# Estimating endogenous treatments effects under long-range dependency without untreated controls

**DOI:** 10.1371/journal.pone.0347847

**Published:** 2026-06-03

**Authors:** Shiming Hao

**Affiliations:** Law School, Yunnan University, Chenggong, Kunming, Yunnan, P. R. China; Indian Statistical Institute, INDIA

## Abstract

The identification and estimation of social policy effects through time‑series natural experiments is fundamental in modern econometrics. However, challenges come from the heterogeneities caused by staggered treatment adoptions and the endogeneities caused by omitted variables. In this paper, we propose a novel method to identify and estimate staggeringly adopted endogenous treatments effects with several treatments when there is no available (suitable) untreated unit and instrument variable. First, we propose a conditional mean symmetry condition by projecting the potential outcomes onto a sub-linear space spanned by the proposed common proximal variable. Under this condition, we can rule out confounding biases. Second, a proposed weak index restriction constructed by Bernstein expansions satisfying conditional mean independence property enables us to consistently estimate multiple heterogeneous treatments effects, and the proposed estimators are robust to weak common proximal variable. We show that the asymptotic distribution of the step-wise estimator is a fractional Brownian motion process with long range dependency. Third, we propose a bootstrap procedure to circumvent the inference difficulty brought by time-series dependencies. Monte Carlo simulations show that our proposed estimator and inference framework work well in small samples, and our contributions are further illustrated by an empirical example with unilateral divorce law reforms.

## Introduction

This paper considers the identification and estimation of the following treatments effects panel model:


$$\begin{array}{cc} y_{it}= & \;S_{it}\beta_{it}+W_{it}\eta_{it}+D_{it}\xi_{it}+X_{it}\alpha_{it}+Z_{it}\nu_{it}+\mu_{i}+\mu_{t}+\epsilon_{it},\\ & \;\mathrm{cov}\left(\lambda_{it},\epsilon_{it}\right)\neq 0,\;\lambda_{it}\in \{S_{it},D_{it}\},\;i=1,2,\ldots ,N;\;t=1,2,\ldots ,T, \end{array}$$ (1)

*y*_*it*_ is a scale response variable for unit *i* at time *t*, i.e., some observed social-economic outcome; *S*_*it*_ is a treatment taking place at time *t*_*Si*_ and driven by the confounder *W*_*it*_, *D*_*it*_ is another treatment taking place at time *t*_*Di*_ and driven by the confounder *X*_*it*_, e.g., the treatments (*S*_*it*_, *D*_*it*_) may be some economic stimulate policies promoted by the governments while the confounders (*W*_*it*_, *X*_*it*_) may be some social-economic factors driving the growth of *y*_*it*_ and the stimulation of the policies (*S*_*it*_, *D*_*it*_) [1–4]. We assume that *W*_*it*_ and *X*_*it*_ are all of dimension one. *Z*_*it*_ are *d*_*Z*_ dimensional exogeneous control variables which are independent of the treatments (*S*_*it*_, *D*_*it*_) and the model error $\epsilon_{it}$. $\mu_{i}$ and $\mu_{t}$ are individual fixed effect and time fixed effect. The parameters of interest are the heterogeneous treatment effects $\beta_{it}$ and $\xi_{it}$. We consider three main challenges that commonly arise in empirical studies:

1. **The endogeneity problem.** We assume that the confounders (*W*_*it*_, *X*_*it*_) are unobservable. Meanwhile, we relax the Gauss-Markov Theorem to allow the treatments to be correlated with the error term: $\mathrm{cov}(\lambda_{it},D_{it})\neq 0$ for $\lambda_{it}\in \{S_{it},D_{it}\}$, which means that even if the confounders are observed and included in model (1), the model is still endogeneous. In empirical studies, Instrument Variables (IVs) approaches are considered to be an effective way to handle the problem under this scenario [5], however good quality IVs are hard to be found [6–8]. Hence, we directly assume in this paper that no suitable IVs are available for empirical analysis.
2. **The heterogeneity problem.** On the one hand, as shown in model (1), all parameters are time-varying and unit-varying (two-way heterogeneous), which implies that treating the treatments effects as constant will lead to false estimations of ATE and ATT [9–11]. On the other hand, we further assume that the treatments (*S*_*it*_, *D*_*it*_) are staggeringly adopted, i.e., there exist at least two $i\neq i^{\prime }\in \{1,2,\ldots ,N\}$ such that $t_{Si}\neq t_{Si^{\prime }}$ and $t_{Di}\neq t_{Di^{\prime }}$. It is well-known that regional policy evaluation approaches such as Two-Way Fixed Effect Estimations (TWFE), Synthetic Control Methods (SCM) and Difference in Difference (DID) among others will lead to false estimations under this scenario [12–19], e.g., the contamination bias [20].
3. **The absence of untreated units.** When the social-economic policies (*S*_*it*_, *D*_*it*_) are one-size-fit-all, e.g., the government’s policies are carried out nationwide, no untreated units are available for identification and estimation of the treatments effects. Regional policy evaluation approaches such as TWFE, SCM and DID among others are no longer applicable under this scenario. Empirical researches tend to find or construct untreated units from historical data or cross-national data (e.g., [21]), however the validities are suspectable. To say the least, even if the polices are not one-size-fit-all, we are stilling facing the problem of selecting “good-quality” untreated units [22]. In fact, adopting unsuitable untreated units will lead to biased estimations [23,24].

Although these three problems are common in empirical studies, the methodological literature handling these three dilemmas simultaneously as far as we know is in straitened circumstances. Most recent discussions have paid attentions to the heterogeneous problem [20,25], however if untreated units are absent, the proposed methods are not applicable. Even if untreated units are present, estimating model (1) by the proposed methods with the endogeneity problem will also lead to biased estimations. How to identify and estimate heterogeneous treatments effects with endogeneity and staggeringly adopted treatments in untreated units and IVs absent time-series natural experiments remains an unsolved problem.

To overcome these difficulties, a novel method is proposed. We propose a Conditional Mean Symmetry condition by projecting the counterfactual outcome onto the sub-linear space spanned by the proposed Common Proximal variable, wherein the Common Proximal variable is of high correlation with the unobservable confounders and independent of the error term. The Conditional Mean Symmetry condition is, on the one hand, shown to be equivalent to the Weak Index Restrictions used in the identification of semiparametric models [26,27]; on the other hand it is also equivalent to the matching condition used in observation studies which rules out confounding biases (see, e.g., [28–31]). Our framework is built on Professor Halbert White and colleague’s pioneering works [32–34], and we further illustrate the relationships between Granger (G-) causality, Sims causality and structural causality in untreated units absent time series natural experiments [35], as well as how we can disentangle one treatment effect from another through a forward point-wise estimation approach based on the proposed Conditional Mean Symmetry condition.

We contribute to the recent emerging literature on heterogeneous treatments effects in event studies with variations in treatments timings in two main fronts. First, we allow non-stationary potential outcomes. Stationarity is usually required in time-series natural experiments to guarantee valid inference [10,23,36,37], however social-economic outcomes are always non-stationary, e.g., trend stationary processes. Causal inference with non-stationary outcomes is somehow nettlesome. In view of this, our proposed bootstrap inference framework allows the potential outcomes to be non-stationary processes, e.g., the potential outcomes are trend-stationary processes with long-range dependency, where the deterministic trend captures the long-term growth and the stationary component follows a long memory model. As far as we are concerned, this is the first time we consider non-stationarity with long range dependency in time-series natural experiments.

Second, the Conditional Mean Symmetry identification assumption proposed in this paper presents an innovative identification paradigm that extends causal inference to empirical settings—such as national policy evaluations—where the DID framework is not applicable due to the absence of a valid comparison group. If there is no untreated unit, [32–34,38,39] and [40] have considered the possibility of using only treated units to estimate causal effects. However, they did not consider scenarios involving multiple endogenous treatments and long-range dependence, both of which are common in real-world data. Our approach fills this gap.

**Fig 1:**
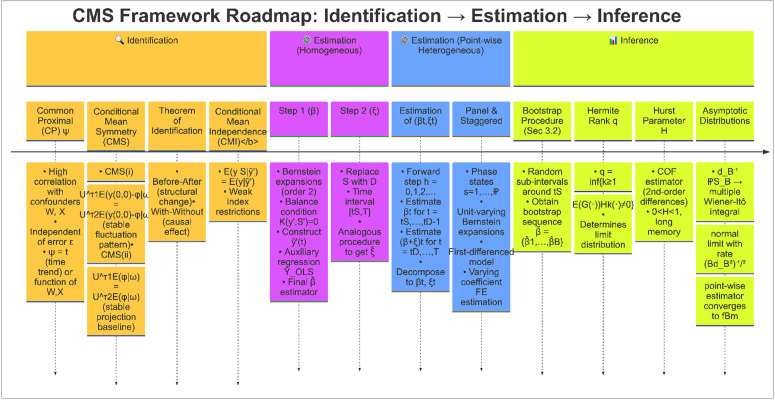
Roadmap of identification, estimation and inference.

The rest of the paper is organized as shown in the roadmap Fig 1. The second part introduces the identification assumptions and describes how we can identify multiple treatments effects without untreated units under the Neyman-Rubin counterfactual framework. The estimation procedures and inference method for a single (univariate) time series model are gathered in the third part, and the fourth part extents the proposed methods to handle panel (multivariate) settings. The fifth part reports the results of the numerical examples and the final part concludes. All the proofs and additional analysis are collected in the Online Supplementary Materials S1 File-S5 File. To facilitate the use of our method, we provide an open-source R package endoLTE at https://github.com/codescollection/endoLTE that implements all estimators and inference procedures proposed in this paper.

## Identification

We will start our identification and estimation approach by a single (univariate) time-series model with homogeneous treatments effects. Then, we will show how the proposed approach can be extended to the heterogeneous settings and staggered panel settings, as shown in model (1).

### Global identification

Consider the following 4 specifications:


$$\begin{array}{c} \text{DGP1:}\;y_{t}=S_{t}\beta_{1}+W_{t}\eta_{1}+D_{t}\xi_{1}+X_{t}\alpha_{1}+Z_{t}\nu_{1}+\epsilon_{t,1},\;W_{t}↔D_{t}, \end{array}$$ (2)

$$\begin{array}{c} \text{DGP2:}\;y_{t}=S_{t}\beta_{2}+W_{t}\eta_{2}+D_{t}\xi_{2}+X_{t}\alpha_{2}+Z_{t}\nu_{2}+\epsilon_{t,2},\;W_{t}⟂D_{t}, \end{array}$$ (3)

$$\begin{array}{c} \text{DGP3:}\;y_{t}=S_{t}\beta_{3}+W_{t}\eta_{3}+Z_{t}\nu_{3}+\epsilon_{t,3}, \end{array}$$ (4)

$$\begin{array}{c} \text{DGP4:}\;y_{t}=D_{t}\xi_{4}+X_{t}\alpha_{4}+\epsilon_{t,4}, \end{array}$$ (5)

(*S*_*t*_, *D*_*t*_) are treatments with the confounders (*W*_*t*_, *X*_*t*_), *Z*_*t*_ are exogenous control variables. DGP1 assumes that the confounder *W*_*t*_ is correlated with the treatment *D*_*t*_, i.e., $W_{t}↔D_{t}$, while DGP2 assumes that *W*_*t*_ is independent of *D*_*t*_. DGP3 and DGP4 respectively describes a single treatment effect model.

**Fig 2:**
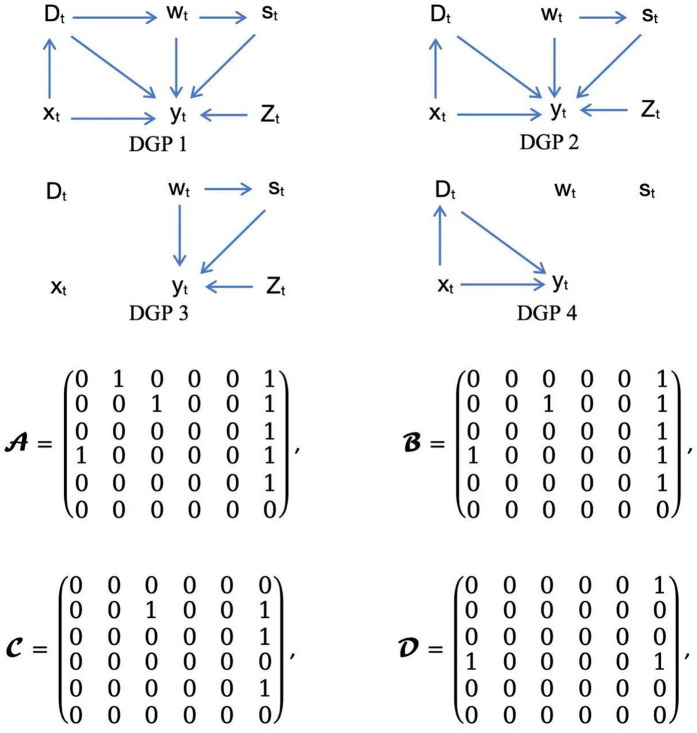
DAGs for the treatments effects models (2-5) (DGPs 1-4) with corresponding adjacency matrices.

The Directed Acyclic Graphs (DAGs) for DGPs (2)–(5) are shown in Fig 2, corresponding to the adjacency matrices 𝒜,ℬ,𝒞,𝒟.

**Definition 1 (Pseudo-subspace).** *If we let* ${ℒ}_{*,n}$ *denote the n-th column of the matrix* ${ℒ}_{T\times N}$*, then there exists a pseudo-subspace spanned by* ${ℒ}_{*,n}$: $k_{1}{ℒ}_{*,1}+k_{2}{ℒ}_{*,2}+\ldots +k_{N}{ℒ}_{*,N}$ *for all nonzero constants* $k_{n}\in \mathbb{R}⧵\{0\}$, n=1,2,…,N*. We denote this pseudo-subspace as* ${ℳ}^{-1}(ℒ)$.

**Assumption 1 (Rank condition)** *In models (2)–(5), for the random vectors* $W=(W_{1},W_{2},\ldots ,W_{T}{)}^{\prime }$, $S=(S_{1},S_{2},\ldots ,S_{T}{)}^{\prime }$, $D=(D_{1},D_{2},\ldots ,D_{T}{)}^{\prime }$, $X=(X_{1},X_{2},\ldots ,X_{T}{)}^{\prime }$*, and the matrix* $Z=(Z_{1},Z_{2},\ldots ,Z_{T}{)}^{\prime }$*, denote* ${𝐱}_{1}=(W,S,D,X,Z)$ *for DGP1,* ${𝐱}_{2}=(W,S,D,X,Z)$ *for DGP2,* ${𝐱}_{3}=(W,S,Z)$ *for DGP3 and* ${𝐱}_{4}=(X,D)$ *for DGP4.* **x**_1_ *and* **x**_2_ *are matrices of dimension* $T\times (4+d_{z})$, **x**_3_ *is of dimension* $T\times (2+d_{z})$ *and* **x**_4_ *is of dimension* *T* × 2*. Then we have* $\mathrm{rank}({𝐱}_{1}^{\prime }{𝐱}_{1})=4+d_{z}$, $\mathrm{rank}({𝐱}_{2}^{\prime }{𝐱}_{2})=4+d_{z}$, $\mathrm{rank}({𝐱}_{3}^{\prime }{𝐱}_{3})=2+d_{z}$ *and* $\mathrm{rank}({𝐱}_{4}^{\prime }{𝐱}_{4})=2$.

The difference between the usual subspace and the pseudo-subspace defined in Definition 1 is that we do not allow *k*_*n*_ = 0, so one can see from the adjacency matrices that ${ℳ}^{-1}(ℬ)={ℳ}^{-1}(C+D)$ while ${ℳ}^{-1}(𝒜)\neq {ℳ}^{-1}(C+D)$. Assumption 1 requires $t_{S}\neq t_{D}$, we do not allow treatments take place at the same time, e.g., the governments’ policies are carried out at the same time. In fact if *t*_*S*_ = *t*_*D*_, treatments $\left(S_{t},D_{t}\right)$ would be mixed with each other, making it impossible to distinguish one from another.

**Proposition 1.** *If Assumption 1 is satisfied, then the treatment effect* β *and the treatment effect* ξ *can be separately identified from each other under DGP2 with the adjacency matrix* ℬ *but not under DGP1 with the adjacency matrix* 𝒜.

### Why and how it is possible to identify treatment effect in untreated units absent time-series natural experiments

As shown in Proposition 1, model (2) is unidentifiable, hence only the following treatments effects model (6) is identifiable. We now start with this univariate time-series model with homogeneous coefficients, and show how the estimation and inference procedure could be extended to handle heterogeneous panel settings latter.


$$\begin{array}{c} y_{t}=W_{t}\eta +S_{t}\beta +D_{t}\xi +X_{t}\alpha +Z_{t}\gamma +\varepsilon_{t},\;\mathrm{cov}(\lambda_{t},\varepsilon_{t})\neq 0,\;t=1,2,\ldots ,t_{S},\ldots ,t_{D},\ldots ,T, \end{array}$$ (6)

where $\lambda_{t}\in \{S_{t},D_{t}\}$, $W_{t}⟂D_{t}$  *Z*_*t*_ are exogenous control variables independent of $\lambda_{t}$ and $\varepsilon_{t}$. The parameters of interest are (β,ξ), but (*D*_*t*_, *S*_*t*_) are endogenous due to the unobservability of (*W*_*t*_, *X*_*t*_) and $\mathrm{cov}(\lambda_{t},\varepsilon_{t})\neq 0$. Instead of finding IVs for the endogenous treatment variables *D*_*t*_ and *S*_*t*_, we propose to find (or construct) a Common Proximal Variable $\psi_{t}$ satisfying the following Assumptions 2–3 and Definition 2. On this basis, for model (6), we consider the Bernstein expansions of the random variables $y_{t}\equiv y(t)$, $S_{t}\equiv S(t)$ and $D_{t}\equiv D(t)$ on the Common Proximal Variable $\psi_{t}$ of order 2 [41]:


$$\begin{array}{c} y(t)=\sum_{k=0}^{2}\frac{2!}{k!(2-k)!}\psi_{t}^{k}(1-\psi_{t}{)}^{2-k}f_{t}\;\left(\frac{k}{2}\right)=\rho_{y,2}\psi_{t}^{2}+\rho_{y,1}\psi_{t}+\rho_{y,0}, \end{array}$$ (7)

$$\begin{array}{c} \lambda (t)=\sum_{k=0}^{2}\frac{2!}{k!(2-k)!}\psi_{t}^{k}(1-\psi_{t}{)}^{2-k}f_{t}\;\left(\frac{k}{2}\right)=\rho_{\lambda ,2}\psi_{t}^{2}+\rho_{\lambda ,1}\psi_{t}+\rho_{\lambda ,0},\;\lambda \in \{S,D\}, \end{array}$$ (8)

where $f_{\lambda }(0)=0$, $f_{\lambda }(\frac{1}{2})=\frac{1}{2}\rho_{\lambda ,1}$, $f_{\lambda }(1)=\rho_{\lambda ,2}+\rho_{\lambda ,1}$, $\rho_{y,2},\rho_{y,1},\rho_{\lambda ,2},\rho_{\lambda ,1}\in \mathbb{R}⧵\{0\}$ are called Bernstein coefficients and the expansions (7)–(8) we get are called Bernstein polynomials of y(t),λ(t) on $\psi_{t}$, where λ(t)∈{S(t),D(t)}. Note that the first order derivative of *y*(*t*) and λ(t) with respect to $\psi_{t}$ are


$$\begin{array}{c} y^{\prime }(t)=2\rho_{y,2}\psi_{t}+\rho_{y,1},\;\lambda^{\prime }(t)=2\rho_{\lambda ,2}\psi_{t}+\rho_{\lambda ,1}. \end{array}$$

**Assumption 2** *(i) The random variables W and* ψ *are of high correlation, X and* ψ *are also of high correlation, e.g.,* ψ *is a proximal variable for W and X. (ii)* ψ *is independent of* ε*, and* E(ε)=0

**Assumption 3** *There exists a continuous function* $g\in C^{p}$, *p* ≥ 1*, such that: (i)* $W_{t}=ag(y^{\prime }(t))+b+u_{t}$, $X_{t}=cg(y^{\prime }(t))+d+w_{t}$, a,b,c,d∈ℝ⧵{0}, *C*^*p*^ *is the Lebesgue space* $C^{p}(\Omega_{g},{ℱ}_{g},\mu_{g})$ *with* $\|g{\|}_{p}=(\int |g{|}^{p}\;d\mu_{g}{)}^{1/p}$ *defined on* $\Omega_{g}$, *u*_*t*_, *w*_*t*_ *are martingale processes independent of* $\psi_{t}$ *and E(u) = 0, E(w) = 0. (ii) For the Bernstein expansions, (W,X) and* $g(y^{\prime })$ *are of high correlations respectively, where* $g(y^{\prime })=(g(y^{\prime }(1)),g(y^{\prime }(2)),\ldots ,g(y^{\prime }(T)))$*. (iii) The composite error* $u_{t}+w_{t}+\varepsilon_{t}$ *is independent of* $\lambda_{t},\psi_{t}$ *and* *Z*_*t*_*, where* $\lambda_{t}\in \{S_{t},D_{t}\}$.

**Definition 2 (Common Proximal Variable).** *For model (6), a random variable* $\psi_{t}$ *satisfying Assumptions 2–3 is called a Common Proximal Variable (CP).*

**Remark 1 (Doob decomposition theorem).** *For most of the social-economic outcomes* *y*_*t*_*, if* *y*_*t*_ *is changing with time, i.e.,* *y*_*t*_ *is a function of t, as we defined in (7); or if there are theoretical and empirical evidences supporting that* *W*_*t*_, *X*_*t*_ *and* *Z*_*t*_ *are changing with time, we would then take* $\psi_{t}=t$ *satisfying Assumptions 2–3 without loss of generality. Particularly, if both* $(W_{t},{ℱ}_{W_{t}}{)}_{t\in \tau }$ *and* $(X_{t},{ℱ}_{X_{t}}{)}_{t\in \tau }$ *are sub-martingales for* τ={1,2,…,T}*, and we have*


$$\begin{array}{c} W_{t}=ag(\psi_{t})+b+u_{t},\;X_{t}=cg(\psi_{t})+d+w_{t}\;\text{for }\psi_{t}=t, \end{array}$$ (9)

*where* $g(\cdot )\in {ℒ}^{p}$ *is a continuous and non-decreasing function, the coefficients are real non-zero numbers* a,b,c,d∈ℝ⧵{0}*, E(u)=E(w)=0, then (9) is exactly the famous Doob-Meyer decomposition: the sub-martingales* *W*_*t*_ *and* *X*_*t*_ *can be decomposed into a non-decreasing process* $g(\psi_{t})$ *plus martingales* *u*_*t*_ *and* *w*_*t*_ *respectively by Assumption 3(i).*

Following [42] and [43], we let $y_{t}(S,D)$ denote the potential outcome under different realizations of S,D∈{1,0}, so we observe $y_{t}(0,0)$ for 1 ≤ *t* < *t*_*S*_, $y_{t}(1,0)$ for $t_{S}\leq t<t_{D}$ and $y_{t}(1,1)$ for *t*_*D*_ ≤ *t* < *T*. Then *y*_*t*_ in (6) can be rewritten as


$$\begin{array}{c} y_{t}=y_{t}(0,0)\cdot 𝕀\{1\leq t<t_{S}\}+y_{t}(1,0)\cdot 𝕀\{t_{S}\leq t<t_{D}\}+y_{t}(1,1)\cdot 𝕀\{t_{D}\leq t<T\} \end{array}$$ (10)

for any t∈{1,…,T}. $y_{t}(0,0)$ for $t_{S}\leq t<t_{D}$ then denotes the counterfactual outcome if there is no treatment *S*_*t*_; $y_{t}(0,0)$ for $t_{D}\leq t<t_{T}$ denotes the counterfactual outcome if there are no treatments (*S*_*t*_, *D*_*t*_); $y_{t}(0,1)$ for $t_{D}\leq t<t_{T}$ denotes the counterfactual outcome if there is no treatment *S*_*t*_ [44,45].

**Definition 3 (Sub-sample expectation operator on the Hilbert space).** *Let* $ℋ\subset {ℒ}^{p}(\Omega_{m},{ℱ}_{m},\mu_{m})$ *be the Hilbert space generated by the random process* $\{m_{t}{\}}_{t\in \tau }$, ℋ *contains all the functions* $\sum_{t\in \ell }c_{t}m_{t}$ *for* ℓ⊂τ={1,2,…,T} *and* $c_{t}\in ℛ$*. Define a family of operators* $U^{\ell }$ *on* ℋ *as* $U^{\ell }E(m)=\frac{1}{\left|\ell \right|}\sum_{t\in \ell }m_{t}$, |ℓ| *is the size of the set ℓ. Then* $U^{\ell }E(\cdot )$ *is called the sub-interval expectation operator on the sub-space* ℓ⊂τ={1,2,…,T}.

**Definition 4 (Mean symmetry and conditional mean symmetry).** *For any random variable* $x\in {ℒ}^{p}(\Omega_{x},{ℱ}_{x},\mu_{x})$*, if* $U^{\tau_{1}}E(x)=U^{\tau_{2}}E(x)$ *for* $\tau_{1},\tau_{2}\subset \tau =\{1,2,\ldots ,T\}$ *where* $\tau_{1}\cap \tau_{2}=\emptyset$, $\tau_{1}\cup \tau_{2}=\tau$*, then we say that x satisfies mean symmetry. The conditional mean symmetry is then defined by projecting x onto another random variable* $y\in {ℒ}^{p}(\Omega_{y},{ℱ}_{y},\mu_{y})$ *such that* $U^{\tau_{1}}E(x|y)=U^{\tau_{2}}E(x|y)$.

**Assumption 4 (Long range dependency)** *In model* (6), $\mathrm{cov}(\varepsilon_{t+n},\varepsilon_{t})\sim H(2H-1)n^{-2(1-H)}$ *as* n→∞ *for* t=1,2,…,T *and* T→∞*, the parameter* H∈(0,1) *is referred to as the Hurst parameter,* $\varepsilon_{t}$ *is stationary.* *W*_*t*_, *X*_*t*_ *and* *Z*_*t*_ *are stationary or trend stationary processes, hence* *y*_*t*_ *is a stationary or trend stationary process with long range dependency.*

**Assumption 5 (Conditional Mean Symmetry, CMS)** *There exist series of random sequences* $\phi =\{\phi_{1},\phi_{2},\ldots ,\phi_{T}\}$ *and* $\omega =\{\omega_{1},\omega_{2},\ldots ,\omega_{T}\}$ *such that: (i) conditional parallel:* $U^{\tau_{1}}E(y(0,0)-\phi |\omega )=U^{\tau_{2}}E(y(0,0)-\phi |\omega )$*, where* $y(0,0)=\{y_{1}(0,0),y_{2}(0,0),\ldots ,y_{T}(0,0)\}$ *is the counterfactual outcome sequence; and (ii) conditional mean symmetry:* $U^{\tau_{1}}E(\phi |\omega )=U^{\tau_{2}}E(\phi |\omega )$*, where* $\tau_{1}=\{1,2,\ldots ,t_{\lambda }-1\}$, $\tau_{2}=\{t_{\lambda },t_{\lambda }+1,\ldots ,T\}$, λ∈{S,D}.

If we let $\chi_{t}=y_{t}(0,0)-\phi_{t}$ be a martingale process with respect to the σ-filtration $\sigma (y_{r}(0,0)-\phi_{r},y_{r-1}(0,0)-\phi_{r-1},\ldots ,y_{1}(0,0)-\phi_{1})$, *r* ≤ *t*, then $\{\chi_{t}{\}}_{t\in \tau }$ satisfies Assumption 5(i). This is because $E(\chi_{t}|\sigma (\chi_{r},\ldots ,\chi_{1}))=\chi_{r}$ and $E(\chi_{t})=E(\chi_{r})$, hence we can get Assumption 5(i) with $\omega =(y_{r}(0,0)-\phi_{r},y_{r-1}(0,0)-\phi_{r-1},\ldots ,y_{1}(0,0)-\phi_{1}{)}^{\prime }$. Under this scenario, Assumption 5(ii) is also satisfied. To see this, suppose that there exists an affine function $h\in {𝒞}^{p}(\Omega_{h},{ℱ}_{h},\mu_{h})$ such that E(ϕ∣ω)=h(ω). Note that if $\omega_{t}$ is a stationary process, by the von-Neumann Ergodic Theorem (see [46,47]), define $\phi^{-}=\{\phi_{t+k}{\}}_{t\in \ell }$ and $\omega^{-}=\{\omega_{t+k}{\}}_{t\in \ell }$, we can get


$$\begin{array}{c} \lim_{|\ell |\to \infty }U^{\ell }E(\phi |\omega )=\lim_{|\ell |\to \infty }U^{\ell }E(\phi^{-}|\omega^{-})=\rho \in ℋ,\text{ for any }k\in \mathbb{Z}, \end{array}$$

which exactly implies Assumption 5(ii) with $\ell =\{1,2,\ldots ,t_{\lambda }-1\}$ and $k=t_{\lambda }-1$. Assumption 5 indicates that $\chi_{t}$ is an ergodic but non-mixing process on the Hilbert space [48]. This implies that the random sequence $\phi =(\phi_{1},\;\phi_{2},\;\ldots ,\;\phi_{T}{)}^{\prime }$ could be chosen by satisfying the condition that $(\chi_{t},{ℱ}_{\chi_{t}}{)}_{t\in \tau }$ is a martingale process with $\chi_{t}=y_{t}(0,0)-\phi_{t}$, e.g., the random sequences ϕ could be the untreated units as shown in Fig 2 (grey solid lines). This remind us that, in time-series natural experiments, one of the criterions of judging whether the selected untreated units are “good enough” is that the difference between the counterfactual outcome and the untreated unit should be a martingale process over the whole time interval.

**Fig 3:**
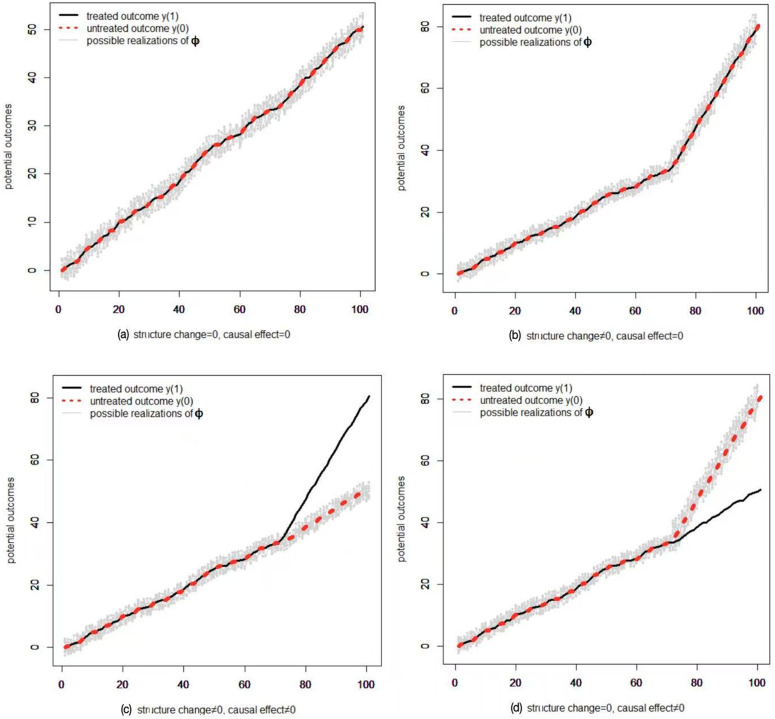
Comparisons between structure change and treatment effect through hypothetical examples (a-d).

For time series, structure change (break) is usually defined as a *before-after change*, i.e., there is a spontaneous trend, slope, structure or regime change before and after some point of time; while treatment effect is defined as a *with-without change*, i.e., the growth path of the observed *y*_*t*_ with *D*_*t*_ (or *S*_*t*_), ceteris paribus, would be totally different without *D*_*t*_ (or *S*_*t*_). Hence treatment effect is defined in a counterfactual way, while structure change or other distribution shifts effect is not [28–30,49,50]. This is also the main difference between Granger Causality, Sims Causality and the Neyman-Rubin counterfactual framework (e.g., [35,38,51,52]). Fig 3 illustrates the differences through hypothetical DGPs. It can be seen that without further restrictions (assumptions), structure change has no direct relationship with treatment effect. A (non-)zero treatment effect does not necessarily imply a (non-)zero structure change, and vice versa. Empirical researchers may hold the intuition that non-zero treatment effect implies non-zero structure change, as illustrated in Fig 3c. However, this is not always the case, see Fig 3b and (d) for examples.

**Fig 4:**
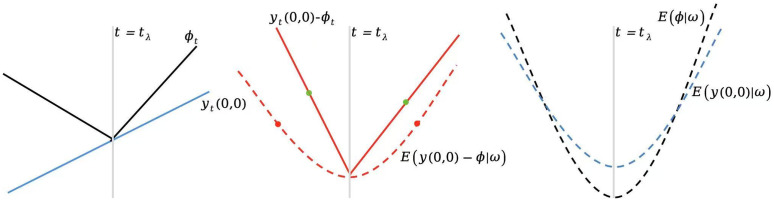
Hypothetical illustration example of Conditional Mean Symmetry (Assumption 5). the middle panel shows Assumption 5 **(i)**, the right panel shows Assumption 5 (i) and **(ii)**. The blue solid line shows the original (before-projection) counterfactual outcome $y_{t}(0,0)$, the black solid line shows the original (before-projection) random sequence $\phi_{t}$, while the blue dashed line shows the after-projection counterfactual outcome E(ϕ(0,0)∣ω) and the black dashed line shows the after-projection sequence E(ϕ∣ω). The red solid line shows the difference of before-projections $y_{t}(0,0)-\phi_{t}$, while the red dashed line shows the difference of after-projections E(y(0,0)−ϕ∣ω). The green dots show the sub-sample means of before-projections: $U^{\tau_{1}}E(y(0,0)-\phi )$ and $U^{\tau_{2}}E(y(0,0)-\phi )$, while the red dots show the sub-sample means of after-projections: $U^{\tau_{1}}E(y(0,0)-\phi |\omega )$ and $U^{\tau_{2}}E(y(0,0)-\phi |\omega )$, $\tau_{1}=\{1,2,\ldots ,t_{\lambda }-1\}$, $\tau_{2}=\{t_{\lambda },t_{\lambda }+1,\ldots ,T\}$, λ∈{S,D}. The vertical line shows the time when the treatment takes place.

**Fig 5:**
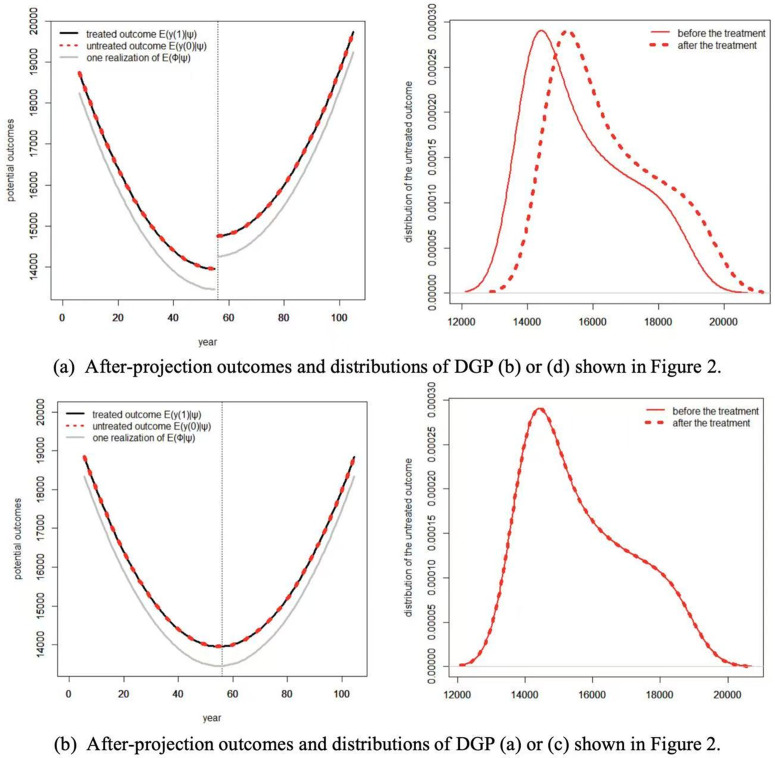
After-projection outcomes (left panel), and distributions of DGPs (b) and (c) shown in Fig 3 (right panel). We take $\omega_{t}=\psi_{t}$. For the left panel, the dark solid line shows the after-projections of the treated outcome E{Y(1)|ω}, the red dashed line shows the after-projections of the untreated outcome E{Y(0)|ω}, and the grey solid line shows one possible realization of E{ϕ|ω}. For the right panel, the red solid line shows the distribution of the untreated outcome (after-projection) for the pre-treatment period $t=1,2,\ldots ,t_{S}-1$, while the red dashed line shows the distribution of the untreated outcome (after-projection) for the treatment period $t=t_{S},t_{S}+1,\ldots ,T$. The vertical line shows the time when the treatment takes place. For expositional convenience, we only consider model (6) with single treatment *S*_*t*_.

To make the interpretations and credibility of our assumptions clear and transparent, an intuitive illustration of Assumption 5 is further given in Fig 4 and 5, it can be seen that the random sequences $y_{t}(0,0)$ and $\phi_{t}$ will not satisfy Assumption 5 (the green dots do not equal to each other in Fig 4) until we project them on to the space spanned by (the red dots equal to each other), where we take $\omega_{t}=\psi_{t}$. Note that in this paper we allow the observed outcome to be non-stationary (e.g., trend stationary process), while the literatures usually require (conditional) stationary for identification and inference in time-series natural experiments, see, e.g., [53,54].

**Remark 2 (Matching condition)** *Assumption 5 rules out confounding biases caused by* (*W*_*t*_, *X*_*t*_) *and guarantees what identified are pure treatments effects. To see this, consider the DGPs (a-d) shown in* Fig 3*, the after-projections of the potential outcomes are shown in Fig 5 respectively. It can be seen that the DGP (b) or (d) does not satisfy Assumption 5(ii) (Conditional Mean Symmetry fails down due to the fact that there exists a structure change), and neglecting the distribution shifts shown in the right panel of* Fig 5(a) *will lead to false positive identification results, i.e., the identified effect stems from structural shifts, which has been misleadingly interpreted as evidence of a treatment effect; or false negative mistake, just as shown in DGP (b) or (d) of Fig 3 which satisfies Assumption 5(i) but not (ii).*

Assumption 5 implicitly implies that the treatment effect can be identified if and only if the distribution of the untreated outcome (after-projection) for the pre-treatment period shares a common support with that of the treatment period (Fig 3a and (c) shown in Fig 5b). As stressed by [28] and [32], reliable estimates obtained when the support for the treated regime coincides with or is a subset of that for the non-treated regime, and not necessarily otherwise. This is the core Matching Condition used in observational studies (e.g., [28,29]). Assumption 5 then permits us to avoid confounding the effect of treatment with shifts in the distribution of other causes.

**Remark 3.** *Recall the general panel data model* $y_{it}=g(B_{it},\alpha_{i},\epsilon_{it})$*, the time-homogeneity condition assumes that* $\epsilon_{it}|B_{it},\alpha_{i}\to_{d}\epsilon_{is}|B_{is},\alpha_{i}$ *for all* t,s∈{1,2,…,T}*, where* *B*_*it*_ *is the explanatory variable and* $\to_{d}$ *denotes distributional equivalence, the disturbance* $\epsilon_{it}$ *is then conditional strict stationary* [55,56]. *When* $B_{it}\in \{0,1\}$*, the time-homogeneity condition, under our scenario* (6)*, is equivalent to the assumption that the conditional distribution of the counterfactual untreated outcome* $y_{t}(0,0)$ *given* (*X*_*t*_, *W*_*t*_) *does not change before and after the treatment, as shown in the right panel of* Fig 5(b). *However, Assumption 5 does not necessarily imply the time-homogeneity condition, so Assumption 5 is less restrictive. Hence, with the distribution of factors other than* *D*_*t*_ *(or* *S*_*t*_*) not varying over time, the changes in* *D*_*t*_ *(or* *S*_*t*_*) over time can help identify the ceteris paribus effect of* *D*_*t*_ *(or* *S*_*t*_*) on* *y*_*t*_*, this is exactly why and how we can achieve causal inference in time-series natural experiment through Assumption 5.*

**Assumption 6 (SUTVA and no anticipation)** *We assume stable unit treatment value (SUTVA) and no anticipation effect exist.*

**Theorem 1 (Identifications).** *For model (6) with* $\tau_{4}=\{t_{D},t_{D}+1,\ldots ,T\}$, $\tau_{3}=\{1,2,\ldots ,t_{D}-1\}$, $\tau_{2}=\{t_{S},t_{S}+1,\ldots ,t_{D}-1\}$ *and* $\tau_{1}=\{1,2,\ldots ,t_{S}-1\}$*: (i) Before-After (structure change effect): if Assumption 5(i) is satisfied, then we have*


$$\begin{array}{c} \beta +\xi =U^{\tau_{4}}E(y(1,1)|S,D,\omega )-U^{\tau_{3}}E(y(0,0)|S,D,\omega ), \end{array}$$

$$\begin{array}{c} \xi =U^{\tau_{4}}E(y(1,1)|S,D,\omega )-U^{\tau_{2}}E(y(1,0)|S,D,\omega ), \end{array}$$

$$\begin{array}{c} \beta =U^{\tau_{2}}E(y(1,0)|S,D,\omega )-U^{\tau_{1}}E(y(0,0)|S,D,\omega ). \end{array}$$

*(ii) With-Without (treatment effect): if Assumptions 5(i)-(ii) are both satisfied, we will then get*


$$\begin{array}{c} \beta +\xi =U^{\tau_{4}}E(y(1,1)-y(0,0)|S,D,\omega ), \end{array}$$

$$\begin{array}{c} \xi =U^{\tau_{4}}E(y(1,1)-y(1,0)|S,D,\omega ), \end{array}$$

$$\begin{array}{c} \beta =U^{\tau_{2}}E(y(1,0)-y(0,0)|S,D,\omega ). \end{array}$$

*The Before-After part (i) identifies the structure change effect, while the With-Without part (ii) identifies the treatment effect.*

We are now able to give a formal distinction between treatment effect and structure change effect:

**Definition 5 (Causal field and multiverse).** *For model (6), the random processes triplets* (*y*_*t*_, *D*_*t*_, *X*_*t*_) *is called a causal field where E(y|D,X) = E(y|X) and* E(yD)≠E(y)E(D)*; while the random process triplets* (*y*_*t*_, *D*_*t*_, *W*_*t*_) *is called a structure change field where* E(y|D,W)≠E(y|D) *and* E(yD)≠E(y)E(D)*. Similarly, the triplet* (*y*_*t*_, *S*_*t*_, *W*_*t*_) *is a causal field and the triplet* (*y*_*t*_, *S*_*t*_, *X*_*t*_) *is a structure change field. For the pairs* (*y*_*t*_, *S*_*t*_) *and* (*y*_*t*_, *D*_*t*_) *without further information, we cannot tell whether they are causal fields or structure change fields. The quadruplets* (*y*_*t*_, *D*_*t*_, *S*_*t*_, *X*_*t*_) *is called a time-bowl (the quadruplets* (*y*_*t*_, *D*_*t*_, *S*_*t*_, *W*_*t*_) *is also a time-bowl), the fields under all time-bowls constitute a multiverse:*


$$\begin{array}{c} \{(y_{t},D_{t},X_{t}),(y_{t},D_{t},W_{t}),(y_{t},S_{t},X_{t}),(y_{t},S_{t},W_{t})\}. \end{array}$$

The interesting *time-bowl* and *multiverse* concepts are borrowed from the 2023 DC movie The Flash. The *time-bowl* corresponds to a specific universe and the *multiverse* is constructed by different specific universes, the Flash Man (the protagonist of the movie) can enter any *time-bowl* from the *multiverse* by his superpower. I’m inspired by these originalities, we econometricians are just like the Flash Man whose superpower are different model assumptions under which we can disentangle one *time-bowl* (e.g., causal field) from another *time-bowl* (e.g., structure change filed).

It can be seen from Theorem 1 and Definition 5 that the parameters of interest (ξ,β) in model (6) have different meanings under different assumptions. The before-after part under Assumption 5(i) identifies a structure change effect, while the with-without part under Assumptions 5(i)-(ii) identifies a causal effect. This finding implies that the parallel assumption alone in DiD settings is not sufficient enough to disentangle treatment effect from structure change effect. The presence of a non-zero estimated effect under the parallel trends assumption does not necessarily imply a causal treatment effect. An alternative explanation is that the treated units were undergoing a spontaneous structural change, while the untreated units were not. Under Assumption 5, a (non-)zero treatment effect implies a (non-)zero structure change as shown in Fig 3(a) and (c), but not vice versa as shown in Fig 3(b) and (d). In observation studies, the triplet (*y*_*t*_, *D*_*t*_, *X*_*t*_) or (*y*_*t*_, *S*_*t*_, *W*_*t*_) describes an “economic story”, which ascribes a causal meaning to the empirical findings. Without such a “story” (the relationship between *y_t_* and *D_t_* (*S_t_*) given *X_t_* (*W_t_*)), we can hardly claim that what we finally get is actually a causal relationship.

### The plausibility of the CMS assumption

To justify the Conditional Mean Symmetry (CMS) assumption and demonstrate its plausibility, we illustrate its logic step-by-step using a specific economic example: the impact of a national minimum wage policy on employment. Suppose a national minimum wage policy (the treatment variable *D*_*t*_) was enacted on January 1, 2000. We want to evaluate the causal effect of this policy on the youth employment rate (*y*_*t*_). This is a classic time-series natural experiment with no untreated units, as the entire country is affected.

The challenging we are facing now is that the employment rate is influenced not only by the policy but also by numerous unobservable confounding factors, such as: (i) Macroeconomic cycles: Employment is high during economic booms and low during recessions. (ii) Industrial structure adjustments: An increase in the service sector’s share and a decrease in manufacturing also affect overall employment. (iii) Demographic changes: Changes in the proportion of the youth population. These factors simultaneously influence whether the policy might be considered (as economic pressures form the backdrop for policy enactment) and the employment outcome.

**Step 1: Finding the “Common Proximal Variable (CP)”** $\psi_{t}$. According to Remark 1, under specific economic scenarios, we can take $\psi_{t}=t$ (the time trend) as the CP variable. The rationale for this choice is that the confounding factors (economic cycles, industrial structure, demographics) all change systematically over time. By Assumptions 2–3, we can use a function of time (e.g., g(·)) to roughly capture these trends, i.e., we assume that the macroeconomic condition *W*_*t*_ can be decomposed as $W_{t}=ag(\psi_{t})+b+u_{t}$, and the industrial structure *X*_*t*_ can be decomposed as $X_{t}=cg(\psi_{t})+d+w_{t}$. Here, $g(\psi_{t})$ is a smoothly varying function of time, while *u*_*t*_ and *w*_*t*_ are short-term random fluctuations around this trend and are independent of the error term $\varepsilon_{t}$.

**Step 2: Applying CMS(i) (Conditional Parallelism).** In our example, CMS(i) implies that *y*_*t*_(0) is the employment rate “if there were no minimum wage policy”. $\phi_{t}$ is the “shadow variable” used for adjustment. It can be understood as the part of the outcome highly correlated with the CP variable $\left(\psi_{t}\right)$. We can simplify this to the part of the employment rate explained by the time trend. $\omega_{t}$ here can be constructed from $y^{\prime }(t)$.

The economic meaning of CMS(i) is: After removing the part explained by the time trend (and the confounding factors associated with it) $\left(\phi_{t}\right)$, the behavior (e.g., its expected value) of the remaining “pure random shock” part $\left(y_{t}(0)-\phi_{t}\right)$ is the same before $\left(\tau_{1}\right)$ and after $\left(\tau_{2}\right)$ the policy implementation. In layman’s terms: Without the policy, employment would fluctuate due to economic cycles. CMS(i) assumes that the “pattern” of these fluctuations is the same before and after the policy. For example, during an economic upturn, employment would be *x*% above the trend line; during a downturn, it would be *y*% below the trend line. This “pattern of deviation from the trend” is stable.

**Step 3: Applying CMS(ii) (Mean Symmetry).** In our example, $\phi_{t}$ can be roughly understood as the “potential employment level driven by the long-term trend”. CMS(ii) assumes that: The employment level determined by the long-term trend itself did not undergo a structural break around the time of the policy. That is, we cannot say that the “long-term trend line” itself had a discontinuity in its slope or intercept right after January 1, 2000, compared to before. The trend line is continuous and smooth across the policy implementation point.

In this specific example, the plausibility of the CMS assumption depends on judgment of the economic context:

- *Plausibility of CMS(i)*: The assumption that the “pattern of deviation of employment from its long-term trend” is stable is a common hypothesis in macroeconomics. Business cycles (boom-recession-recovery) do exhibit a certain regularity and cyclicality [2]. As long as there are no other major structural changes (e.g., war, technological revolution), this pattern of fluctuation around the trend is likely to be similar.
- *Plausibility of CMS(ii)*: The assumption that the “long-term trend itself” did not have a structural break around the policy implementation is precisely what we need to verify. The purpose of the minimum wage policy is to change the employment level, so it could very well cause a change in the intercept or slope of the trend line. CMS(ii) essentially states that the “trend” part captured by $\phi_{t}$ does not include the effect of the policy. The entire policy effect is intended to be captured by the parameter we ultimately estimate, ξ.

The CMS assumption is a stronger assumption than the parallel trends assumption used in DiD settings in that it attempts to substitute cross-unit comparability with within-unit stability over time; however, it is also more flexible because it does not require the existence of a unit unaffected by the treatment.

### Empirical manifestations of CMS violations

Understanding how CMS assumptions would be violated in real-world data is crucial for applied researchers. Below, I describe what empirical violations would look like for each component of the CMS assumption, using concrete economic examples.

#### Violations of CMS(i):

- *Pattern 1: Time-varying volatility in residuals.* Imagine plotting the residuals $Y_{t}-{\hat{Y}}_{t}$ (where ${\hat{Y}}_{t}$ is the projection onto the CP variable) over time. A violation of CMS(i) would show: For the pre-treatment period, residuals fluctuate within a stable range (e.g., ±1%); while for the post-treatment period, the pattern of fluctuations changes fundamentally—perhaps they become more volatile, or the auto-correlation structure changes. In the minimum wage example, suppose before 2000, employment deviations from trend were mild and short-lived (2–3 quarters). After 2000, these deviations become persistent and last 5–7 years. This suggests the underlying economic dynamics have changed, violating the “same fluctuation pattern” assumption.
- *Pattern 2: Structural break in the relationship with the CP.* Another violation occurs if the relationship between the counterfactual untreated outcome and the CP variable changes over time. This would appear as: For the pre-treatment period, E(Y(0,0)|ω) follows a function K(ω); however for the post-treatment period, the function shifts to P(ω)≠K(ω). Consider the divorce law reform example from the later empirical analysis part of the paper. If the relationship between divorce rates and time (the CP variable) fundamentally changed after the 1997 Asian financial crisis—not just a level shift but a change in how divorce responds to economic conditions—this would violate CMS(i). The “adjustment” $\phi_{t}$ that worked before the crisis would no longer be appropriate after.

#### Violations of CMS(ii):

- *Pattern 1: Level shifts in the projected component.* The $\phi_{t}$ series shows a discrete jump exactly at the treatment time that cannot be explained by the treatment itself. In the minimum wage case, suppose $\phi_{t}$ captures the “long-term trend” in employment driven by demographics. If a new demographic survey methodology was introduced in 2000 that redefined how youth population is measured, the trend line would shift mechanically—violating CMS(ii) even if the minimum wage policy had no effect.
- *Pattern 2: Slope changes in the projected component.* The rate of change of $\phi_{t}$ shifts at the treatment boundary. In the divorce law example, suppose the CP variable is time *t*, capturing the natural upward trend in divorce rates due to secular social changes. If the 1997 financial crisis permanently accelerated the pace of social change (making people more individualistic faster), then the slope of $\phi_{t}$ would increase after 1997. This would violate CMS(ii) because the $\phi_{t}$ used to adjust pre-treatment data would not be appropriate post-treatment.
- *Pattern 3: Correlation between* $\phi_{t}$ *and the treatment indicator.* $\phi_{t}$ becomes correlated with *D*_*t*_ or *S*_*t*_ after controlling for $\omega_{t}$. This would appear as: In a regression of $\phi_{t}$ on *D*_*t*_ and $\omega_{t}$, the coefficient on *D*_*t*_ is statistically significant. This indicates that the “pure trend” component is actually contaminated by the treatment itself.

In summary, the violations of CMS(i) come from: units learn from the treatment and respond differently to subsequent shocks; the treatment fundamentally alters how the economy/society responds to other factors; the set of units changes over time (in panel settings); and treatment effects spill over to affect the untreated state. The violations of CMS(ii) would come from: other policy changes or shocks coinciding with the treatment; the treatment itself alters the long-term trends it was supposed to be independent of; and selecting a proximal variable that is itself affected by the treatment.

### Diagnose of the CMS assumption

When untreated units (control units) are present, the random sequence $\phi_{t}$ could be selected as the untreated units, and the CMS assumption could be tested by the empirical process-based methods proposed by [57], and apply them to our constructed residual term $y_{t}(0,0)-\phi_{t}$ on the pre-treatment period $\left[1,t_{s^{*}}\right]$ where we assume $t_{s^{*}}<t_{S}<t_{D}$. Truncate the sample to the period $\tau_{1}=[1,t_{0}-1]$ and $\tau_{2}=[t_{0},t_{s}^{*}]$ (the “faked pre- and post-treatment” interval), if the estimated pseudo effect $\Delta_{-}(i)=U^{\tau_{1}}E(y(0,0)-\phi |\omega )-U^{\tau_{2}}E(y(0,0)-\phi |\omega )$ or $\Delta_{-}(ii)=U^{\tau_{1}}E(\phi |\omega )-U^{\tau_{2}}E(\phi |\omega )$ is significantly nonzero, then CMS is not satisfied. However, when untreated units (control units) are absent, CMS could not be tested directly. Two approaches can be adapted to construct possible indirect tests for CMS:

1. Placebo tests using a “fake treatment time.” We know no actual treatment occurred on the “faked pre- and post-treatment” interval $\tau_{1}$ and $\tau_{2}$, so the true treatment effect should be zero. Apply the paper’s two-step estimation procedure to this hypothetical interval to estimate the “treatment effect.” If we obtain estimates that are statistically significantly different from zero, this provides strong evidence that the CMS assumption (especially CMS(i)) might be violated. Because even in the absence of treatment, our method falsely identifies an “effect,” suggesting the projection mechanism used to construct the counterfactual is biased.
2. Testing implications of Conditional Mean Independence (CMI). Theorem 3 of the paper states that the proposed two-step estimation procedure implies conditional mean independence: $E(YS|\hat{Y})=E(Y|\hat{Y})$. This means that, conditional on ${\hat{Y}}_{t}$, the treatment variable *S*_*t*_ should be unrelated to potential outcomes. While potential outcomes are unobservable, we can test whether *S*_*t*_ is related to some functions of ${\hat{Y}}_{t}$. In the sample including the true pre-treatment period ($\tau_{1}$) and the post-treatment period ($\tau_{2}$), we can test whether $E(S_{t}|{\hat{Y}}_{t})$ is constant. A non-constant relationship would indicate a violation of CMI and thus CMS.

#### Choice of the CP variable.

(i) $\psi_{t}$ should be a sufficient statistics of the confounders or should be of high-correlation with the confounders. This guarantees that the CP variable is not weak.
(ii) $\psi_{t}$ should be independent of $\varepsilon_{t}$, which guarantees that the CP variable is valid.

These two requirements imply that for trend stationary social-economic outcome *y*_*t*_, the CP could be selected as (i) time *t*; or (ii) common factor *f*_*t*_ extracted from $Z_{it}=\mu_{t}+\lambda_{i}f_{t}+e_{it}$, where *Z*_*it*_ are multiple macroeconomic variables (such as GDP, consumption, and investment across regions) correlated with *y*_*t*_ but independent of the policy, $f_{t}$ then is a good CP variable as a measure of the “economic cycle”.

Researchers should select CP variable by integrating economic theory with the context of the research question. Table 1 provides some concrete examples.

**Table 1:** Candidate Common Proximal variables for typical empirical settings.

Treatment/Outcome	Unobserved Confounders	Candidate CP variable	Rationale
The effect of Minimum Wage Policy on youth employment rate	Macroeconomic conditions (business cycle), industrial structure, demographic trends	Time trend or GDP growth of trading partners	Time captures long-run trends; external GDP is unaffected by domestic policy but correlated with domestic cycles
The effect of education reform on students’ test scores	Teaching quality, family background, school resources	Regional GDP per capita (if exogenous to education policy)	GDP reflects economic development that influences both education inputs and student performance
The effect of environmental regulation on air quality	Industrial activity, weather patterns, technology adoption	Industrial production index of major trading partners	External demand drives local industrial activity but may be exogenous to local environmental policy
The effect of healthcare policy on life expectancy	Nutrition, lifestyle, sanitation	Caloric intake per capita (if largely driven by income trends)	Nutrition is a fundamental determinant of health and evolves with economic growth, but may be correlated with policy if policy affects income

The table provides examples of candidate Common Proximal variables for various treatment-outcome pairs, along with the unobservable confounders they aim to capture and the rationale for their choice.

Time $\left(\psi_{t}=t\right)$ is a natural and convenient CP because many confounders trend over time. However, there are several scenarios where time fails to satisfy the CP assumptions: (i) Treatment-induced structural break in the trend: If the treatment itself alters the long-term trajectory of the confounders (or the outcome’s relationship with time), then using a simple time trend violates CMS(ii); (ii) Non-linear or non-smooth confounding: If confounders evolve in a way that cannot be captured by a smooth function of time (e.g., abrupt shifts due to wars, natural disasters, or technological breakthroughs), a simple time trend will not proxy them well. The residuals *u*_*t*_ and *w*_*t*_ would then be correlated with time, violating the martingale difference assumption; (iii) Multiple confounders with different trends: When there are several confounders with different time profiles (e.g., one increasing linearly, another following a U-shape), a single time trend cannot simultaneously capture all. The CP would be misspecified; (iv) Seasonality or high-frequency fluctuations: In quarterly or monthly data, time may not capture seasonal patterns or business cycle frequencies that act as confounders. In such cases, a more sophisticated CP (e.g., a business cycle indicator) is needed; (v) When the treatment timing coincides with another aggregate shock: If the treatment date coincides with a major event (e.g., a financial crisis), the time trend alone cannot distinguish between the treatment effect and the crisis effect. The crisis would appear as a structural break in the trend, violating CMS(i) and (ii).

Multiple CPs are admissible, and researchers can attempt to select the “best” one—or a combination of them. However, within the specific CMS framework of this paper, the theory is developed for a univariate CP $\psi_{t}$. If multiple CPs are available, the researcher faces a model selection problem: choosing which variable (or which combination) best satisfies the CMS assumption. This can be left for future research.

### Estimation by weak index restrictions and inference with long range dependency

Substitute (9) into model (6) and directly estimate model (6) will not be correct due to the fact that $\mathrm{cov}(\lambda_{t},\varepsilon_{t})\neq 0$, $\lambda_{t}\in \{S_{t},D_{t}\}$. In this section, we propose a novel method to deal with this issue without IVs.

#### A two-step estimation approach by weak index restrictions.

**Step 3.1. (Estimation of β)** Based on the Bernstein expansions (7)–(8), we will get


$$\begin{array}{c} y^{\prime }(t)=2\rho_{y,2}\psi_{t}+\rho_{y,1},\;S^{\prime }(t)=2\rho_{S,2}\psi_{t}+\rho_{S,1}\;\text{for }t=1,2,\ldots ,t_{S},\ldots ,t_{D}-1. \end{array}$$

By symbolic computations, see [58,59], there exists a continuous function $K(\cdot ,\cdot )\in C^{2}(\mathbb{R})$ such that the relationship between $y^{\prime }(t)$ and $S^{\prime }(t)$ satisfies:


$$\begin{array}{c} K(y^{\prime }(t),S^{\prime }(t))=a_{S,2}y^{\prime }(t)-a_{y,2}S^{\prime }(t)+a_{y,2}a_{S,1}-a_{S,2}a_{y,1}=0\;\text{with }a_{\sigma ,b}=b\rho_{\sigma ,b}, \end{array}$$

$\rho_{\sigma ,b}$ is the Bernstein coefficient defined in (7)–(8) for b∈{1,2} and σ∈{y,S}. Estimate (7)–(8) by OLS to get the estimators ${\hat{a}}_{\sigma ,b}=b{\hat{\rho }}_{\sigma ,b}$ respectively for *b* and σ, and input all the estimators into the equation $K(y^{\prime }(t),S^{\prime }(t))$, we will get


$$\begin{array}{c} {\hat{y}}^{\prime }(t)=({\hat{a}}_{y,2}S^{\prime }(t)+{\hat{a}}_{S,2}{\hat{a}}_{y,1}-{\hat{a}}_{y,2}{\hat{a}}_{S,1})/{\hat{a}}_{S,2}. \end{array}$$

Then further consider the following auxiliary regression:


$$\begin{array}{c} Y=XB+W, \end{array}$$ (11)

$$\begin{array}{c} {\hat{Y}}_{OLS}=X{\hat{B}}_{OLS}=X(X^{\prime }X{)}^{-1}X^{\prime }Y, \end{array}$$ (12)

$$\begin{array}{c} X=(I\;{\hat{y}}^{\prime }{)}_{(t_{D}-1)\times 2},\;B=(\delta_{0}\;\delta_{1}{)}^{\prime }, \end{array}$$ (13)

$$\begin{array}{c} I=(1,1,\ldots ,1{)}_{(t_{D}-1)\times 1}^{\prime }, \end{array}$$ (14)

$$\begin{array}{c} Y=(y_{1},y_{2},\ldots ,y_{t_{D}-1}{)}^{\prime }, \end{array}$$ (15)

$$\begin{array}{c} {\hat{y}}^{\prime }=({\hat{y}}^{\prime }(1),{\hat{y}}^{\prime }(2),\ldots ,{\hat{y}}^{\prime }(t_{D}-1){)}^{\prime }, \end{array}$$ (16)

*W* is an unobserved random shock. Like Indirect Inference [60], we do not require ${\hat{B}}_{OLS}$ to be consistent for *B* in the auxiliary regression (11). Substitute (6) into ${\hat{Y}}_{OLS}$, we will then get


$$\begin{array}{c} U^{\tau_{2}}E({\hat{Y}}_{OLS})-U^{\tau_{1}}E({\hat{Y}}_{OLS})=(U^{\tau_{2}}E(\hat{y})-U^{\tau_{1}}E(\hat{y}))(\beta \frac{U^{\tau }E(S\hat{y})-U^{\tau }E(S)U^{\tau }E(\hat{y})}{U^{\tau }E(\hat{y}\hat{y})-U^{\tau }E(\hat{y})U^{\tau }E(\hat{y})} \end{array}$$

$$\begin{array}{c} +\;\eta \frac{U^{\tau }E(W\hat{y})-U^{\tau }E(W)U^{\tau }E(\hat{y})}{U^{\tau }E(\hat{y}\hat{y})-U^{\tau }E(\hat{y})U^{\tau }E(\hat{y})}+\alpha \frac{U^{\tau }E(X\hat{y})-U^{\tau }E(X)U^{\tau }E(\hat{y})}{U^{\tau }E(\hat{y}\hat{y})-U^{\tau }E(\hat{y})U^{\tau }E(\hat{y})} \end{array}$$

$$\begin{array}{c} +\;\gamma \frac{U^{\tau }E(Z\hat{y})-U^{\tau }E(Z)U^{\tau }E(\hat{y})}{U^{\tau }E(\hat{y}\hat{y})-U^{\tau }E(\hat{y})U^{\tau }E(\hat{y})}+\frac{U^{\tau }E(\varepsilon \hat{y})-U^{\tau }E(\varepsilon )U^{\tau }E(\hat{y})}{U^{\tau }E(\hat{y}\hat{y})-U^{\tau }E(\hat{y})U^{\tau }E(\hat{y})}) \end{array}$$

$$\begin{array}{c} \equiv \beta {𝒜}_{1}+\eta {𝒜}_{2}+\alpha {𝒜}_{3}+\gamma {𝒜}_{4}+{𝒜}_{5}=\beta {𝒜}_{1}+\eta {𝒜}_{2}+\alpha {𝒜}_{3}+\gamma {𝒜}_{4} \end{array}$$

for $\tau_{1}=\{1,2,\ldots ,t_{S}-1\}$, $\tau_{2}=\{t_{S},\ldots ,t_{D}-1\}$ and $\tau =\{1,2,\ldots ,t_{D}-1\}$, the last equality holds true (${𝒜}_{5}=0$) by (6) and Assumption 2 (ii). Notice that by Assumption 3, model (6) could be rewritten as


$$\begin{array}{c} y_{t}=(a\eta +c\alpha )g(y^{\prime }(t))+S_{t}\beta +D_{t}\xi +Z_{t}\gamma +e+u_{t}+w_{t}+\varepsilon_{t},\;\mathrm{cov}(\lambda_{t},\varepsilon_{t})\neq 0, \end{array}$$ (17)

where e=bη+dα, $\lambda_{t}\in \{S_{t},D_{t}\}$, $t=1,2,\ldots ,t_{D}-1$, thereby we can get


$$\begin{array}{c} U^{\tau_{2}}E({\hat{Y}}_{OLS})-U^{\tau_{1}}E({\hat{Y}}_{OLS})=\beta {𝒜}_{1}+(a\eta +c\alpha )(U^{\tau_{2}}E(\hat{y})-U^{\tau_{1}}E(\hat{y}))+\gamma {𝒜}_{4}. \end{array}$$

Estimate (17) by semiparametric methods, under Assumption 2 (ii) we will get $\hat{\left(a\eta +c\alpha \right)}=(a\eta +c\alpha )+o_{p}(1)$ and $\hat{\gamma }=\gamma +o_{p}(1)$ as $t_{D}-1\to \infty$, see, e.g., [61]. Substitute these estimators into $U^{\tau_{2}}E({\hat{Y}}_{OLS})-U^{\tau_{1}}E({\hat{Y}}_{OLS})$, we will finally get


$$\begin{array}{c} \hat{\beta }=\frac{U^{\tau_{2}}E({\hat{Y}}_{OLS})-U^{\tau_{1}}E({\hat{Y}}_{OLS})-\hat{\left(a\eta +c\alpha \right)}(U^{\tau_{2}}E(\hat{y})-U^{\tau_{1}}E(\hat{y}))-\hat{\gamma }{𝒜}_{4}}{{𝒜}_{1}}. \end{array}$$

**Assumption 6** *As* $t_{D}-1\to \infty$*, we have (i)* $\frac{t_{D}-t_{S}}{t_{D}-1}=o_{p}(1)$;

*(ii)* $\sum_{t=t_{S}}^{t_{D}-1}Z_{t}<\infty$ *and* $\sum_{t=t_{S}}^{t_{D}-1}y_{t}<\infty$;
*(iii)* $U^{\tau }E(y^{2})<\infty$, $U^{\tau }E(Z^{2})<\infty$, $U^{\tau }E(yZ)<\infty$, $U^{\tau }E(y)<\infty$ *and* $U^{\tau }E(Z)<\infty$;
*(iv)* $\sum_{t=1}^{t_{D}-1}S_{t}(u_{t}+w_{t}+\varepsilon_{t})<\infty$, $\tau =\{1,2,\ldots ,t_{D}-1\}$.

**Theorem 2.** *(UNBIASEDNESS AND CONSISTENCY) Under the Assumptions 1–5 and Assumption 6,* $E(\hat{\beta })=\beta$*, and* $\hat{\beta }=\beta +o_{p}(1)$.

**STEP 3.2. (ESTIMATION OF** ξ) The estimation approach is similar to Step 3.1 except for replacing the time interval $\left[1,t_{D}-1\right]$ in (11) with $\left[t_{S},T\right]$, and replacing $S^{\prime }(t)$ with $D^{\prime }(t)$. The estimator we finally get is


$$\begin{array}{c} \hat{\xi }=\frac{U^{\tau_{4}}E({\hat{Y}}_{OLS})-U^{\tau_{1}}E({\hat{Y}}_{OLS})-\hat{\beta }{\tilde{𝒜}}_{1}-\hat{\left(a\eta +c\alpha \right)}(U^{\tau_{4}}E(\hat{y})-U^{\tau_{3}}E(\hat{y}))-\hat{\gamma }{\tilde{𝒜}}_{3}}{{\tilde{𝒜}}_{4}}, \end{array}$$

where $\tau_{3}=\{t_{S},t_{S}+1,\ldots ,t_{D}-1\}$, $\tau_{4}=\{t_{D},t_{D}+1,\ldots ,T\}$ and $\tau =\{t_{S},t_{S}+1,\ldots ,T\}$, ${\hat{Y}}_{OLS}=X{\hat{B}}_{OLS}=X(X^{\prime }X{)}^{-1}X^{\prime }Y$ with $X=(I\;\hat{y}{)}_{(T-t_{S}+1)\times 2}$, $B=(\delta_{0}\;\delta_{1}{)}^{\prime }$, $I=(1,1,\ldots ,1{)}_{(T-t_{S}+1)\times 1}^{\prime }$, $Y=(y_{t_{S}},y_{t_{S}+1},\ldots ,y_{T}{)}^{\prime }$, and $\hat{y}=({\hat{y}}^{\prime }(t_{S}),{\hat{y}}^{\prime }(t_{S}+1),\ldots ,{\hat{y}}^{\prime }(T){)}^{\prime }$. The coefficients are


$$\begin{array}{c} {\tilde{𝒜}}_{1}=(U^{\tau_{4}}E(\hat{y})-U^{\tau_{1}}E(\hat{y}))\frac{U^{\tau }E(S\hat{y})-U^{\tau }E(S)U^{\tau }E(\hat{y})}{U^{\tau }E(\hat{y}\hat{y})-U^{\tau }E(\hat{y})U^{\tau }E(\hat{y})}, \end{array}$$

$$\begin{array}{c} {\tilde{𝒜}}_{3}=(U^{\tau_{4}}E(\hat{y})-U^{\tau_{3}}E(\hat{y}))\frac{U^{\tau }E(Z\hat{y})-U^{\tau }E(Z)U^{\tau }E(\hat{y})}{U^{\tau }E(\hat{y}\hat{y})-U^{\tau }E(\hat{y})U^{\tau }E(\hat{y})}, \end{array}$$

$$\begin{array}{c} {\tilde{𝒜}}_{4}=(U^{\tau_{4}}E(\hat{y})-U^{\tau_{3}}E(\hat{y}))\frac{U^{\tau }E(D\hat{y})-U^{\tau }E(D)U^{\tau }E(\hat{y})}{U^{\tau }E(\hat{y}\hat{y})-U^{\tau }E(\hat{y})U^{\tau }E(\hat{y})}. \end{array}$$

Under the Assumptions 1–5, by Theorem 1 and similar to Theorem 2, we can get $E(\hat{\xi })=\xi$, and $\hat{\xi }=\xi +o_{p}(1)$ as long as conditions similar to these in Assumption 6 are satisfied, i.e., (i) $\frac{T-t_{D}}{t_{D}-t_{S}}=o_{p}(1)$;

(ii) $\sum_{t=t_{D}}^{T}Z_{t}<\infty$ and $\sum_{t=t_{D}}^{T}y_{t}<\infty$;
(iii) $U^{\tau }E(y^{2})<\infty$, $U^{\tau }E(Z^{2})<\infty$, $U^{\tau }E(yZ)<\infty$, $U^{\tau }E(y)<\infty$ and $U^{\tau }E(Z)<\infty$;
(iv) $\sum_{t=t_{S}}^{T}D_{t}(u_{t}+w_{t}+\varepsilon_{t})<\infty$, $\tau =\{t_{S},t_{S}+1,\ldots ,T\}$.

The following statements clarifies why and how the above proposed estimation approach satisfies Assumption 5 and guarantees Theorem 1:

**Theorem 3.** *(CONDITIONAL MEAN INDEPENDENCE, CMI) For the 2-steps estimation approach, we have* $E(𝒴S|{\hat{y}}^{\prime })=E(𝒴|{\hat{y}}^{\prime })$*, where* ${𝒴}_{t}=(y_{t}(1,0),y_{t}(0,0))$ *for Step 3.1; and* $E(𝒴D|{\hat{y}}^{\prime })=E(𝒴|{\hat{y}}^{\prime })$*, where* $𝒴=(y_{t}(1,1),y_{t}(0,0))$ *for Step 3.2.*

Interestingly, Theorem 3 indicates that given ${\hat{y}}^{\prime }(t)$, which is constructed by the CP $\psi_{t}$, the treatments (*S*_*t*_, *D*_*t*_) are nearly randomly assigned, which is a weak version of the *unconfoundness condition* $\left(y_{t}(1,1),y_{t}(0,0))⟂\lambda_{t}|{\hat{y}}^{\prime }(t\right)$ for $\lambda_{t}\in \{S_{t},D_{t}\}$.

**Remark 4.** *(WEAK INDEX RESTRICTIONS) Theorem 3 is natural in the sense that the CP* $\psi_{t}=h(W_{t},X_{t})$ *is an index function of the confounders* (*W*_*t*_, *X*_*t*_) *by Assumptions 2–3. The conditions* E(π|X)=E(π|ψ) *and* E(π|W)=E(π|ψ)*, in which* π=u+w+ε *is the error term defined in model (17), are the well known weak or mean index restrictions widely used in semiparametric identifications (*[62–64]*). Under weak index restrictions, the treatments effects can be uniquely identified and consistently estimated even if untreated units and IVs are not available.*

**Remark 5.** *(CMI IMPLIES CMS) To see how CMI implies CMS, for expositional purposes, we consider model (6) with* β=0 *(Remark 5 is still valid for the case where* β≠0*. But for convenience, we only consider the single treatment situation), and we assume that* $t_{D}=T/2$*. For Assumption 5 (ii), we take* $\omega_{t}=\psi_{t}$*. Because* ${\hat{y}}^{\prime }(t)$ *is a continuous and measurable function of* $\psi_{t}$*, hence* $\omega_{t}={\hat{y}}^{\prime }(t)$ *also satisfies Assumption 5 (ii). For Assumption 5 (i), note that*


$$\begin{array}{c} {\hat{y}}^{\prime }(t)=({\hat{a}}_{y,2}S^{\prime }(t)+{\hat{a}}_{S,2}{\hat{a}}_{y,1}-{\hat{a}}_{y,2}{\hat{a}}_{S,1})/{\hat{a}}_{S,2}\equiv \alpha (t)\cdot t+\beta (t) \end{array}$$

*for some* α(t), $\beta (t)\in C^{2}(\mathbb{R})$*, hence one can verify that* ${\hat{y}}^{\prime }(t\in \tau_{1})=a\cdot {\hat{y}}^{\prime }(t\in \tau_{2})+b$ *for a = 1 and* $b=2\rho_{y,2}\cdot |\tau_{2}|$*, where* ${\hat{y}}^{\prime }(t\in \tau_{1})=({\hat{y}}^{\prime }(1),{\hat{y}}^{\prime }(2),\ldots ,{\hat{y}}^{\prime }(t_{D}-1){)}^{\prime }$ *and* ${\hat{y}}^{\prime }(t\in \tau_{2})=({\hat{y}}^{\prime }(t_{D}),{\hat{y}}^{\prime }(t_{D}+1),\ldots ,{\hat{y}}^{\prime }(T){)}^{\prime }$*. By the proof of Theorem 3, we can get* $y_{t}(0,0)=a(t)\cdot {\hat{y}}^{\prime }(t)+b(t)+\varepsilon_{t}$ *for some a(t),* $b(t)\in C^{2}(\mathbb{R})$*, and* E(ε)=0*. Substitute* $y_{t}(0,0)$ *into Assumption 2 (i), we will then get*


$$\begin{array}{c} U^{\tau_{j}}E(y(0,0)|{\hat{y}}^{\prime })=a(t\in \tau_{j})\cdot {\hat{y}}^{\prime }(t\in \tau_{j})+b(t\in \tau_{j}),\;j=1,2. \end{array}$$

*After simplifications, by Assumption 5 (i),*


$$\begin{array}{cc} & U^{\tau_{2}}E(y(0,0)|{\hat{y}}^{\prime })-U^{\tau_{1}}E(y(0,0)|{\hat{y}}^{\prime })\\ & \;=\{a\cdot a(t\in \tau_{2})-a(t\in \tau_{1})\}\cdot {\hat{y}}^{\prime }(t\in \tau_{1})\\ & \;\;+\{b\cdot a(t\in \tau_{2})+b(t\in \tau_{2})-b(t\in \tau_{1})\}=0 \end{array}$$

*if and only if the following balance conditions are satisfied:*


$$\begin{array}{c} a\cdot a(t\in \tau_{2})-a(t\in \tau_{1})=0\;\text{and}\;b\cdot a(t\in \tau_{2})+b(t\in \tau_{2})-b(t\in \tau_{1})=0. \end{array}$$

*Simultaneously considering Theorem 3 and the above balance conditions, CMI implies CMS if and only if*


$$\begin{array}{cc} & a(t\in \tau_{2})-a(t\in \tau_{1})=0,\\ & 2\rho_{y,2}\cdot |\tau_{2}|\cdot a(t\in \tau_{2})+b(t\in \tau_{2})-b(t\in \tau_{1})=0,\\ & U^{\tau_{2}}E(y(1,1))-a(t\in \tau_{2})\cdot U^{\tau_{2}}E({\hat{y}}^{\prime })-b(t\in \tau_{2})-c(t\in \tau_{2})\cdot U^{\tau_{2}}E(D)=0,\\ & U^{\tau_{1}}E(y(1,1))-a(t\in \tau_{1})\cdot U^{\tau_{1}}E({\hat{y}}^{\prime })-b(t\in \tau_{1})-c(t\in \tau_{1})\cdot U^{\tau_{1}}E(D)=0, \end{array}$$

*where* $U^{\tau_{2}}E(D)=1$ *and* $U^{\tau_{1}}E(D)=0$; $c(t\in \tau_{2})$ *and* $c(t\in \tau_{1})$ *are defined in the proof of Theorem 3 (see the online Supplementary Material* S2 File*),* $\tau_{1}=\{1,2,\ldots ,t_{D}-1\}$, $\tau_{2}=\{t_{D},t_{D}+1,\ldots ,T\}$*. The above simultaneous functions are equivalent to*


$$\begin{array}{c} (\begin{array}{cccc} 1 & 0 & \Sigma & \Omega \\ 0 & 1 & -1 & 0\\ 0 & 0 & 1 & 1\\ -\Xi & 0 & 0 & 1 \end{array})(\begin{array}{c} a(t\in \tau_{2})\\ b(t\in \tau_{1})\\ b(t\in \tau_{2})\\ c(t\in \tau_{2}) \end{array})=(\begin{array}{c} \Pi \\ \Phi \\ \Psi \\ \Gamma \end{array}),\;\text{which we denote as }X\theta =\Delta , \end{array}$$

*where*


$$\begin{array}{cc} \Xi & =1/\{2\beta_{y,2}\cdot |\tau_{2}|-(U^{\tau_{2}}E({\hat{y}}^{\prime })-U^{\tau_{1}}E({\hat{y}}^{\prime }))\},\\ \Omega & =U^{\tau_{2}}E({\hat{y}}^{\prime }),\\ \Phi & =U^{\tau_{1}}E(y(1,1)),\\ \Pi & =-\{U^{\tau_{2}}E(y(1,1))-U^{\tau_{1}}E(y(1,1))\}/\{2\beta_{y,2}\cdot |\tau_{2}|-(U^{\tau_{2}}E({\hat{y}}^{\prime })-U^{\tau_{1}}E({\hat{y}}^{\prime }))\},\\ \Psi & =U^{\tau_{1}}E({\hat{y}}^{\prime }),\\ \Sigma & =2\beta_{y,2}\cdot |\tau_{2}|,\\ \Gamma & =U^{\tau_{2}}E(y(1,1)). \end{array}$$

*Under this scenario one can verify that, by some elementary but tedious computations,* $\mathrm{rank}(X^{\prime }X)=4$*, hence* θ *can be uniquely point identified and estimated by* $(X^{\prime }X{)}^{-1}X^{\prime }\Delta$*. This implies that CMI is compatible with CMS, there is an equivalent relationship between CMI and CMS.*

### Inference with long range dependency

We consider a bootstrap procedure. For the estimation Step 3.1, consider divide the whole time interval $\left[1,2,\ldots ,t_{S},\ldots ,t_{D}-1\right]$ into sub-intervals $I_{b}=[t_{s,b},t_{s,b}+1,\ldots ,t_{S},\ldots ,t_{e,b}]$ around *t*_*S*_ randomly, where $t_{s,b}\in [1,t_{S}-1)$ and $t_{e,b}\in [t_{S},t_{D}-1]$ for b=1,2,…,B. On each interval, we can get ${\hat{\beta }}_{b}$ by Step 3.1. Thereby, by Theorem 2, the bootstrap sequence $\tilde{B}=\{{\hat{\beta }}_{1},{\hat{\beta }}_{2},\ldots ,{\hat{\beta }}_{B}\}$ should be an i.i.d. stationary process with $E({\hat{\beta }}_{b})=\mu_{\beta }$. The parameter of interest is $\sigma_{\beta }^{2}=\mathrm{Var}({\hat{\beta }}_{b})$. By [65] and [66], the limit behavior of the partial sum ${PS}_{B}=\sum_{b=1}^{B}(M_{b}-\sigma_{\beta }^{2})$ depends on the Hermite rank of the function *G*, where $M_{b}=({\hat{\beta }}_{b}-\mu_{\beta }{)}^{2}$ and ${\hat{\beta }}_{b}=G(y_{t\in I_{b}},S_{t\in I_{b}},D_{t\in I_{b}},Z_{t\in I_{b}},\psi_{t\in I_{b}})$. Let $H_{k}(\beta )=(-1{)}^{k}\exp(\frac{1}{2}\beta^{2})\;d^{k}\exp(-\frac{1}{2}\beta^{2})/d\beta^{k}$, β∈ℝ, *k* ≥ 0 denote the *k*-th Hermite polynomial. The Hermite rank *q* of *G* is then defined as $q=\inf\{k\geq 1:E(G(ℛ))H_{k}(ℛ)\neq 0\}$, ℛ=(y,S,D,Z,ψ). Suppose that $\mathrm{cov}({\hat{\beta }}_{b},{\hat{\beta }}_{b^{\prime }})=(b^{\prime }-b{)}^{-H}ℒ(b^{\prime }-b)$ for 0 < *H* < *q*^−1^ and ℒ:(0,∞)→ℝ is a slowly varying function at infinity [67], i.e., $\lim_{t\to \infty }ℒ(\tau k)/ℒ(k)=1$ for all τ>0. Set $d_{B}=B^{2-qH}{ℒ}^{q}(B{)}^{1/2}$, as B→∞ we have


$$\begin{array}{c} d_{B}^{-1}{PS}_{B}{⇝}_{d}\Theta^{-q/2}c_{q}\int_{{\mathbb{R}}^{q}}\frac{\exp(i(B_{1}+\ldots +B_{q}))-1}{i(B_{1}+\ldots +B_{q})}\\ \cdot \prod_{k=1}^{q}|B_{k}{|}^{(H-1)/2}dW(B_{1})\ldots dW(B_{q}) \end{array}$$ (18)

where $\Theta =2\Gamma (H)\cos(\frac{1}{2}H\pi )$, $c_{q}=E(G(ℛ)H_{q}(ℛ))/q!$. $d_{B}^{-1}\mathbb{P}S_{B}$ converges in distribution to a multiple Wiener-Itô integral with respect to the random spectral measure *W* of Gaussian white-noise processes [46,66,68].

For *q* = 1, $d_{B}^{-1}{PS}_{B}$ has a normal limit distribution with mean zero and variance $2c_{q}/(1-H)(2-H)$; and for *q* ≥ 2, the limit distribution is non-normal. Herein, in this paper we chose to reconstruct the normalizing factor *d*_*B*_ so as to get a more straightforward limit distribution. As noted by [68,69], the wrong choice of *d*_*B*_ will make the distribution of the statistics $d_{B}^{-1}{PS}_{B}$ converge to a degenerate limit because $d_{B}^{-1}{PS}_{B}$ tends to zero so fast. An appropriate construction could be ${ℍ}_{B}=(Bd_{B}^{2}{)}^{-1/2}({PS}_{B}-B\cdot \sigma_{\beta ,\eta }^{2})$, then we will get

**THEOREM 4. (Asymptotic of the bootstrap estimator)** *if* *q* ≥ 1 *and* $B^{-1}=o(1)$*, then*


$$\begin{array}{c} \underset{x\in \mathbb{R}}{\sup}\left|P_{B}\left({ℍ}_{B}\leq x\right)-\Phi \left(x/\sigma_{q}\right)\right|=o(1), \end{array}$$

*where* $\sigma_{q}^{2}=2(2-qH{)}^{-1}(1-qH{)}^{-1}c_{q}^{2}q!$, Φ *is the cumulative distribution function of the normal distribution with mean zero and variance 1,* *P*_*B*_ *is the probability measure defined on the bootstrap sequence. Furthermore,*


$$\begin{array}{c} \underset{x\in \mathbb{R}}{\sup}\left|P_{B}\left({ℍ}_{B}\leq x\right)-\Phi \left(d_{B}^{-1}\mathbb{P}S_{B}\leq x\right)\right|=o(1) \end{array}$$

*if and only if q = 1.*

Usually $E(\beta^{2})\neq 0$ for model (6), hence $E(G(ℛ))H_{k=1}(ℛ)\neq 0$ for most of the DGPs, thereby the Hermite rank can be taken as $\hat{q}=1$. On this basis, we propose to estimate the Hurst parameter *H* by using the change-of-frequency (COF) estimator based on the second-order difference of $\tilde{B}$ ([70,71]):


$$\begin{array}{c} \hat{H}=\frac{1}{2}\log_{2}\left(\frac{\sum_{i=1}^{B-4}{\left({\tilde{B}}_{i+4}-2{\tilde{B}}_{i+2}+{\tilde{B}}_{i}\right)}^{2}}{\sum_{i=1}^{B-2}{\left({\tilde{B}}_{i+2}-2{\tilde{B}}_{i+1}+{\tilde{B}}_{i}\right)}^{2}}\right), \end{array}$$

where $\log_{2}(\cdot )$ is the base-2 logarithm. The critical values and confidence interval can be then constructed correspondingly by [72]. The proposed bootstrap procedure can be then applied similarly to the estimation Step 3.2, i.e., we divide the whole time interval [1,2,…,T] into sub-intervals around *t*_*D*_ randomly, then repeat Step 3.1 and Step 3.2 to get the bootstrap sequence $\tilde{B}=\{{\hat{\xi }}_{1},{\hat{\xi }}_{2},\ldots ,{\hat{\xi }}_{B}\}$, the inference framework thereby follows. Be cautious that, our bootstrap inference framework for $\hat{\xi }$ accounts for the estimation uncertainty brought by the first estimation Step 3.1, because $\hat{\xi }$ is a smooth function of $\hat{\beta }$ as shown in Step 3.2.

## Heterogeneous treatments effects and panel settings with staggered treatments adoptions

### A forward point-wise estimation approach of the heterogeneous treatments effects

Consider now the following one-way heterogeneous treatments effects model (the coefficients are time-varying):


$$\begin{array}{c} y_{t}=W_{t}\eta_{t}+S_{t}\beta_{t}+D_{t}\xi_{t}+X_{t}\alpha_{t}+Z_{t}\gamma_{t}+\nu_{t}+\varepsilon_{t},\;t=1,2,\ldots ,t_{S},\ldots ,t_{D},\ldots ,T \end{array}$$ (19)

where $\mathrm{cov}(\lambda_{t},\varepsilon_{t})\neq 0$, $\lambda_{t}\in \{S_{t},D_{t}\}$. All variables and parameters follow previous definitions. The parameters of interests are $\beta_{t}$ for $t=t_{S},\ldots ,t_{D},\ldots ,T$ and $\xi_{t}$ for $t=t_{D},\ldots ,T$.

**STEP 4.1. (Estimation of** $\beta_{t}$ **for** $t=t_{S},\ldots ,t_{D}-1$) We estimate the following model respectively for the forward point-wise step $h=0,1,2,\ldots ,t_{D}-t_{S}-1$:


$$\begin{array}{c} y_{t}-{\hat{\beta }}_{t}^{-}\cdot I\{t\leq t_{S}\}-{\hat{\beta }}_{t}^{+}\cdot I\{t>t_{S}+h\}=W_{t}\eta_{t}+S_{t}\beta_{t}+X_{t}\alpha_{t}+Z_{t}\gamma_{t}+\nu_{t}+\varepsilon_{t}, \end{array}$$ (20)

where $t=1,2,\ldots ,t_{S}-1+h$, ${\hat{\beta }}_{t}^{-}=0$ and ${\hat{\beta }}_{t}^{+}={\hat{\beta }}_{t-h}$. Estimate model (20) by Step 3.1 for $t_{D}-t_{S}-1$ times, we will get point-wise estimators ${\hat{\beta }}_{t_{S}},{\hat{\beta }}_{t_{S}+1},{\hat{\beta }}_{t_{S}+2},\ldots ,{\hat{\beta }}_{t_{D}-1}$ respectively for the point-wise step *h*.

**STEP 4.2. (Estimation of** $\beta_{t}+\xi_{t}$ **for** $t=t_{D},\ldots ,T$) Further consider the following model respectively for the forward point-wise step $h=0,1,2,\ldots ,T-t_{D}$:


$$\begin{array}{c} {\tilde{y}}_{t}=W_{t}\eta_{t}+SD_{t}(\beta +\xi {)}_{t}+X_{t}\alpha_{t}+Z_{t}\gamma_{t}+\nu_{t}+\varepsilon_{t},\;t=1,2,\ldots ,t_{S},\ldots ,t_{D}+h \end{array}$$ (21)

where


$$\begin{array}{c} {\tilde{y}}_{t}=y_{t}\cdot I\{1\leq t\leq t_{S}-1\}+(y_{t}-{\hat{\beta }}_{t}\cdot I\{t_{S}\leq t\leq t_{D}-1\})+y_{t}\cdot I\{t=t_{D}\}+(y_{t}-{\hat{\left(\beta +\xi \right)}}_{t}^{+})\cdot I\{t>t_{D}+h\}, \end{array}$$

$SD_{t}\equiv S_{t}D_{t}$ is the total treatment indicator, $(\beta +\xi {)}_{t}\equiv \beta_{t}+\xi_{t}$ is the total treatment effect, ${\hat{\left(\beta +\xi \right)}}_{t}^{+}={\hat{\left(\beta +\xi \right)}}_{t-h}$. In accordance with Step 4.1, estimate model (21) by Step 3.1 for *T* − *t*_*D*_ times, we will get point-wise estimators ${\hat{\left(\beta +\xi \right)}}_{t_{D}},{\hat{\left(\beta +\xi \right)}}_{t_{D}+1},\ldots ,{\hat{\left(\beta +\xi \right)}}_{T}$ for the point-wise step *h* respectively.

**STEP 4.3. (Estimation of** $\beta_{t}$ **and** $\xi_{t}$ **for** $t=t_{D},\ldots ,T$) Combining Step 4.1 and 4.2 to get $\hat{\phi }\equiv ({\hat{\beta }}_{t_{S}},{\hat{\beta }}_{t_{S}+1},\ldots ,{\hat{\beta }}_{t_{D}-1},{\hat{\left(\beta +\xi \right)}}_{t_{D}},{\hat{\left(\beta +\xi \right)}}_{t_{D}+1},\ldots ,{\hat{\left(\beta +\xi \right)}}_{T})$, consider the following forward one step-wise regression model


$$\begin{array}{c} \hat{\phi }-{\hat{\theta }}_{t}^{-}\cdot I\{t\leq t_{D}\}-{\hat{\theta }}_{t}^{+}\cdot I\{t>t_{D}+h\}=D_{t}\theta_{t}+\sigma_{t}\;\text{for }t=t_{S},t_{S}+1,\ldots ,t_{D}-1+h, \end{array}$$

where ${\hat{\theta }}_{t}^{-}=0$, ${\hat{\theta }}_{t}^{+}={\hat{\theta }}_{t-h}$, $h=0,1,2,\ldots ,T-t_{D}$. Estimate this model by Step 3.1 for *T* − *t*_*D*_ times, we will finally get the estimators


$$\begin{array}{c} {\hat{\xi }}_{t_{D}}={\hat{\theta }}_{t_{D}},\;{\hat{\xi }}_{t_{D}+1}={\hat{\theta }}_{t_{D}+1},\;{\hat{\xi }}_{t_{D}+2}={\hat{\theta }}_{t_{D}+2},\;\ldots ,\;{\hat{\xi }}_{T}={\hat{\theta }}_{T} \end{array}$$

and


$$\begin{array}{c} {\hat{\beta }}_{t_{D}}={\hat{\left(\beta +\xi \right)}}_{t_{D}}-{\hat{\xi }}_{t_{D}},\;{\hat{\beta }}_{t_{D}+1}={\hat{\left(\beta +\xi \right)}}_{t_{D}+1}-{\hat{\xi }}_{t_{D}+1},\;\ldots ,\;{\hat{\beta }}_{T}={\hat{\left(\beta +\xi \right)}}_{T}-{\hat{\xi }}_{T} \end{array}$$

respectively for the point-wise step *h*.

Let $\zeta_{t}$, t∈ℤ be *i.i.d.* random variables and ${ℱ}_{t}=(\ldots ,\zeta_{t-1},\zeta_{t})$ be the corresponding sigma-filtration. For model (19) with ${𝒳}_{t}=(g({\hat{y}}^{\prime }(t)),S_{t},Z_{t})$, and $\pi_{t}$ defined in Remark 4, we assume that ${𝒳}_{t}=𝔾(t/T,{ℱ}_{t})$, $\pi_{t}=ℍ(t/T,{ℱ}_{t})$, ${𝒳}_{t}\pi_{t}=GH(t/T,{ℱ}_{t})$ and $y^{\prime }(t)\pi_{t}=JH(t/T,{ℱ}_{t})$, 𝔾 and ℍ are measurable functions such that $𝔾(t/T,{ℱ}_{t})$ and $ℍ(t/T,{ℱ}_{t})$ are well defined for each *t* and $E(\pi |y^{\prime },Z,S)=0$ (see [73–75] for further definitions). The variance-covariance matrix of these random processes can be denoted as:


$$\begin{array}{c} {ℳ}_{𝒳}(t)=E(𝔾{𝔾}^{\prime }),\;{ℳ}_{\pi }(t)=E(ℍ{ℍ}^{\prime }),\;{ℳ}_{𝒳\pi }(t)=E(GH{GH}^{\prime }),\;{ℳ}_{y^{\prime }\pi }(t)=E(JH{JH}^{\prime }). \end{array}$$

We now construct the asymptotic behavior of the point-wise estimator $\hat{\beta }(\tau )$, the asymptotic behavior of $\hat{\xi }(\tau )$ can be similarly constructed, hence omitted here for brevity.

**Theorem 5. (Asymptotics of the point-wise estimator)** *Assume that the functions* 𝔾, ℍ, 𝔾ℍ, 𝕁ℍ *are stochastically Lipschitz continuous processes, and the smallest eigenvalues of the matrices* ${ℳ}_{𝒳}(t)$, ${ℳ}_{\pi }(t)$, ${ℳ}_{𝒳\pi }(t)$ *and* ${ℳ}_{y^{\prime }\pi }(t)$ *are bounded away from zero. Define* $\hat{\beta }(\tau )={\hat{\beta }}_{t_{S}-1+\tau }$ *respectively for* $\tau =1,2,\ldots ,T-t_{S}+1$*, then we have*


$$\begin{array}{cc} (t_{S}+\tau -1{)}^{H} & (\hat{\beta }(\tau )-\beta (\tau )-\mathrm{Bias}(\tau )){⇝}_{d}\\ & \frac{1}{{\overset{ˇ}{A}}_{1}}\mathrm{Co}{\left(\int_{0}^{r_{\tau }T}{ℳ}_{𝒳}(s)\;ds\right)}^{\;-1}(\mathbb{Q}(r_{\tau }T)-\mathbb{Q}(0))\\ & -\frac{1}{{\overset{ˇ}{A}}_{2}}(\tilde{\mathbb{Q}}(r_{\tau }T)-\tilde{\mathbb{Q}}(0))-\frac{1}{{\overset{ˇ}{A}}_{3}}(\overset{―}{\mathbb{Q}}(r_{\tau }T)-\overset{―}{\mathbb{Q}}(0)), \end{array}$$

and


$$\begin{array}{c} \mathrm{Bias}(\tau )=\frac{1}{{\overset{ˇ}{A}}_{1}}\mathrm{Co}{\left(\frac{1}{r_{\tau }T}\sum_{s=1}^{r_{\tau }T}X_{s}^{\prime }X_{s}\right)}^{\;-1}\left(\frac{1}{r_{\tau }T}\sum_{s=1}^{r_{\tau }T}X_{s}^{\prime }(\hat{ℬ}(\tau =s)-ℬ(\tau =s))\right)=o_{p}(c), \end{array}$$

where


$$\begin{array}{c} \mathrm{Co}=(U^{{𝒯}_{2}}E(\hat{y})-U^{{𝒯}_{1}}E(\hat{y}),\;0,\;{\overset{ˇ}{A}}_{4},\;0),\;c=\frac{(t_{S}+\tau -1{)}^{H}}{\left(t_{S}+\tau \right)},\;r_{\tau }=\frac{t_{S}+\tau }{T}, \end{array}$$

H∈(0,1) is the Hurst parameter,


$$\begin{array}{cc} \hat{ℬ}(\tau =1) & =(\underset{t_{S}}{\underbrace{0,0,\ldots ,0}},0{)}^{\prime },\\ \hat{ℬ}(\tau =2) & =(\underset{t_{S}}{\underbrace{0,0,\ldots ,0}},\hat{\beta }(\tau =1),0{)}^{\prime },\\ \hat{ℬ}(\tau =3) & =(\underset{t_{S}}{\underbrace{0,0,\ldots ,0}},\hat{\beta }(\tau =1),\hat{\beta }(\tau =2),0{)}^{\prime },\\ & \;\vdots \\ \hat{ℬ}(\tau =T-t_{S}+1) & =(\underset{t_{S}}{\underbrace{0,0,\ldots ,0}},\hat{\beta }(\tau =1),\hat{\beta }(\tau =2),\ldots ,\hat{\beta }(\tau =T-t_{S}){)}^{\prime }, \end{array}$$

and


$$\begin{array}{cc} {\overset{ˇ}{A}}_{1} & =(U^{{𝒯}_{2}}E(\hat{y})-U^{{𝒯}_{1}}E(\hat{y}))\frac{U^{𝒯}E(S\hat{y})-U^{𝒯}E(S)U^{𝒯}E(\hat{y})}{U^{𝒯}E(\hat{y}\hat{y})-U^{𝒯}E(\hat{y})U^{𝒯}E(\hat{y})},\\ {\overset{ˇ}{A}}_{2} & =\frac{1}{U^{𝒯}E(S\hat{y})-U^{𝒯}E(S)U^{𝒯}E(\hat{y})},\\ {\overset{ˇ}{A}}_{4} & =(U^{{𝒯}_{2}}E(\hat{y})-U^{{𝒯}_{1}}E(\hat{y}))\frac{U^{𝒯}E(Z\hat{y})-U^{𝒯}E(Z)U^{𝒯}E(\hat{y})}{U^{𝒯}E(\hat{y}\hat{y})-U^{𝒯}E(\hat{y})U^{𝒯}E(\hat{y})},\\ {\overset{ˇ}{A}}_{3} & =\frac{U^{𝒯}E(\hat{y})}{U^{𝒯}E(S\hat{y})-U^{𝒯}E(S)U^{𝒯}E(\hat{y})}. \end{array}$$

for ${𝒯}_{1}=\{1,2,\ldots ,t_{S}-1\}$, ${𝒯}_{2}=\{t_{S},t_{S}+1,\ldots ,t_{S}+\tau \}$ and $𝒯=\{1,2,\ldots ,t_{S}+\tau \}$. ℚ(τ), $\tilde{\mathbb{Q}}(\tau )$, $\overset{―}{\mathbb{Q}}(\tau )$ are Gaussian processes with covariance


$$\begin{array}{c} E(\mathbb{Q}(\tau_{1})\mathbb{Q}(\tau_{2}{)}^{\prime })=\int_{0}^{\min(\tau_{1},\tau_{2})}{ℳ}_{𝒳\pi }(s)\;ds,\;E(\tilde{\mathbb{Q}}(\tau_{1})\tilde{\mathbb{Q}}(\tau_{2}{)}^{\prime })=\int_{0}^{\min(\tau_{1},\tau_{2})}{ℳ}_{y^{\prime }\pi }(s)\;ds, \end{array}$$

and


$$\begin{array}{c} E(\overset{―}{\mathbb{Q}}(\tau_{1})\overset{―}{\mathbb{Q}}(\tau_{2}{)}^{\prime })=\int_{0}^{\min(\tau_{1},\tau_{2})}{ℳ}_{\pi }(s)\;ds. \end{array}$$

Theorem 5 states that the asymptotic distribution of the step-wise estimator is a fractional Brownian motion (fBm) process; when *H* = 0.5 the asymptotic distribution turns out to be the usual Brownian motion. Inference for the step-wise estimator is as the same as the proposed bootstrap procedure.

### Panel settings with staggered treatments adoptions and two-way heterogeneities

We now extend model (19) to allow for two-way heterogeneities. Consider the setting with *N* units and each unit *i* receives one (several) policy(s) or treatment(s), the start times and end times of the treatments adoptions may, but need not, vary by unit. For the balanced staggered treatments adoptions, the whole time interval [1,2,…,T] can be divided into ℙ sub-intervals: $\left[t_{s=1},t_{s=1}+1,\ldots ,t_{s=2}-1\right]$, $\left[t_{s=2},t_{s=2}+1,\ldots ,t_{s=3}-1\right]$, $\left[t_{s=3},t_{s=3}+1,\ldots ,t_{s=4}-1\right]$, ..., $\left[t_{s=\mathbb{P}-1},t_{s=\mathbb{P}-1}+1,\ldots ,t_{s=\mathbb{P}}-1\right]$, $\left[t_{s=\mathbb{P}},t_{s=\mathbb{P}}+1,\ldots ,T\right]$ where s=1,2,…,ℙ denotes the *s*-th sub-interval, *t*_*s*_ is the initial time of the *s*-th sub-interval; and $t_{s+1}-t_{s}=\ell_{s}$, $\ell_{s}$ is the length of the *s*-th sub-interval.

We assume that, on the *s*-th sub-interval, each unit *i* receives a treatment $d_{i,s}=(d_{i,t=t_{s}},d_{i,t=t_{s+1}},\ldots ,d_{i,t=t_{s+1}-1})$, where $d_{i,t=t_{s}}=\ldots =d_{i,t=t_{s+1}-1}\in \{0,1\}$. Then $d_{*,s}=(d_{i=1,s},d_{i=2,s},\ldots ,d_{i=N,s}{)}_{N\times \ell_{s}}^{\prime }$ denotes the treatments indicator for all units on the *s*-th sub-interval. Correspondingly, $\zeta_{*,s}=(\zeta_{i=1,s},\zeta_{i=2,s},\ldots ,\zeta_{i=N,s}{)}_{N\times \ell_{s}}^{\prime }$ denotes the two-way heterogeneous treatments effects for all units on the *s*-th sub-interval with $\zeta_{i,s}=(\zeta_{i,t=t_{s}},\zeta_{i,t=t_{s+1}},\ldots ,\zeta_{i,t=t_{s+1}-1})$, $\zeta_{i,t}$ represents the treatment effect for unit *i* at time *t*. A graphical and intuitive illustration of these definitions is given in the Online Supplementary Material S3 File.

**Definition 6 (Phase states and phase transitions).** *The sub-intervals* s=1,2,…,ℙ *are called phase states, the switch from phase state s to s + 1 is called a phase transition. A phase state corresponds to a treatment state, the switch from one treatment state* $d_{i,s}$ *to another* $d_{i,s+1}$ *for the same unit i is called a heterogeneous phase transition if and only if* $d_{i,s}\neq d_{i,s+1}$*, otherwise it is a homogeneous phase transition.*

The DGP of panel settings with staggered treatments adoptions we consider now is


$$\begin{array}{c} Y_{*,*}\equiv \overset{\mathbb{P}}{\underset{s=1}{⨁}}\{{𝔻}_{*,s}\circ \zeta_{*,s}+X_{*,s}\circ \alpha_{*,s}+Z_{*,s}\circ \nu_{*,s}\}+\mu_{*,*}+\nu_{*,*}+\epsilon_{*,*}, \end{array}$$ (22)

where ⨁ denotes the horizontal stacking of matrices, i.e., $C_{N,(T_{1}+T_{2})}=A_{N,T_{1}}⨁B_{N,T_{2}}$ representing the stacking of matrices *A* and *B*, ∘ denotes the Hadamard product, $Y_{*,*}\equiv (Y_{*,s=1},Y_{*,s=2},\ldots ,Y_{*,s=\mathbb{P}}{)}_{N\times T}$ is the outcomes of all units on the whole time interval, $Y_{*,s}=(Y_{i=1,s},Y_{i=2,s},\ldots ,Y_{i=N,s}{)}_{N\times \ell_{s}}^{\prime }$ is the outcomes of all units on the *s*-th sub-interval, and $Y_{i,s}=(Y_{i,t=t_{s}},Y_{i,t=t_{s}+1},\ldots ,Y_{i,t=t_{s+1}-1}{)}_{1\times \ell_{s}}$ is the outcome of unit *i* on the *s*-th sub-interval, $T=\sum_{s}\ell_{s}$ is the total length of the sub-intervals; $X_{*,s}=(X_{i=1,s},X_{i=2,s},\ldots ,X_{i=N,s}{)}_{N\times \ell_{s}}^{\prime }$ are unobservable (or omitted) confounders driving ${𝔻}_{*,s}$ and $Y_{*,s}$ for s=1,2,…,ℙ with $X_{i,s}=(X_{i,t=t_{s}},X_{i,t=t_{s}+1},\ldots ,X_{i,t=t_{s+1}-1}{)}_{1\times \ell_{s}}$ representing the confounder for unit *i* on the *s*-th sub-interval, the coefficients are $\alpha_{*,s}=(\alpha_{i=1,s},\alpha_{i=2,s},\ldots ,\alpha_{i=N,s}{)}_{N\times \ell_{s}}^{\prime }$ with $\alpha_{i,s}=(\alpha_{i},\alpha_{i},\ldots ,\alpha_{i}{)}_{1\times \ell_{s}}$; $Z_{*,s}$ are observed exogenous control variables, which is similarly structured as $X_{*,s}$, the structure of $\nu_{*,s}$ is similar to $\alpha_{*,s}$; $\mu_{*,s}$ is unit fixed effect and $\nu_{*,s}$ is time fixed effect. We assume that: $\mathrm{cov}({𝔻}_{*,s=j},\epsilon_{i,t})\neq 0$, and $\mathrm{cov}(\epsilon_{i,t+n},\epsilon_{i,t})\sim H(2H-1)n^{-2(1-H)}$ as n→∞, H∈(0,1) is the Hurst parameter, $i^{\prime }\neq i\in \{1,2,\ldots ,N\}$, t=1,2,…,T. Note that Model (1) is a special case of model (22) with ℙ=3. The parameter of interest are the two-way heterogeneous treatments effects $\zeta_{*,s}$ on every phase state s=1,2,…,ℙ.

The non-matrix form of (22) with forward point-wise step *h* is:


$$\begin{array}{c} Y_{i,t}-{\tilde{\zeta }}_{i,t}^{-}\cdot 𝕀(t\leq t_{i,k})-{\tilde{\zeta }}_{i,t}^{+}\cdot 𝕀(t\geq t_{i,k}+h)=d_{i,t}\zeta_{i,t}+X_{i,t}\alpha_{i,t}+Z_{i,t}\gamma_{i,t}+\mu_{i}+\nu_{t}+\epsilon_{i,t},\\ t=1,2,\ldots ,t_{i,k}-1+h\;\text{for }h=1,2,\ldots ,T-t_{i,k}, \end{array}$$ (23)

$t_{i,k}$ is the first time unit *i* receives treatment, k∈{2,3,…,ℙ}; ${\tilde{\zeta }}_{t}^{-}=0$ and ${\tilde{\zeta }}_{t}^{+}\equiv {\hat{\zeta }}_{t-h}$. For a given *h*, consider the step-wise first-order difference of model (23):


$$\begin{array}{c} \Delta Y_{i,t}-{\tilde{\zeta }}_{i,t}^{-}\cdot 𝕀(t\leq t_{i,k})-{\tilde{\zeta }}_{i,t}^{+}\cdot 𝕀(t\geq t_{i,k}+h)=\Delta d_{i,t}\zeta_{i,t}+\Delta X_{i,t}\alpha_{i,t}+\Delta Z_{i,t}\gamma_{i,t}+\Delta \nu_{t}+\Delta \epsilon_{i,t}, \end{array}$$

where $\Delta \sigma_{i,t}=\sigma_{i,t}-\frac{1}{t_{i,k}+h}\sum_{t=1}^{t_{i,k}+h}\sigma_{i,t}$, $\sigma_{i,t}\in \{y_{i,t},d_{i,t},X_{i,t},\nu_{t},\epsilon_{i,t}\}$. Suppose that $\sum_{t=1}^{t_{i,k}+h}d_{i,t}<\infty$ for all *i,* as $t_{i,k}+h\to \infty$ we will get


$$\begin{array}{c} \tilde{\Delta Y_{i,t}}=d_{i,t}\zeta_{i,t}+\Delta X_{i,t}\alpha_{i,t}+\Delta Z_{i,t}\gamma_{i,t}+\Delta \nu_{t}+\Delta \epsilon_{i,t}+o_{p}(1) \end{array}$$ (24)

with $\tilde{\Delta Y_{i,t}}=\Delta Y_{i,t}-{\tilde{\zeta }}_{1,t}^{-}\cdot 𝕀(t\leq t_{i,k})-{\tilde{\zeta }}_{i,t}^{+}\cdot 𝕀(t\geq t_{i,k}+h)$.

**Assumption 7** *For the Proximal Variables* $\psi_{i,t}$*: (i) the random variables* $\Delta X_{i,t}$ *and* $\Delta \psi_{i,t}$ *are of high correlations,* $\Delta \nu_{t}$ *and* $\Delta \psi_{i,t}$ *are also of high correlation; (ii)* $\Delta \psi_{i,t}$ *is independent of* $\Delta \epsilon_{i,t}$*; (iii) there exists a continuous function* $g\in {𝒞}^{p}$, *p* ≥ 1*, such that:*


$$\begin{array}{c} \Delta X_{i,t}=\tilde{a}\;g(\tilde{\Delta }y^{\prime }(i,t))+\tilde{b}+{\tilde{u}}_{i,t},\;\Delta \nu_{t}=\tilde{c}\;g(\tilde{\Delta }y^{\prime }(i,t))+\tilde{d}+{\tilde{w}}_{i,t}, \end{array}$$

*with* $\tilde{\Delta }y^{\prime }(i,t)=y^{\prime }(i,t)-\frac{1}{t_{i,k}+h}\sum_{t=1}^{t_{i,k}+h}y^{\prime }(i,t)$, $y^{\prime }(i,t)$ *is defined similarly to model (7),* $\tilde{a},\tilde{b},\tilde{c},\tilde{d}\in \mathbb{R}⧵\{0\}$.

In line with models (7), (8), (11) and (17), model (24) are then turned into varying coefficients panel forms. Estimate this model by varying coefficient fixed effects methods (see, e.g., [76–78]), and by the estimation Steps 3.1–3.2, Steps 4.1–4.3 and Assumption 7, we will get consistent estimators ${\hat{\zeta }}_{i,t}$ respectively for i=1,2,…,N and t=1,2,…,T by applying Theorem 2 and Theorem 5. The bootstrap inference method proposed can be herein applied. See the Online Supplementary Material S3 File for details.

## Numerical examples

### Monte Carlo simulations

**Data Generating Process.** To evaluate the performances of our method, we generate potential outcomes from a varying coefficients linear model with staggered treatments:


$$\begin{array}{c} y_{it}=W_{it}\eta_{it}+D_{it}\xi_{it}+Z_{it}\gamma_{it}+U_{it},\;t=1,2,\ldots ,T,\;i=1,2,\ldots ,N, \end{array}$$ (25)

where $D_{it}=[P_{i}]$, $X_{it}=[P_{i,1}]$, $W_{it}=[P_{i,2}]+W_{t\Delta }^{H}$, $Z_{it}=[P_{i,3}]+W_{t\Delta }^{H}$, $U_{it}=X_{it}\alpha_{it}+e_{it}+\mu_{i}+\nu_{t}$ and $P_{i,1}\sim N(0,\sigma_{P,i}^{2})$, $P_{i}=I\{P_{i,1}\geq q_{\tau }(P_{i,1})\}$, $P_{i,2}\sim U(a_{i},b_{i})$, $P_{i,3}\sim U(c_{i},d_{i})$, $\alpha_{it}\sim U(e_{i},f_{i})$, $e_{it}\sim N(0,\sigma_{e,i}^{2})$ where *U* denotes the uniform distribution, $q_{\tau }(\cdot )$ denotes the τ-th quantile; $\eta_{it}=10.25+i$, $\gamma_{it}=0.1\cdot i$, $\xi_{it}\sim U(g_{i},h_{i})$. [*A*] denotes the order operator of the vector *A*, i.e., $\left[A]=(A_{\left[1\right]},A_{\left[2\right]},\ldots ,A_{\left[n\right]}\right)$ where $A_{\left[1\right]}\leq A_{\left[2\right]}\leq \ldots \leq A_{\left[n\right]}$ for $A\in \{P_{i},P_{i,1},P_{i,2},P_{i,3}\}$; $W_{t\Delta }^{H}$ is a discrete-time fractional Brownian motion (fBm) with the Hurst parameter *H*:


$$\begin{array}{c} W_{t\Delta }^{H}=\sigma^{-\kappa \Delta }W_{(t-1)\Delta }^{H}+(1-\sigma^{-\kappa \Delta })\mu +\epsilon_{t},\;\epsilon_{t}=\rho \int_{(i-1)\Delta }^{i\Delta }\sigma^{-\kappa (i\Delta -s)}\;d{\bar{B}}_{s}^{H}, \end{array}$$

Δ=L/T denotes the sampling interval, $\kappa \in {\mathbb{R}}^{+}$, $\rho \in {\mathbb{R}}^{+}$ and μ∈ℝ are given parameters, ${\bar{B}}_{t}^{H}$ denotes a zero-mean Gaussian process with the covariance function $\mathrm{cov}({\bar{B}}_{t}^{H},{\bar{B}}_{s}^{H})=\frac{1}{2}(|t{|}^{2H}+|s{|}^{2H}-|t-s{|}^{2H})$, t,s≥0 [79]. $W_{t\Delta }^{H}$ is the discrete-time representation of the continuous-time fractional Ornstein-Uhlenbeck process $dW_{t}=\kappa (\mu -W_{t})dt+\rho d{\bar{B}}_{t}^{H}$ [80].

Model (25) is endogeneous because *X*_*it*_ is correlated with *D*_*it*_, and we assume that *U*_*it*_ is unobservable. We take $\psi_{t}=t$ by Remark 1. The method based on fast Fourier transformation (FFT) to generate $W_{t,\Delta }^{H}$ is provided in [81] and [79]. In this paper we consider *H* = 0.700, κ=0.237, μ=2.417, ρ=0.700, *T* = 500, *N* = 5 and $\Delta =1/2^{8}$, hence *L* = 1.953125. We set $\sigma_{P,i}^{2}=0.02+i\cdot 0.02$, *a*_*i*_ = 0, *b*_*i*_ = 1, $c_{i}=-0.01\cdot i$, $d_{i}=0.01\cdot i$, *e*_*i*_ = 14, *f*_*i*_ = 16, *g*_*i*_ = −*i*, *h*_*i*_ = *i*, τ=0.5 and $\sigma_{e,i}^{2}=0.01$ for model (25). The estimands we are interested in are


$$\begin{array}{c} \theta_{ATT,1}=\frac{1}{NT_{D}}\sum_{i=1}^{N}\sum_{t=t_{i,d}}^{T}\xi_{1,it},\;\theta_{ATT,2}=\frac{1}{N}\sum_{j=1}^{N}(\frac{1}{T-t_{i,d}}\sum_{t=t_{i,d}}^{T}\xi_{2,it}), \end{array}$$

where $\xi_{1,it}$ is the panel model estimand; $\xi_{2,it}$ is the mean group estimand which averages coefficients obtained from separate time-series regressions for each individual; $t_{i,d}$ is the time unit *i* receives treatment, $T_{D}=\sum_{i=1}^{N}(T-t_{i,d})$.

We also compare our method with the literature when untreated units are available. We generate 10 untreated units for each treated unit through model (25), i.e., the *j*-th untreated outcome for the treated unit *i* is generated as follows:


$$\begin{array}{c} Y_{co,it,j}=W_{it}\eta_{it}+Z_{it}\gamma_{it}+U_{it}+U(-0.05j,0.05j),\;t=1,2,\ldots ,T,\;j=1,2,\ldots ,10, \end{array}$$

which guarantees that $Y_{it}-Y_{co,it,j}$ is a martingale process for every *i* and *j* over the time interval {t=1,…,T}.

**Estimation Performances.** The simulation results in terms of bias and mean squared error (MSE) are shown in Table 2. It would be interesting to see that the treatment effects could be consistently estimated by our methods. The biases of the proposed method $\left({\hat{\theta }}_{ATT,1},{\hat{\theta }}_{ATT,2}\right)$ are generally the smallest among all competitors and the biases are decreasing with sample size. The other estimators do not perform well due to the heterogeneities or endogeneities.

**Table 2:** Simulation results with long range dependency (*H* = 0.70).

Estimators	*T* = 100,*N* = 5		*T* = 300,*N* = 5		*T* = 500,*N* = 5	
	Av. Bias	MSE	Av. Bias	MSE	Av. Bias	MSE
without untreated units						
${\hat{\theta }}_{\text{ATT,1}}$	0.428	0.211	0.291	0.094	0.142	0.021
${\hat{\theta }}_{\text{ATT,2}}$	0.585	0.429	0.361	0.165	0.140	0.018
TWFE	1.874	3.838	0.914	1.306	0.843	0.710
did_staggered (or)	0.552	0.341	0.494	0.260	0.598	0.371
did_staggered (dr)	0.552	0.341	0.494	0.260	0.598	0.371
did_staggered (ipw)	0.552	0.341	0.494	0.260	0.598	0.371
before_after	15.020	22.632	13.020	16.423	5.506	30.35
with untreated units						
synthdid	4.993	25.197	4.326	18.991	0.458	0.210
gsynth	4.787	22.929	4.131	17.073	0.458	0.210
did_staggered (or)	0.952	0.955	0.194	0.042	0.225	0.054
did_staggered (dr)	0.952	0.955	0.194	0.042	0.225	0.054
did_staggered (ipw)	0.952	0.955	0.194	0.042	0.225	0.054
did_staggered (sunab)	1.738	3.089	0.977	0.959	0.458	0.210

*Notes:* based on 1000 simulations; “did_staggered (or)”, “did_staggered (ipw)” and “did_staggered (dr)” represents for the outcome regression estimator, inverse probability weighting estimator and double robust estimator proposed in [18,82]; “synthdid” is the synthetic DiD estimator proposed in [83]; “gsynth” is the generalized synthetic control method proposed by [84], and “sunab” is the estimator proposed by. Before_after is the estimator simply compares the means of the treated outcomes before and after the treatments.

We now examine the performances of the confidence interval (CI) for the proposed methods. It can be seen from Table 3 that the coverage probabilities (CP) are increasing with sample size, and generally perform good.

**Table 3:** Empirical coverage probability of the 95% CI for the treatment effect.

Estimator	*T* = 100	*T* = 300	*T* = 500
*N* = 1			
$\theta_{ATT,1}$ (CP)	0.912	0.931	0.950
*N* = 5			
$\theta_{ATT,1}$ (CP)	0.848	0.951	0.856
$\theta_{ATT,2}$ (CP)	0.842	0.962	0.957

Notes: based on 1000 simulations. The coverage probability is estimated by $CP_{CI}=\frac{1}{NT_{D}}\sum_{i=1}^{N}\sum_{t=t_{i,d}}^{T}{\hat{\sigma }}_{It}$, where ${\hat{\sigma }}_{It}=𝕀\{{\hat{\xi }}_{It}\leq \xi_{It}\leq {\hat{\xi }}_{It}^{*}\}$, $\left[{\hat{\xi }}_{It}^{*},{\hat{\xi }}_{It}^{*}\right]$ is the estimated 95% confidence interval for the treatment effect $\xi_{It}$.

**Weak and Invalid CP.** To illustrate the consequences if the chosen proximal variable is weak or invalid, we carried out additional Monte Carlo simulations studies. (i) Weak CP is generated by $\psi_{t,\text{weak}}=\psi_{t}+N(0,\sigma )$, i.e., we introduce different levels of noise σ into the CP variable and examine the resulting effects on the estimation outcomes. (ii) Invalid CP is generated by $\psi_{t,\text{invalid}}=\psi_{t}+\rho e_{1t}+\rho e_{2t}+\ldots +\rho e_{Nt}$, i.e., we introduce different levels of invalidity ρ into the CP variable and examine the resulting effects on the estimation outcomes. The results are shown in the following Tables 4–8.

**Table 4:** Simulation studies when the CP is weak or invalid (*T* = 30, *N* = 5; *CP* = *t*).

Weak CP	σ=15	σ=45	σ=150	σ=550
$\theta_{ATT,1}$	0.436	0.451	0.461	0.467
$\theta_{ATT,2}$	0.867	2.380	1.509	1.532

**Table 5:** Simulation studies when the CP is weak or invalid (*T* = 50, *N* = 5; *CP* = *CF* treated).

Weak CP	σ=15	σ=45	σ=150	σ=550
$\theta_{ATT,1}$	1.707	1.837	1.787	1.824
$\theta_{ATT,2}$	1.658	2.042	1.690	1.825

**Table 6:** Simulation studies when the CP is weak or invalid (*T* = 50, *N* = 5; *CP* = *CF* untreated).

Weak CP	σ=15	σ=45	σ=150	σ=550
$\theta_{ATT,1}$	1.782	1.764	1.712	1.782
$\theta_{ATT,2}$	2.242	1.807	1.681	1.810

**Table 7:** Simulation studies when the CP is weak or invalid (*T* = 50, *N* = 5; *CP* = *CF* treated).

Invalid CP	ρ=1	ρ=50	ρ=100	ρ=500
$\theta_{ATT,1}$	0.375	0.448	0.454	0.456
$\theta_{ATT,2}$	0.645	0.576	0.564	0.547

**Table 8:** Simulation studies when the CP is weak or invalid (*T* = 50, *N* = 5; *CP* = *CF* untreated).

Invalid CP	ρ=1	ρ=50	ρ=100	ρ=500
$\theta_{ATT,1}$	1.690	0.816	0.838	0.902
$\theta_{ATT,2}$	1.639	0.798	0.824	0.901

Interestingly, we find that the bias in estimating the treatment effect is increasing in the invalidity of the CP variable, but not in its weakness. That is, our proposed methods remains largely unbiased even when the CP variable is weak (as shown in Table 4). To understand this phenomenon, a theoretical analysis proving that our estimators are robust to weak CP variable is provided in the Online Supplementary Material S5 File.

**Short panels, and simple serial correlation.** In the paper, we employ the Change-of-Frequency (COF) estimator to estimate the Hurst parameter *H*. This estimator is based on the second differences of the bootstrap sequence, and the consistent convergence of the Hurst parameter does not depend on *T*, but rather on *B*. In practice, the Hurst parameter is only used to construct the normalization factor ${\hat{d}}_{B}$. As long as the bootstrap sample size *B* is sufficiently large, the confidence intervals computed from short time sequences can still maintain the nominal coverage level. To see this, we have conducted simulation studies specifically designed for short time sequences.

**Fig 6:**
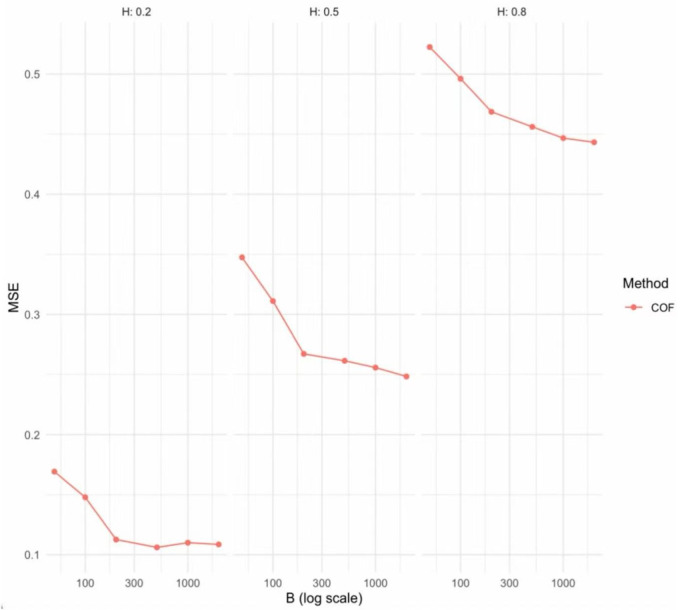
Simulation studies for the COF estimator. The figure shows bias of Hurst estimator vs. bootstrap replications with different level of long range dependency.

From Table 9, we find that even for short panel data, the treatment effect estimator remains consistent. At the same time, we observe from Fig 6 that the bias of the Hurst parameter estimator decreases as the number of bootstrap replications increases, indicating that the proposed COF estimator is feasible. Hence, the estimators perform well even if the time series is short.

**Table 9:** Simulation study for short panels.

*T*,*N*	Estimator	Avg Bias	MSE	CP (95%)
*T* = 10,*N* = 5	$\theta_{ATT,1}$	1.630	2.576	0.548
	$\theta_{ATT,2}$	0.864	1.098	0.693
*T* = 20,*N* = 5	$\theta_{ATT,1}$	0.928	1.315	0.643
	$\theta_{ATT,2}$	0.943	1.268	0.814
*T* = 30,*N* = 5	$\theta_{ATT,1}$	0.478	0.149	0.815
	$\theta_{ATT,2}$	0.515	0.220	0.753
*T* = 50,*N* = 5	$\theta_{ATT,1}$	0.436	0.178	0.920
	$\theta_{ATT,2}$	0.647	0.288	0.910

Apart from these, we carried out additional simulation studies to illustrate our methods’ performances with simple serial correlation, i.e., the model’s error term $\epsilon_{it}$ is generated by an AR(1) model with auto-correlation coefficient ρ.

The robustness of the proposed bootstrap inference framework to both short and long memory shown in Table 10 stems from its sub-interval resampling design, which non-parametrically captures the underlying dependence structure without imposing parametric assumptions. For short memory (e.g., AR(1) with exponentially decaying auto-correlations), the randomly partitioned sub-intervals *I*_*b*_ are asymptotically independent as the sample size *T* grows. Consequently, the bootstrap sequence $\tilde{B}=({\tilde{\beta }}_{1},\ldots ,{\tilde{\beta }}_{B})$ becomes approximately *i.i.d.*, rendering conventional inference valid. For long-range dependence (LRD) where auto-correlations decay hyperbolically, the bootstrap sequence itself retains the long memory property, with its covariance structure satisfying $\mathrm{Cov}({\tilde{\beta }}_{b},{\tilde{\beta }}_{b^{\prime }})\sim (b^{\prime }-b{)}^{-H}ℒ(b^{\prime }-b)$. Rather than ignoring this dependence, the proposed method directly characterizes the limiting distribution of the partial sum ${PS}_{B}$. As established in Theorem 4, after appropriate normalization, the statistic ${ℍ}_{B}$ converges in distribution to a standard normal with the Hermite rank *q* = 1. This unified framework automatically adapts to the dependence strength: under short memory, the Hurst parameter *H* plays no distorting role, and under long memory, it is explicitly incorporated into the normalization factor *d*_*B*_, ensuring valid coverage regardless of the true memory process.

**Table 10:** Simulation studies with simple serial correlation.

	Estimator	Avg Bias	MSE	CP (95%)
ρ=0.1	$\theta_{ATT,1}$	0.947	1.304	0.782
	$\theta_{ATT,2}$	1.212	1.654	0.843
ρ=0.3	$\theta_{ATT,1}$	0.875	1.302	0.814
	$\theta_{ATT,2}$	0.768	1.266	0.904
ρ=0.5	$\theta_{ATT,1}$	0.938	1.276	0.788
	$\theta_{ATT,2}$	0.765	1.031	0.811

### Unilateral divorce law reforms and divorce growth

We illustrate our proposed methods by analyzing the heterogeneous treatment effects of no-fault divorce law reforms on divorce growth in China. Unilateral (or no-fault) divorce reform carried out worldwide allows either spouse to end a marriage, redistributing property rights and bargaining power relative to fault-based divorce regimes [85,86][85] . The Marriage Law of the People’s Republic of China (promulgated in 2003 and implemented in 2004) stipulates that the condition for divorce is emotional breakdown rather than one party’s fault, so both spouses can file a divorce lawsuit even if both spouses are not at fault (Before *The Marriage Law of the People’s Republic of China (2004)*, the condition for one spouse to file a divorce lawsuit in China is that the other party is at fault, i.e.,bigamy, cohabitation with others, domestic violence, maltreat or abandon. Many divorce lawsuits cannot be supported by the court because one spouse cannot provide sufficient evidence to prove the other’s fault. For more details on Chinese divorce system). What’s special for China is that the implement of the law is “one size fits all”, which means that all 34 provincial districts across the country are all treated units. Hence we are unable to collect untreated units for policy evaluation. The method proposed in this paper is therefore most suitable.

All 30 provinces’ registered divorce data per year from 1990 to 2010 are collected from the National Bureau of Statistics (The data of Chongqing etc. are not provided by the official statistics. Please see the website of the National Bureau of Statistics for reasons: https://data.stats.gov.cn/english/easyquery.htm?cn=E0103). The divorces all show an upward growth trend, hence we take $\psi_{it}=t$ for all *i*; we take *Z*_*it*_ to be the GDP per capita, because GDP per capita is found to be highly correlated with factors driving the growth of divorce [85]. *D*_*it*_ takes value 1 after the year 2003 and 0 otherwise.

**Fig 7:**
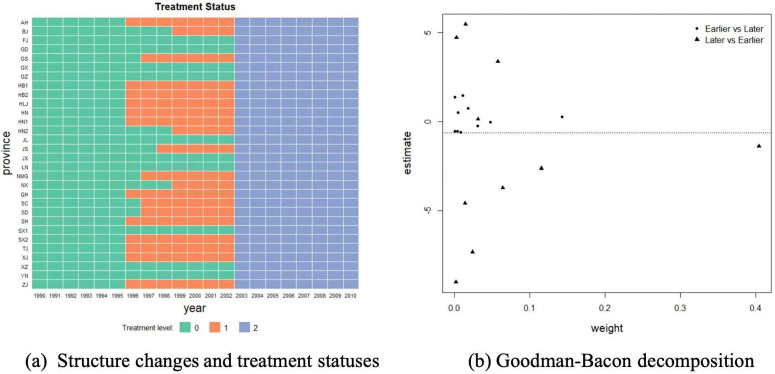
Structure changes and treatments statuses (left), and Goodman-Bacon decomposition (right). Treatment level 1 indicates the periods when structure changes commence and persist, level 2 indicates the periods when treatment starts and continues, as well as the periods when structure changes last after level 1.

As shown in Definition 5 and Theorem 1, if there exist structural change *S*_*it*_ in divorce before the implementation of the law *D*_*it*_, treatment effect could be contaminated with structure change effect. Hence, structure change effect should be removed when evaluating the law enforcement effect. *S*_*it*_ is then detected by the Chow test through the R package “sctest” using data ranging from 1990 to 2002. The structure changes and treatments statuses are shown in Fig 7(a). Fig 7(b) further shows that staggering effect exist in structure changes [16].

The parameters we are interested in are the average treatment effect on the treated, the average structure change effect and the total average effect:


$$\begin{array}{c} \theta_{ATT}=(1/|N_{tr}|T_{D})\cdot \sum_{i\in N_{tr}}\sum_{t\in T_{D}}\xi_{it}, \end{array}$$

$$\begin{array}{c} \theta_{ASC}=(1/|N_{tr}|T_{S})\cdot \sum_{i\in N_{tr}}\sum_{t\in T_{S}}\beta_{it}, \end{array}$$

$$\begin{array}{c} \theta_{TAE}=(1/|N_{tr}|T_{S0})\cdot \sum_{i\in N_{tr}}\sum_{t\in T_{S}}(\beta_{it}+\xi_{it}), \end{array}$$

in which $N_{tr}=\{1,2,\ldots ,30\}$, $T_{D}=\{2003,2004,\ldots ,2010\}$, $T_{S}={⋃}_{i\in N_{tr}}\{T_{i,S},T_{i,S}+1,\ldots ,2010\}$, and $T_{i,S}\in \{1996,1997,1998,1999\}$ is the start time of the structure change for province *i* as shown in Fig 7(a). The estimation results are collected in Table 11.

**Table 11:** Unilateral divorce law reform effect on divorce growth.

	new_1		new_2		TWFE		Pooling_ols	
$\theta_{ASC}$	−3.324***	−1.131	−2.886***	−1.036***	−2.332***	−5.896***	0.706	−1.079
	(0.008)	(2.290)	(0.049)	(0.003)	(0.509)	(0.586)	(0.789)	(0.782)
$\theta_{ATT}$	4.478***	2.740***	4.679***	2.677***	2.163***	6.029***	2.788***	5.336***
	(0.010)	(0.002)	(0.087)	(0.002)	(0.366)	(0.391)	(0.609)	(0.582)
$\theta_{TAE}$	−0.681***	0.643	−0.134***	0.632***	−0.169	0.133	3.494***	4.257***
CP variable	✓	✓	✓	✓	×	×	×	×
Control variable	✓	×	✓	×	✓	×	✓	×
year fixed effects	✓	✓	✓	✓	✓	✓	×	×
province fixed effects	✓	✓	✓	✓	✓	✓	×	×

Notes: Standard errors in parentheses. *** *p* < 0.01, ** *p* < 0.05, * *p* < 0.1. “new_1” and “new_2” refer to two different estimand $\theta_{1}$ and $\theta_{2}$ (see text).

It is shown that the structure changes around 1996–1999 lead to a decline of divorce (33240 couples on average), while the implements of unilateral divorce reform since 2003 lead to an increase of divorce (44780 couples on average). TWFE and OLS underestimates the treatment effects and structure change effects for neglecting the heterogeneities and endogeneities caused by two-way variability and potential confounders.

The 1997 Asian financial crisis exerted great negative economic impacts on China, many people lost their jobs or experienced a decrease in asset and income; meanwhile, a major flood occurred across China in 1998, resulting in exceeding 20 billion USD direct economic losses. Under these scenarios, couples will choose to overcome difficulties together, resulting in a decrease in divorce [85]. On the other hand, the unilateral divorce reform lowers the threshold and cost of divorce, making it easier to divorce, hence resulting in an increase of divorce. This finding is in accordance with the literature [86,87].

**Fig 8:**
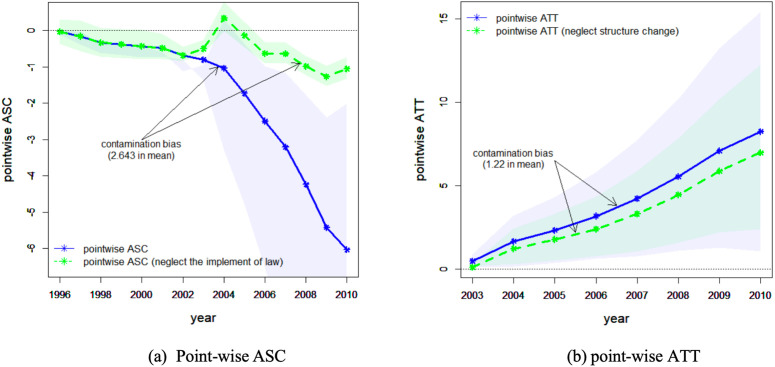
Point-wise estimation of ASC and ATT.

We also report the point-wise estimations of the ASCs and ATTs in Fig 8 (left: ${\text{ASC}}_{t}=(1/|N_{tr}|)\cdot \sum_{i\in N_{tr}}\beta_{it};\text{right:}ATT_{t}=(1/|N_{tr}|)\cdot \sum_{i\in N_{tr}}\xi_{it}$). It can be seen that neglecting the treatment *D*_*it*_ (Fig 8(a)) or structure changes *S*_*it*_ (Fig 8(b)) will lead to contamination bias [20], and the bias will lead to wrong empirical conclusions. This warns us be aware of other potential distributional shifts if our focus is the effect of some specific social policy treatment.

## Conclusion

We propose a novel method to disentangle one treatment effect from another in time-series natural experiment settings with staggered and endogenous treatments adoptions, we allow the social-economic outcome to be long range dependent and no untreated units along with IVs are available. The Conditional Mean Symmetry condition constructed by the proposed Common Proximal Variable enables us to identify the treatments effects under the Neyman-Rubin counterfactual framework, while a two-step point-wise estimation approach allows us to consistently estimate the heterogeneous treatments effects. We show that the asymptotic distribution of the estimator is a fractional Brownian motion process with long range dependency, thereby a bootstrap procedure is considered for inference. We provide a new identification and estimation framework for empirical researchers and decision-makers whose interest lies in evaluating social policies effects.

## Supporting information

S1 File Notation table.
(PDF)

S2 File Main proofs of Proposition and Theorems.
(PDF)

S3 File Detailed estimation procedure for panel model.
(PDF)

S4 File Illustration of panel settings with staggered treatments adoptions.
(PDF)

S5 File Impact of choosing a weak or invalid CP variable on estimation and inference.
(PDF)
