# Supplementary material for: Estimating endogenous treatments effects under long-range dependency without untreated controls
Source: PLoS One. 2026 Jun 3;21(6):e0347847. doi: 10.1371/journal.pone.0347847 (PMC13232959; doi:10.1371/journal.pone.0347847)
Supplement: S1 File — Notation table. (PDF) [file pone.0347847.s001.pdf]

Estimating Endogenous Treatments Effects under Long Range Dependency without Untreated Controls

SUPPLEMENT 1. Notations and Descriptions

Below is a comprehensive table summarizing the main notations used throughout the paper. The notations are organized by category for clarity.

Table 1: Notations and descriptions.

Notation	Description
Variables and Indices	
y_{it}	Observed scalar outcome for unit i at time t, $i = 1, 2, \ldots, N$; $t = 1, 2, \ldots, T$.
S_{it}	Binary treatment indicator for the first treatment (e.g., policy S) for unit i at time t.
D_{it}	Binary treatment indicator for the second treatment (e.g., policy D) for unit i at time t.
W_{it}	Unobservable confounder affecting S_{it} and y_{it}; dimension one.
X_{it}	Unobservable confounder affecting D_{it} and y_{it}; dimension one.
Z_{it}	Vector of exogenous control variables (dimension d_Z), independent of treatments and error term.
μ_i, μ_t	Individual fixed effect, and time fixed effect.
ε_{it}	Idiosyncratic error term, may be correlated with treatments.
λ_{it}	Generic placeholder for an endogenous treatment variable (e.g., S_{it} or D_{it}).
Parameters	
β_{it}	Heterogeneous treatment effect of S_{it} on y_{it}.
ξ_{it}	Heterogeneous treatment effect of D_{it} on y_{it}.
η_{it}	Coefficient on unobservable confounder W_{it}.
α_{it}	Coefficient on unobservable confounder X_{it}.
γ_{it}	Coefficient on exogenous controls Z_{it}.
Treatment timing and intervals	
t_{Si}	Time when treatment S starts for unit i.
t_{Di}	Time when treatment D starts for unit i.
τ	Generic subset of the time index set $\{1, 2, \ldots, T\}$.
τ_1	Pretreatment period for the first treatment: $\{1, \ldots, t_S - 1\}$.
τ_2	Period between the two treatments: $\{t_S, \ldots, t_D - 1\}$.
τ_3	Pretreatment period for the second treatment: $\{1, \ldots, t_D - 1\}$.
τ_4	Period after the second treatment starts: $\{t_D, \ldots, T\}$.
ℓ	Generic subset (subinterval) of time indices.
Potential outcomes	
Continued on next page	

Table 1 – continued from previous page	
**Notation**	**Description**
$y_t(0,0)$	Potential outcome when neither treatment is received.
$y_t(1,0)$	Potential outcome when only treatment S is received.
$y_t(1,1)$	Potential outcome when both treatments are received.
$y_t(0,1)$	Potential outcome when only treatment D is received.
Common proximal variable (CP)	
ψ_t	Common proximal variable satisfying Assumptions 2.2–2.3. Often taken as t in trend stationary scenarios.
$g(\cdot)$	A continuous function (in C^p) linking ψ_t to the confounders; used in DoobMeyer type decompositions.
Bernstein expansions	
$\rho_{y,2}$, $\rho_{y,1}$, $\rho_{\lambda,2}$, $\rho_{\lambda,1}$	Bernstein coefficients in expansions (7)–(8).
$a_{\sigma,b} = b\rho_{\sigma,b}$	Scaled Bernstein coefficients used in estimation.
$\widehat{y}'(t)$	Estimated first derivative of y_t with respect to ψ_t, used in the auxiliary regression.
Operators and expectations	
$E(\cdot)$	Expectation operator.
$U^\ell E(\cdot)$	Subsample expectation operator over the time subset ℓ.
$\mathrm{Cov}(\cdot,\cdot)$	Covariance operator.
$\rho(\cdot,\cdot)$	Correlation coefficient.
$\sigma(\cdot)$	Sigmaalgebra generated by a random variable.
Assumptionrelated notations	
CMS	Conditional Mean Symmetry (Assumption 2.5).
CMI	Conditional Mean Independence (Theorem 3.2).
CP	Common Proximal Variable (Definition 2.2).
fBm	Fractional Brownian motion.
H	Hurst parameter, measuring longrange dependency ($0 < H < 1$).
Estimation step notations	
d_B	Normalizing factor in bootstrap asymptotics.
q	Hermite rank of a function.
$H_k(\cdot)$	k-th Hermite polynomial.
$\mathbb{P}S_B$	Partial sum of bootstrap statistics.
$\mathbb{H}_B$	Studentized version of the bootstrap statistic.
Panel data extensions	
$\mathbb{P}$	Number of phase states (subintervals) in staggered adoption panel.
$s = 1, 2, \ldots, \mathbb{P}$	Index for phase state.
ℓ_s	Length of the s-th phase state.
$d_{i,s}$	Treatment indicator for unit i during phase state s (constant within the phase).
$\zeta_{i,s}$	Heterogeneous treatment effect for unit i during phase state s.
$\bigoplus$	Horizontal stacking of matrices.
$\circ$	Hadamard (elementwise) product.
$Y_{*,*}$, $X_{*,*}$, $Z_{*,*}$	Matrices stacking outcomes and covariates for all units and time periods.
Δ	Firstdifference operator used in panel estimation (e.g., Δy_{it}).
Proof notations	
$\widehat{\Delta y}_{it}$	Firstdifferenced outcome after adjusting for previously estimated effects.
$d'(i,t)$	First derivative of the treatment indicator with respect to ψ_t (unitspecific).
$\widehat{\Delta y}'(i,t)$	Estimated first derivative of the differenced outcome.
$K(\cdot,\cdot)$	A C^2 function capturing the balance condition between derivatives.
E_B, Var_B	Expectation and variance operator with respect to the bootstrap distribution.
$\mathcal{M}_X(t)$, $\mathcal{M}_\pi(t)$	Covariance matrices of stochastic processes in the asymptotic distribution of pointwise estimators.

Continued on next page

Table 1 – continued from previous page	
**Notation**	**Description**
$\mathbb{Q}$, $\widetilde{\mathbb{Q}}$, $\overline{\mathbb{Q}}$	Gaussian processes appearing in the limit distribution of $\widehat{\beta}(\tau)$.
