# Supplementary material for: Estimating endogenous treatments effects under long-range dependency without untreated controls
Source: PLoS One. 2026 Jun 3;21(6):e0347847. doi: 10.1371/journal.pone.0347847 (PMC13232959; doi:10.1371/journal.pone.0347847)
Supplement: S2 File — Main proofs of Proposition and Theorems. (PDF) [file pone.0347847.s002.pdf]

Estimating Endogenous Treatments Effects under Long Range Dependency without Untreated Controls

SUPPLEMENT 2. Proofs of Proposition and Theorems

Lemma S.1 Let A and B be two matrices of the same size (e.g. $m \times n$), under Definition 1, the following statements are equivalent:

(i) $\mathcal{M}^{-1}(A+B) = \mathcal{M}^{-1}(A) + \mathcal{M}^{-1}(B)$;

(ii) $\mathcal{M}^{-1}(A) \subseteq \mathcal{M}^{-1}(A+B)$ or equivalent $\mathcal{M}^{-1}(B) \subseteq \mathcal{M}^{-1}(A+B)$;

(iii) $\text{rank}(A+B) = \text{rank}([A\ B])$, where $[A\ B]$ is the augmented matrix;

(iv) $\text{Ker}((A+B)') \subseteq \text{Ker}(A')$ or equivalent $\text{Ker}((A+B)') \subseteq \text{Ker}(B')$.

Proof of Lemma S.1: We always have the inclusion $\mathcal{M}^{-1}(A+B) \subseteq \mathcal{M}^{-1}(A) + \mathcal{M}^{-1}(B)$. Hence, equality holds if and only if the reverse inclusion $\mathcal{M}^{-1}(A) + \mathcal{M}^{-1}(B) \subseteq \mathcal{M}^{-1}(A+B)$ is true, which is equivalent to both $\mathcal{M}^{-1}(A) \subseteq \mathcal{M}^{-1}(A+B)$ or $\mathcal{M}^{-1}(B) \subseteq \mathcal{M}^{-1}(A+B)$. We now prove that (i)$\Leftrightarrow$(ii)$\Leftrightarrow$(iii)$\Leftrightarrow$(iv).

(i)$\Leftrightarrow$(ii): If $\mathcal{M}^{-1}(A) \subseteq \mathcal{M}^{-1}(A+B)$, then for any column b_j of B, we have $b_j = (a_j + b_j) - a_j$ where a_j is the corresponding column of A. Since $a_j + b_j$ and a_j both lie in $\mathcal{M}^{-1}(A+B)$, their difference b_j also lies in $\mathcal{M}^{-1}(A+B)$. Thus $\mathcal{M}^{-1}(B) \subseteq \mathcal{M}^{-1}(A+B)$. Consequently, $\mathcal{M}^{-1}(A) + \mathcal{M}^{-1}(B) \subseteq \mathcal{M}^{-1}(A+B)$, and together with the trivial inclusion we obtain (i). Conversely, (i) clearly implies $\mathcal{M}^{-1}(A) \subseteq \mathcal{M}^{-1}(A) + \mathcal{M}^{-1}(B) = \mathcal{M}^{-1}(A+B)$. So (i) and (ii) are equivalent.

(ii)$\Leftrightarrow$(iii): Note that $\text{rank}([A\ B]) = \dim(\mathcal{M}^{-1}(A) + \mathcal{M}^{-1}(B))$. From (ii) we have $\mathcal{M}^{-1}(A) + \mathcal{M}^{-1}(B) \subseteq \mathcal{M}^{-1}(A+B)$, and since $\mathcal{M}^{-1}(A+B) \subseteq \mathcal{M}^{-1}(A) + \mathcal{M}^{-1}(B)$ always holds, we get equality of the subspaces, hence their dimensions are equal: $\text{rank}(A+B) = \text{rank}([A\ B])$. Conversely, if the ranks are equal, then the inclusion $\mathcal{M}^{-1}(A+B) \subseteq \mathcal{M}^{-1}(A) + \mathcal{M}^{-1}(B)$ together with equal dimensions forces $\mathcal{M}^{-1}(A+B) = \mathcal{M}^{-1}(A) + \mathcal{M}^{-1}(B)$, which implies (ii).

(iii)$\Leftrightarrow$(iv): For any subspace U, its orthogonal complement is $U' = \{x \mid x'u = 0\}$ for all $u \in U$. It is well known that $U \subseteq V$ if and only if $V' \subseteq U'$. Taking $U = \mathcal{M}^{-1}(A)$ and $V = \mathcal{M}^{-1}(A+B)$, we have $\mathcal{M}^{-1}(A) \subseteq \mathcal{M}^{-1}(A+B)$ if and only if $\mathcal{M}^{-1}(A+B)' \subseteq \mathcal{M}^{-1}(A)'$. But $\mathcal{M}^{-1}(X)' = \text{Ker}(X')$ for any

matrix X. Hence (ii) is equivalent to $\mathrm{Ker}((A+B)') \subseteq \mathrm{Ker}(A')$. By symmetry, the same holds with B in place of A. $\square$

Proof of Proposition 1: For the adjacency matrices $\mathcal{A}, \mathcal{B}, \mathcal{C}, \mathcal{D}$, by the notations of Assumption 1, define $X = (W, S, D, X, Z, y)$ and

$$\mathcal{E} = \begin{pmatrix} 1 & 0 & 0 & 0 & 0 & -1 \\ 0 & 1 & -1 & 0 & 0 & -1 \\ 0 & 0 & 1 & 0 & 0 & -1 \\ -1 & 0 & 0 & 1 & 0 & -1 \\ 0 & 0 & 0 & 0 & 1 & -1 \\ 0 & 0 & 0 & 0 & 0 & 0 \end{pmatrix},$$

$$\mathcal{E}_1 = \begin{pmatrix} 0 & 0 & 0 & 0 & 0 & 0 \\ 0 & 1 & -1 & 0 & 0 & -1 \\ 0 & 0 & 1 & 0 & 0 & -1 \\ 0 & 0 & 0 & 0 & 0 & 0 \\ 0 & 0 & 0 & 0 & 1 & -1 \\ 0 & 0 & 0 & 0 & 0 & 0 \end{pmatrix}, \quad \mathcal{E}_2 = \begin{pmatrix} 1 & 0 & 0 & 0 & 0 & -1 \\ 0 & 0 & 0 & 0 & 0 & 0 \\ 0 & 0 & 0 & 0 & 0 & 0 \\ -1 & 0 & 0 & 1 & 0 & -1 \\ 0 & 0 & 0 & 0 & 0 & 0 \\ 0 & 0 & 0 & 0 & 0 & 0 \end{pmatrix}.$$

Then by Definition 1 and Assumption 1, the parameters $\theta_2 = (\eta_2, \beta_2, \xi_2, \alpha_2, \gamma_2)$ can be identified in model (3) if and only if $y \in \mathcal{M}^{-1}(X(\mathcal{B} + \mathcal{E}))$, the parameters $\theta_3 = (\eta_3, \beta_3, \gamma_3, 0, 0)$ can be identified in model (4) if and only if $y \in \mathcal{M}^{-1}(X(\mathcal{C} + \mathcal{E}_1))$ and the parameters $\theta_4 = (0, 0, \xi_4, \alpha_4, \gamma_4)$ can be identified in model (5) if and only if $y \in \mathcal{M}^{-1}(X(\mathcal{D} + \mathcal{E}_2))$ (Rao & Toutenburg, 1995). By Assumption 1 again, we will get

$$\begin{aligned} y &\in \mathcal{M}^{-1}(X(\mathcal{B} + \mathcal{E})) \\ &\subseteq \mathcal{M}^{-1}(X\mathcal{B}) + \mathcal{M}^{-1}(X\mathcal{E}) \\ &= \mathcal{M}^{-1}(X(\mathcal{C} + \mathcal{D})) + \mathcal{M}^{-1}(X(\mathcal{E}_1 + \mathcal{E}_2)) \\ &\subseteq \mathcal{M}^{-1}(X\mathcal{C}) + \mathcal{M}^{-1}(X\mathcal{D}) + \mathcal{M}^{-1}(X\mathcal{E}_1) + \mathcal{M}^{-1}(X\mathcal{E}_2) \\ &= \mathcal{M}^{-1}(X\mathcal{C} + X\mathcal{E}_1) + \mathcal{M}^{-1}(X\mathcal{D} + X\mathcal{E}_2) \\ &\subseteq \mathcal{M}^{-1}(X(\mathcal{C} + \mathcal{E}_1)) + \mathcal{M}^{-1}(X(\mathcal{D} + \mathcal{E}_2)), \end{aligned}$$

where the fifth equality holds true by Lemma S.1 and Assumption 1. On the contrary, one can verify that $y \in \mathcal{M}^{-1}(X(\mathcal{A} + \mathcal{E}))$ does not belong to $\mathcal{M}^{-1}(X(\mathcal{C} + \mathcal{E}_1)) + \mathcal{M}^{-1}(X(\mathcal{D} + \mathcal{E}_2))$, thereby confirms that the treatments effects can be disentangled from each other under model (3) with the adjacency matrix $\mathcal{B}$ but not under model (2) with the adjacency matrix $\mathcal{A}$. $\square$

Proof of Theorem 1: **(i) the Before-After part.** For model (6) and (10),

by Definition 3 we will get

$$\begin{aligned}
\beta &= \mathrm{E}\left(y(1,0) \mid S=1, D=0, \omega\right) - \mathrm{E}\left(y(0,0) \mid S=1, D=0, \omega\right) \\
&= U^{\tau_3}\mathrm{E}\left(y(1,0) \mid S, D, \omega\right) - U^{\tau_3}\mathrm{E}\left(y(0,0) \mid S, D, \omega\right) + U^{\tau_3}\mathrm{E}\left(y(0,0) - \phi \mid S, D, \omega\right) \\
&\quad - U^{\tau_4}\mathrm{E}\left(y(0,0) - \phi \mid S, D, \omega\right) + U^{\tau_3}\mathrm{E}\left(\phi \mid S, D, \omega\right) - U^{\tau_4}\mathrm{E}\left(\phi \mid S, D, \omega\right) \\
&= U^{\tau_3}\mathrm{E}\left(y(1,0) \mid S, D, \omega\right) - U^{\tau_4}\mathrm{E}\left(\phi \mid S, D, \omega\right) - U^{\tau_3}\mathrm{E}\left(y(0,0) - \phi \mid S, D, \omega\right) + 0 \\
&= U^{\tau_3}\mathrm{E}\left(y(1,0) \mid S, D, \omega\right) - U^{\tau_4}\mathrm{E}\left(\phi \mid S, D, \omega\right) - U^{\tau_4}\mathrm{E}\left(y(0,0) - \phi \mid S, D, \omega\right) \\
&= U^{\tau_3}\mathrm{E}\left(y(1,0) \mid S, D, \omega\right) - U^{\tau_4}\mathrm{E}\left(y(0,0) \mid S, D, \omega\right),
\end{aligned}$$

where the third and fourth equalities follow from

$$U^{\tau_3}\mathrm{E}\left(y(0,0) - \phi \mid S, D, \omega\right) = U^{\tau_4}\mathrm{E}\left(y(0,0) - \phi \mid S, D, \omega\right)$$

by Assumption 5 (i). Similarly, we can get

$$\begin{aligned}
\beta + \xi &= \mathrm{E}\left(y(1,1) \mid S=1, D=1, \omega\right) - \mathrm{E}\left(y(0,0) \mid S=0, D=0, \omega\right) \\
&= U^{\tau_1}\mathrm{E}\left(y(1,1) \mid S, D, \omega\right) - U^{\tau_1}\mathrm{E}\left(y(0,0) \mid S, D, \omega\right) + U^{\tau_1}\mathrm{E}\left(y(0,0) - \phi \mid S, D, \omega\right) \\
&\quad - U^{\tau_2}\mathrm{E}\left(y(0,0) - \phi \mid S, D, \omega\right) + U^{\tau_1}\mathrm{E}\left(\phi \mid S, D, \omega\right) - U^{\tau_2}\mathrm{E}\left(\phi \mid S, D, \omega\right) \\
&= U^{\tau_1}\mathrm{E}\left(y(1,1) \mid S, D, \omega\right) - U^{\tau_2}\mathrm{E}\left(\phi \mid S, D, \omega\right) - U^{\tau_1}\mathrm{E}\left(y(0,0) - \phi \mid S, D, \omega\right) + 0 \\
&= U^{\tau_1}\mathrm{E}\left(y(1,1) \mid S, D, \omega\right) - U^{\tau_2}\mathrm{E}\left(\phi \mid S, D, \omega\right) - U^{\tau_2}\mathrm{E}\left(y(0,0) - \phi \mid S, D, \omega\right) \\
&= U^{\tau_1}\mathrm{E}\left(y(1,1) \mid S, D, \omega\right) - U^{\tau_2}\mathrm{E}\left(y(0,0) \mid S, D, \omega\right),
\end{aligned}$$

where the third and fourth equalities follow from

$$U^{\tau_1}\mathrm{E}\left(y(0,0) - \phi \mid S, D, \omega\right) = U^{\tau_2}\mathrm{E}\left(y(0,0) - \phi \mid S, D, \omega\right)$$

by Assumption 5 (i). Finally,

$$\begin{aligned}
\xi &= \mathrm{E}\left(y(1,1) \mid S=1, D=0, \omega\right) - \mathrm{E}\left(y(1,0) \mid S=1, D=0, \omega\right) \\
&= U^{\tau_1}\mathrm{E}\left(y(1,1) \mid S, D, \omega\right) - U^{\tau_1}\mathrm{E}\left(y(1,0) \mid S, D, \omega\right) + U^{\tau_1}\mathrm{E}\left(y(1,0) - \phi \mid S, D, \omega\right) \\
&\quad - U^{\tau_3}\mathrm{E}\left(y(1,0) - \phi \mid S, D, \omega\right) + U^{\tau_1}\mathrm{E}\left(\phi \mid S, D, \omega\right) - U^{\tau_3}\mathrm{E}\left(\phi \mid S, D, \omega\right) \\
&= U^{\tau_1}\mathrm{E}\left(y(1,1) \mid S, D, \omega\right) - U^{\tau_3}\mathrm{E}\left(\phi \mid S, D, \omega\right) - U^{\tau_1}\mathrm{E}\left(y(1,0) - \phi \mid S, D, \omega\right) + 0 \\
&= U^{\tau_1}\mathrm{E}\left(y(1,1) \mid S, D, \omega\right) - U^{\tau_3}\mathrm{E}\left(\phi \mid S, D, \omega\right) - U^{\tau_3}\mathrm{E}\left(y(1,0) - \phi \mid S, D, \omega\right) \\
&= U^{\tau_1}\mathrm{E}\left(y(1,1) \mid S, D, \omega\right) - U^{\tau_3}\mathrm{E}\left(y(1,0) \mid S, D, \omega\right),
\end{aligned}$$

where the third and fourth equalities follow from

$$U^{\tau_1}\mathrm{E}\left(y(1,0) - \phi \mid S, D, \omega\right) = U^{\tau_3}\mathrm{E}\left(y(1,0) - \phi \mid S, D, \omega\right)$$

by Assumption 5 (i).

(ii) The With-Without part. Based on the proof of part (i), we have

$$\begin{aligned}
\beta &= U^{\tau_3}\mathrm{E}\left(y(1,0) \mid S, D, \omega\right) - U^{\tau_4}\mathrm{E}\left(y(1,1) \mid S, D, \omega\right) \\
&= U^{\tau_3}\mathrm{E}\left(y(1,0) \mid S, D, \omega\right) - U^{\tau_4}\mathrm{E}\left(\phi \mid S, D, \omega\right) - U^{\tau_4}\mathrm{E}\left(y(0,0) - \phi \mid S, D, \omega\right) \\
&= U^{\tau_3}\mathrm{E}\left(y(1,0) \mid S, D, \omega\right) - U^{\tau_4}\mathrm{E}\left(\phi \mid S, D, \omega\right) - U^{\tau_3}\mathrm{E}\left(y(0,0) - \phi \mid S, D, \omega\right) \\
&= U^{\tau_3}\mathrm{E}\left(y(1,0) - y(0,0) \mid S, D, \omega\right) + U^{\tau_3}\mathrm{E}\left(\phi \mid S, D, \omega\right) - U^{\tau_4}\mathrm{E}\left(\phi \mid S, D, \omega\right) \\
&= U^{\tau_3}\mathrm{E}\left(y(1,0) - y(0,0) \mid S, D, \omega\right),
\end{aligned}$$

where the third equality comes from Assumption 5 (i) and the last equality follows from Assumption 5 (ii). Similarly, we will get

$$\begin{aligned}
\beta+\xi &= U^{\tau_1}\mathrm{E}\left(y(1,1)\mid S,D,\omega\right)-U^{\tau_2}\mathrm{E}\left(y(0,0)\mid S,D,\omega\right)\\
&= U^{\tau_1}\mathrm{E}\left(y(1,1)\mid S,D,\omega\right)-U^{\tau_2}\mathrm{E}\left(\phi\mid S,D,\omega\right)-U^{\tau_2}\mathrm{E}\left(y(0,0)-\phi\mid S,D,\omega\right)\\
&= U^{\tau_1}\mathrm{E}\left(y(1,1)\mid S,D,\omega\right)-U^{\tau_2}\mathrm{E}\left(\phi\mid S,D,\omega\right)-U^{\tau_1}\mathrm{E}\left(y(0,0)-\phi\mid S,D,\omega\right)\\
&= U^{\tau_1}\mathrm{E}\left(y(1,1)-y(0,0)\mid S,D,\omega\right)+U^{\tau_1}\mathrm{E}\left(\phi\mid S,D,\omega\right)-U^{\tau_2}\mathrm{E}\left(\phi\mid S,D,\omega\right)\\
&= U^{\tau_1}\mathrm{E}\left(y(1,1)-y(0,0)\mid S,D,\omega\right)
\end{aligned}$$

and

$$\begin{aligned}
\xi &= U^{\tau_1}\mathrm{E}\left(y(1,1)\mid S,D,\omega\right)-U^{\tau_3}\mathrm{E}\left(y(1,0)\mid S,D,\omega\right)\\
&= U^{\tau_1}\mathrm{E}\left(y(1,1)\mid S,D,\omega\right)-U^{\tau_3}\mathrm{E}\left(\phi\mid S,D,\omega\right)-U^{\tau_3}\mathrm{E}\left(y(1,0)-\phi\mid S,D,\omega\right)\\
&= U^{\tau_1}\mathrm{E}\left(y(1,1)\mid S,D,\omega\right)-U^{\tau_3}\mathrm{E}\left(\phi\mid S,D,\omega\right)-U^{\tau_1}\mathrm{E}\left(y(1,0)-\phi\mid S,D,\omega\right)\\
&= U^{\tau_1}\mathrm{E}\left(y(1,1)-y(1,0)\mid S,D,\omega\right)+U^{\tau_1}\mathrm{E}\left(\phi\mid S,D,\omega\right)-U^{\tau_3}\mathrm{E}\left(\phi\mid S,D,\omega\right)\\
&= U^{\tau_1}\mathrm{E}\left(y(1,1)-y(1,0)\mid S,D,\omega\right)
\end{aligned}$$

by Assumption 5 (i-ii). □

Proof of Theorem 3: Note that model (17) could be rewritten as $y=XB+E$ for $t=1,2,\ldots,t_S,\ldots,t_D-1$, where $X=(g(y'),S,Z,I)_{(t_D-1)\times 4}$, $g(y')=(g(y'(1))\cdots g(y'(t_D-1)))'$, $S=(S_1\cdots S_{t_D-1})'$, $Z=(Z_1\cdots Z_{t_D-1})'$, $I=(1\cdots 1)'_{(t_D-1)\times 1}$; $B=((a\eta+c\alpha),\beta,\gamma,e)'_{4\times 1}$; $y=(y_1\cdots y_{t_D-1})'$ and $E=(u_1+w_1+\varepsilon_1,\cdots,u_{t_D-1}+w_{t_D-1}+\varepsilon_{t_D-1})'$. Estimate model (17) by semiparametric methods and under some mild conditions (Assumption 2 (ii) and Assumption 3 (i)), we will get $\widehat{(a\eta+c\alpha)}=(a\eta+c\alpha)+o_p(1)$, $\widehat{\gamma}=\gamma+o_p(1)$ and $\widehat{e}=e+o_p(1)$ as $t_D-1\to\infty$. Define $Co\equiv\left(U^{\tau_2}\mathrm{E}\left(\widehat{y}\right)-U^{\tau_1}\mathrm{E}\left(\widehat{y}\right),0,A_4,0\right)_{1\times 4}$, after some simplifications, we can get

$$\begin{aligned}
\widehat{\beta} &= \frac{U^{\tau_2}\mathrm{E}\left(\widehat{Y}_{\mathrm{OLS}}\right)-U^{\tau_1}\mathrm{E}\left(\widehat{Y}_{\mathrm{OLS}}\right)-\widehat{(a\eta+c\alpha)}\left(U^{\tau_2}\mathrm{E}\left(\widehat{y}\right)-U^{\tau_1}\mathrm{E}\left(\widehat{y}\right)\right)-\widehat{\gamma}A_4}{A_1}\\
&= \frac{U^{\tau_2}\mathrm{E}\left(\widehat{Y}_{\mathrm{OLS}}\right)-U^{\tau_1}\mathrm{E}\left(\widehat{Y}_{\mathrm{OLS}}\right)-Co\left(X'X\right)^{-1}X'y}{A_1}\\
&= \frac{\beta A_1+\widehat{(a\eta+c\alpha)}\left(U^{\tau_2}\mathrm{E}\left(\widehat{y}\right)-U^{\tau_1}\mathrm{E}\left(\widehat{y}\right)\right)+\gamma A_4}{A_1}\\
&\quad-\frac{\left\{(a\eta+c\alpha)\left(U^{\tau_2}\mathrm{E}\left(\widehat{y}\right)-U^{\tau_1}\mathrm{E}\left(\widehat{y}\right)\right)+\gamma A_4+Co\left(X'X\right)^{-1}X'E\right\}}{A_1}\\
&= \beta-\frac{Co\left(X'X\right)^{-1}X'E}{A_1}+o_p(1).
\end{aligned}$$

We now prove that $Co\left(X'X\right)^{-1}X'E=o_p(1)$.

Define

$$\mathcal{M} \equiv X'X = \begin{pmatrix} \mathbb{S}_{yy} & \mathbb{S}_{yS} & \mathbb{S}_{yZ} & \mathbb{S}_{y} \\ \mathbb{S}_{yS} & \mathbb{S}_{SS} & \mathbb{S}_{SZ} & \mathbb{S}_{S} \\ \mathbb{S}_{yZ} & \mathbb{S}_{SZ} & \mathbb{S}_{ZZ} & \mathbb{S}_{Z} \\ \mathbb{S}_{y} & \mathbb{S}_{S} & \mathbb{S}_{Z} & t_D - 1 \end{pmatrix}, \quad X'E = \begin{pmatrix} \mathbb{S}_{\pi g(y')} \\ \mathbb{S}_{\pi s} \\ \mathbb{S}_{\pi Z} \\ \mathbb{S}_{\pi} \end{pmatrix}$$

where $\mathbb{S}_{yy} = \sum_{t=1}^{t_D-1} y_t y_t$, $\mathbb{S}_{yS}$ among others are defined in the same way; $\pi_t = u_t + w_t + \varepsilon_t$. We will then get

$$Co\left(X'X\right)^{-1} X'E = \frac{1}{\det(\mathcal{M})} \left(\left\{U^{\tau_2}\mathrm{E}\left(\widehat{y}\right) - U^{\tau_1}\mathrm{E}\left(\widehat{y}\right)\right\} \cdot \mathbb{C}_{21} \cdot \mathbb{S}_{\pi s} + A_4 \cdot \mathbb{C}_{23} \cdot \mathbb{S}_{\pi s}\right)$$

where $\mathbb{C}_{ij} = (-1)^{i+j}\det(\mathcal{M}_{ij})$, $\mathcal{M}_{ij}$ is the (i,j)-th cofactor of the matrix $\mathcal{M}$, $\tau_1 = \{1, 2, \ldots, t_S - 1\}$, $\tau_2 = \{t_S, \ldots, t_D - 1\}$ and $\tau = \{1, 2, \ldots, t_D - 1\}$.

$$\begin{aligned} \mathbb{C}_{21} &= -1\det(\mathcal{M}_{21}) \\ &= -(t_D - t_S)(t_S - 1)\Big\{ \left(\mathbb{S}_{ZZ} - (t_D-1)U^{\tau}\mathrm{E}(Z)^2\right)\left(U^{\tau_2}\mathrm{E}(y) - U^{\tau}\mathrm{E}(y)\right) \\ &\qquad - \left(\mathbb{S}_{yZ} - (t_D-1)U^{\tau}\mathrm{E}(y)U^{\tau}E(Z)\right)\left(U^{\tau_2}\mathrm{E}(Z) - U^{\tau}\mathrm{E}(Z)\right)\Big\}, \end{aligned}$$

hence

$$\begin{aligned} \frac{1}{(t_D-1)^3}\mathbb{C}_{21} = &-\frac{(t_D - t_S)(t_S - 1)}{(t_D-1)^2}U^{\tau}\mathrm{E}\left(Z^2\right)\left(U^{\tau_2}\mathrm{E}(y) - U^{\tau}\mathrm{E}(y)\right) \\ &+ \frac{(t_D - t_S)(t_S - 1)}{(t_D-1)^2}\left(U^{\tau}E(Z)\right)^2\left(U^{\tau_2}\mathrm{E}(y) - U^{\tau}\mathrm{E}(y)\right) \\ &+ \frac{(t_D - t_S)(t_S - 1)}{(t_D-1)^2}U^{\tau}\mathrm{E}(yZ)\left(U^{\tau_2}\mathrm{E}(Z) - U^{\tau}\mathrm{E}(Z)\right) \\ &- \frac{(t_D - t_S)(t_S - 1)}{(t_D-1)^2}U^{\tau}E(y)U^{\tau}E(Z)\left(U^{\tau_2}\mathrm{E}(Z) - U^{\tau}\mathrm{E}(Z)\right). \end{aligned}$$

As $t_D - 1 \to \infty$, under Assumption 7,

$$\frac{1}{(t_D-1)^3}\mathbb{C}_{21} = o_p(1).$$

Similarly,

$$\begin{aligned} \mathbb{C}_{23} = -(t_D-1)(t_D-t_S)\Big\{ &\left(\mathbb{S}_{yy} - (t_D-1)U^{\tau_2}\mathbb{E}(y)\right)\left(U^{\tau_2}\mathrm{E}(Z) - U^{\tau}\mathrm{E}(Z)\right) \\ &- \left(\mathbb{S}_{yZ} - (t_D-1)U^{\tau}E(y)U^{\tau}E(Z)\right)\left(U^{\tau_2}\mathrm{E}(y) - U^{\tau}\mathrm{E}(y)\right)\Big\}, \end{aligned}$$

$$\begin{aligned} \frac{1}{(t_D-1)^3}\mathbb{C}_{23} = &-\frac{t_D - t_S}{t_D - 1}U^{\tau}\mathrm{E}\left(y^2\right)\left(U^{\tau_2}\mathrm{E}(Z) - U^{\tau}\mathrm{E}(Z)\right) \\ &+ \frac{t_D - t_S}{t_D - 1}U^{\tau}\mathrm{E}(y)^2\left(U^{\tau_2}\mathrm{E}(Z) - U^{\tau}\mathrm{E}(Z)\right) \\ &+ \frac{t_D - t_S}{t_D - 1}U^{\tau}\mathrm{E}(yZ)\left(U^{\tau_2}\mathrm{E}(y) - U^{\tau}\mathrm{E}(y)\right) \\ &- \frac{t_D - t_S}{t_D - 1}U^{\tau}E(y)U^{\tau}E(Z)\left(U^{\tau_2}\mathrm{E}(y) - U^{\tau}\mathrm{E}(y)\right), \end{aligned}$$

as $t_D - 1 \to \infty$, under Assumption 7,

$$\frac{1}{(t_D-1)^3}\mathbb{C}_{23} = o_p(1).$$

Note that

$$\begin{aligned}
\det(\mathcal{M}) = & \mathbb{S}_{yy}\det(\mathcal{M}_{11}) - \mathbb{S}_{yS}\det(\mathcal{M}_{12}) + \mathbb{S}_{yZ}\det(\mathcal{M}_{13}) - \mathbb{S}_{y}\det(\mathcal{M}_{14}) \\
= & \mathbb{S}_{yy}\Big\{(t_D - t_S)\big(\mathbb{S}_{ZZ}(t_D-1) - \mathbb{S}_Z\mathbb{S}_Z\big) - \mathbb{S}_{ZS}\big(\mathbb{S}_{ZS}(t_D-1) - \mathbb{S}_Z(t_D-t_S)\big) \\
& + (t_D-t_S)\big(\mathbb{S}_{ZS}\mathbb{S}_Z - \mathbb{S}_{ZZ}(t_D-t_S)\big)\Big\} \\
& - \mathbb{S}_{yS}\Big\{\mathbb{S}_{yS}\big(\mathbb{S}_{ZZ}(t_D-1) - \mathbb{S}_Z\mathbb{S}_Z\big) - \mathbb{S}_{ZS}\big(\mathbb{S}_{yZ}(t_D-1) - \mathbb{S}_Z\mathbb{S}_y\big) \\
& + (t_D-t_S)\big(\mathbb{S}_{yZ}\mathbb{S}_Z - \mathbb{S}_{ZZ}\mathbb{S}_y\big)\Big\} \\
& + \mathbb{S}_{yZ}\Big\{\mathbb{S}_{yS}\big(\mathbb{S}_{ZS}(t_D-1) - \mathbb{S}_Z(t_D-t_S)\big) - (t_D-t_S)\big(\mathbb{S}_{yZ}(t_D-1) - \mathbb{S}_Z\mathbb{S}_y\big) \\
& + (t_D-t_S)\big(\mathbb{S}_{yZ}(t_D-t_S) - \mathbb{S}_{ZS}\mathbb{S}_y\big)\Big\} \\
& - \mathbb{S}_{y}\Big\{\mathbb{S}_{yS}\big(\mathbb{S}_{ZS}\mathbb{S}_Z - \mathbb{S}_{ZZ}(t_D-t_S)\big) - (t_D-t_S)\big(\mathbb{S}_{yZ}\mathbb{S}_Z - \mathbb{S}_{ZZ}\mathbb{S}_y\big) \\
& + \mathbb{S}_{ZS}\big(\mathbb{S}_{yZ}(t_D-t_S) - \mathbb{S}_{ZS}\mathbb{S}_y\big)\Big\},
\end{aligned}$$

hence

$$
\begin{aligned}
\frac{1}{(t_D-1)^3}\det(\mathcal{M}) = {} & (t_D-t_S)U^\tau \mathrm{E}\left(y^2\right)U^\tau \mathrm{E}\left(Z^2\right) \\
& -(t_D-t_S)U^\tau \mathrm{E}\left(y^2\right)U^\tau \mathrm{E}(Z)^2 \\
& -U^\tau \mathrm{E}\left(y^2\right)\frac{\mathbb{S}_{ZS}\mathbb{S}_{ZS}}{t_D-1} \\
& +\frac{t_D-t_S}{t_D-1}U^\tau \mathrm{E}\left(y^2\right)U^\tau \mathrm{E}(Z)\frac{\mathbb{S}_{ZS}}{t_D-1} \\
& +\frac{t_D-t_S}{t_D-1}U^\tau \mathrm{E}\left(y^2\right)U^\tau \mathrm{E}(Z)\mathbb{S}_{ZS} \\
& -\frac{(t_D-t_S)^2}{t_D-1}U^\tau \mathrm{E}\left(y^2\right)U^\tau \mathrm{E}\left(Z^2\right) \\
& -U^\tau \mathrm{E}\left(Z^2\right)\frac{\mathbb{S}_{yS}\mathbb{S}_{yS}}{t_D-1} \\
& +U^\tau \mathrm{E}(Z)^2\frac{\mathbb{S}_{yS}\mathbb{S}_{yS}}{t_D-1} \\
& +U^\tau \mathrm{E}(yZ)\frac{\mathbb{S}_{yS}\mathbb{S}_{ZS}}{t_D-1} \\
& -U^\tau E(y)U^\tau E(Z)\frac{\mathbb{S}_{yS}\mathbb{S}_{ZS}}{t_D-1} \\
& -\frac{t_D-t_S}{t_D-1}U^\tau \mathrm{E}(yZ)U^\tau \mathrm{E}(Z)\mathbb{S}_{yS} \\
& +\frac{t_D-t_S}{t_D-1}U^\tau \mathrm{E}\left(Z^2\right)U^\tau E(y)\mathbb{S}_{yS} \\
& +\frac{(t_D-t_S)^2}{t_D-1}U^\tau \mathrm{E}(yZ)^2 \\
& -\frac{t_D-t_S}{t_D-1}U^\tau \mathrm{E}(yZ)U^\tau \mathrm{E}(y)\mathbb{S}_{ZS} \\
& -U^\tau E(y)U^\tau E(Z)\frac{\mathbb{S}_{yS}\mathbb{S}_{ZS}}{t_D-1} \\
& +\frac{t_D-t_S}{t_D-1}U^\tau \mathrm{E}(y)U^\tau \mathrm{E}\left(Z^2\right) \\
& +\frac{t_D-t_S}{t_D-1}U^\tau \mathrm{E}(y)U^\tau \mathrm{E}(yZ)U^\tau \mathrm{E}(Z) \\
& -\frac{t_D-t_S}{t_D-1}U^\tau \mathrm{E}(y)^2U^\tau \mathrm{E}\left(Z^2\right) \\
& -\frac{t_D-t_S}{t_D-1}U^\tau \mathrm{E}(y)U^\tau \mathrm{E}(yZ)\mathbb{S}_{yS} \\
& +U^\tau \mathrm{E}(y)^2\frac{\mathbb{S}_{ZS}}{t_D-1} \\
& +U^\tau \mathrm{E}(yZ)\frac{\mathbb{S}_{yS}\mathbb{S}_{ZS}}{t_D-1} \\
& -\frac{t_D-t_S}{t_D-1}U^\tau \mathrm{E}(yZ)U^\tau \mathrm{E}(Z)\mathbb{S}_{yS} \\
& -(t_D-t_S)U^\tau \mathrm{E}(yZ)U^\tau \mathrm{E}(y)U^\tau \mathrm{E}(Z),
\end{aligned}
$$

as $t_D - 1 \to \infty$, under Assumption 7,

$$\begin{aligned}
\frac{1}{(t_D-1)^3}\det(\mathcal{M}) = {} & (t_D - t_S)\Big\{U^\tau \mathrm{E}\left(y^2\right) U^\tau \mathrm{E}\left(Z^2\right) - U^\tau \mathrm{E}\left(y^2\right) U^\tau \mathrm{E}(Z)^2 - U^\tau \mathrm{E}(yZ)^2 \\
& \qquad + U^\tau \mathrm{E}(yZ) U^\tau \mathrm{E}(y) U^\tau \mathrm{E}(Z)\Big\} \\
& + \frac{1}{t_D-1}\Big\{ - U^\tau \mathrm{E}\left(y^2\right) \mathbb{S}_{ZS}^2 - U^\tau \mathrm{E}\left(Z^2\right) \mathbb{S}_{yS}^2 + U^\tau \mathrm{E}(Z)^2 \mathbb{S}_{yS}^2 \\
& \qquad + U^\tau \mathrm{E}(yZ) \mathbb{S}_{yS} \mathbb{S}_{ZS} - U^\tau \mathrm{E}(y) U^\tau \mathrm{E}(Z) \mathbb{S}_{yS} \mathbb{S}_{ZS} \\
& \qquad - U^\tau \mathrm{E}(y) U^\tau \mathrm{E}(Z) \mathbb{S}_{yS} \mathbb{S}_{ZS} + U^\tau \mathrm{E}(y) U^\tau \mathrm{E}(Z) \mathbb{S}_{ZS} \\
& \qquad + U^\tau \mathrm{E}(yZ) \mathbb{S}_{yS} \mathbb{S}_{ZS}\Big\} \\
& + \frac{(t_D - t_S)^2}{t_D - 1}\Big\{ - U^\tau \mathrm{E}\left(y^2\right) U^\tau \mathrm{E}\left(Z^2\right) + U^\tau \mathrm{E}(yZ)^2 \Big\} + o_p(1) \\
< {} & \infty.
\end{aligned}$$

Hence,

$$Co\left(X'X\right)^{-1} X'E = \frac{\left\{U^{\tau_2} \mathrm{E}\left(\widehat{y}\right) - U^{\tau_1} \mathrm{E}\left(\widehat{y}\right)\right\} \cdot \frac{1}{(t_D-1)^3} \mathbb{C}_{21} \cdot \mathbb{S}_{\pi s} + A_4 \cdot \frac{1}{(t_D-1)^3} \mathbb{C}_{23} \cdot \mathbb{S}_{\pi s}}{\frac{1}{(t_D-1)^3} \det(\mathcal{M})} = o_p(1)$$

as $t_D - 1 \to \infty$. In summary, we finally obtain $\mathrm{E}(\widehat{\beta}) = \beta$ and $\widehat{\beta} = \beta + o_p(1)$. $\square$

Lemma S.2 Suppose that $y_t = m\left(D_t, X_t, \varepsilon_t\right)$ with $D_t = h\left(X_t\right)$, ε_t is independent of D_t and X_t, then $\mathrm{E}(\varepsilon) = 0$ if and only if ε_t is separable from $m(\cdot)$, i.e. there exist a continuous and invertible function $m_1(\cdot, \cdot)$ such that $y_t = m_1\left(D_t, X_t\right) + \varepsilon_t$ almost surely. Furthermore more, we will then get $\mathrm{E}(y|D, X) = \mathrm{E}(y|X)$.

Proof of Lemma S.2: By the definitions of Lemma S.2 and Matzkin (2003), we have $y = m\left(D, X, \varepsilon\right) = \mathrm{E}(y|D, X) + \eta$ with $\mathrm{E}(\eta|D, X) = 0$. Then

$$\mathrm{E}\left[\left\{m\left(D, X, \varepsilon\right) - \mathrm{E}(y|D, X)\right\} \mid D, X\right] = 0. \tag{S.1}$$

By the law of iterated expectations,

$$\mathrm{E}\left(m\left(D, X, \varepsilon\right)\right) = \iint \mathrm{E}\left(m\left(D, X, \varepsilon\right) \mid D, X\right) f\left(X, D\right) dX dD, \tag{S.2}$$

where $f\left(X, D\right)$ is the joint probability density function of the random variables (X, D). Then one can verify that $\mathrm{E}(\varepsilon) = 0$ if and only if $m\left(D_t, X_t, \varepsilon_t\right) = m_1\left(D_t, X_t\right) + \varepsilon_t$ almost surely. Based on this, we obtain

$$\mathrm{E}(y|D, X) = \mathrm{E}(m_1\left(D, X\right) + \varepsilon|D, X) = m_1\left(D, X\right), \tag{S.3}$$

and

$$\begin{aligned}\int \mathrm{E}(y|D,X)f(X)dX &= \int m_1\left(D,X\right)f(X)dX \\ &= \int m_1\left(h\left(X\right),X\right)f(X)dX \\ &= \mathrm{E}\left(m_1\left(D,X\right)\right) \\ &= \int \mathrm{E}(y|X)f(X)dX.\end{aligned}$$

We finally get $\mathrm{E}(y|D,X)=\mathrm{E}(y|X)$. □

Proof of Theorem 2: For estimation Step 3.1, note first that $\mathrm{E}\left(y(1,1)S \mid \widehat{y}'\right) = \mathrm{E}\left(y(1,1) \mid \widehat{y}'\right)$ implies $\mathrm{E}\left(y(0,0)S \mid \widehat{y}'\right) = \mathrm{E}\left(y(0,0) \mid \widehat{y}'\right)$ because

$$y_t = y_t(1,1) = y_t(0,0) + \beta \cdot I\{t_S \le t \le t_D - 1\}$$

by model (10) for $t = 1,2,\ldots,t_S,\ldots,t_D-1$. For a given relationship $y_t = m\left(S_t, \widehat{y}'_t, \varepsilon_t\right)$, by Lemma S.2, if we can proof ε_t is separable from $m(\cdot)$ and $\mathrm{E}\left(\varepsilon\right)=0$, then we can get $\mathrm{E}\left(y^{(1,1)}S \mid \widehat{y}', S\right) = \mathrm{E}\left(y^{(1,1)} \mid \widehat{y}'\right)$. For the estimation Step 3.1, one can verify that

$$\widehat{\beta}_{y,2} = \frac{c_1\sum_{\ell\in T} t_\ell^2 y_\ell + c_2\sum_{\ell\in T} t_\ell y_\ell + c_3\sum_{\ell\in T} y_\ell}{\widetilde{c}_1\sum_{\ell\in T} t_\ell^4 + \widetilde{c}_2\sum_{\ell\in T} t_\ell^3 + \widetilde{c}_3\sum_{\ell\in T} t_\ell^2}, \tag{S.4}$$

$$\widehat{\beta}_{y,1} = \frac{b_1\sum_{\ell\in T} t_\ell^2 y_\ell + b_2\sum_{\ell\in T} t_\ell y_\ell + b_3\sum_{\ell\in T} y_\ell}{\widetilde{b}_1\sum_{\ell\in T} t_\ell^4 + \widetilde{b}_2\sum_{\ell\in T} t_\ell^3 + \widetilde{b}_3\sum_{\ell\in T} t_\ell^2}, \tag{S.5}$$

with

$$\left\{\begin{array}{l} c_1 = \left(\sum_{\ell\in T} t_\ell^2 (t_D-1) - \sum_{\ell\in T} t_\ell \sum_{\ell\in T} t_\ell\right) \\ c_2 = \left(\sum_{\ell\in T} t_\ell \sum_{\ell\in T} t_\ell^2 - \sum_{\ell\in T} t_\ell^3 (t_D-1)\right) \\ c_3 = \left(\sum_{\ell\in T} t_\ell^2 \sum_{\ell\in T} t_\ell^2 - \sum_{\ell\in T} t_\ell^3 \sum_{\ell\in T} t_\ell\right) \end{array}\right., \quad \left\{\begin{array}{l} \widetilde{c}_1 = \left(\sum_{\ell\in T} t_\ell^2 (t_D-1) - \sum_{\ell\in T} t_\ell \sum_{\ell\in T} t_\ell\right) \\ \widetilde{c}_2 = \left(\sum_{\ell\in T} t_\ell \sum_{\ell\in T} t_\ell^2 - \sum_{\ell\in T} t_\ell^3 (t_D-1)\right) \\ \widetilde{c}_3 = \left(\sum_{\ell\in T} t_\ell^2 \sum_{\ell\in T} t_\ell^2 - \sum_{\ell\in T} t_\ell^3 \sum_{\ell\in T} t_\ell\right) \end{array}\right.;$$

$$\left\{\begin{array}{l} b_1 = \left(\sum_{\ell\in T} t_\ell \sum_{\ell\in T} t_\ell^2 - \sum_{\ell\in T} t_\ell^3 (t_D-1)\right) \\ b_2 = \left(\sum_{\ell\in T} t_\ell^4 (t_D-1) - \sum_{\ell\in T} t_\ell^2 \sum_{\ell\in T} t_\ell^2\right) \\ b_3 = \left(\sum_{\ell\in T} t_\ell^2 \sum_{\ell\in T} t_\ell^2 - \sum_{\ell\in T} t_\ell^4 \sum_{\ell\in T} t_\ell\right) \end{array}\right., \quad \left\{\begin{array}{l} \widetilde{b}_1 = \left(\sum_{\ell\in T} t_\ell \sum_{\ell\in T} t_\ell - \sum_{\ell\in T} t_\ell^2 (t_D-1)\right) \\ \widetilde{b}_2 = \left(\sum_{\ell\in T} t_\ell^3 (t_D-1) - \sum_{\ell\in T} t_\ell \sum_{\ell\in T} t_\ell^2\right) \\ \widetilde{b}_3 = \left(\sum_{\ell\in T} t_\ell^3 \sum_{\ell\in T} t_\ell - \sum_{\ell\in T} t_\ell^2 \sum_{\ell\in T} t_\ell^2\right) \end{array}\right.;$$

and

$$\begin{aligned}\widehat{y}'_t &= \frac{\widehat{a}_{y,2}S'(t) + \widehat{a}_{S,2}\widehat{a}_{y,1} - \widehat{a}_{y,2}\widehat{a}_{S,1}}{\widehat{a}_{S,2}} \\ &= 2\widehat{\beta}_{y,2}t + 2\widehat{\beta}_{y,1} - \frac{\widehat{\beta}_{y,2}\widehat{\beta}_{S,1}}{\widehat{\beta}_{S,2}} \\ &= 2\left(c_1t^2 + c_2t + c_3I\right)' y_t t - 2\left(b_1t^2 + b_2t + b_3I\right)' y_t - \frac{\widehat{\beta}_{S,1}}{\widehat{\beta}_{S,2}}\left(c_1t^2 + c_2t + c_3I\right)' y_t\end{aligned}$$

where $t^2 = \left(t_1^2, t_2^2, \ldots, t_{t_D-1}^2\right)'$, $t = (t_1, t_2, \ldots, t_{t_D-1})'$, $I = (1, 1, \ldots, 1)'_{(t_D-1)\times 1}$ and $T = \{1, 2, \ldots, t_D - 1\}$. Hence, it can be seen from the above equation that there is a linear relationship between $\widehat{y}_t'$ and y_t, which could be written as

$$y_t = m\left(\widehat{y}_t', S_t, \epsilon_t\right) = a(t)\widehat{y}_t' + c(t)S_t + b(t) + \epsilon_t. \tag{S.6}$$

for some $a(t), b(t), c(t) \in \mathcal{L}_p$. As we can see here, ϵ_t in (S.6) is separable from $m(\cdot)$, which indicates that if we can further prove that $\mathrm{E}(\epsilon) = 0$, we will finally get $\mathrm{E}\left(y(1,1)S \mid \widehat{y}', S\right) = \mathrm{E}\left(y(1,1) \mid \widehat{y}'\right)$ by Lemma S.2. For the model (S.6), we have

$$\begin{aligned}
\mathrm{E}(y) &= \int \mathrm{E}(y \mid \widehat{y}') f(\widehat{y}') d\widehat{y}' \\
&= \int \left\{a(t)\widehat{y}' f(\widehat{y}') + c(t)\mathrm{E}\left(S \mid \widehat{y}'\right) f(\widehat{y}') + b(t) f(\widehat{y}') + \mathrm{E}\left(\epsilon \mid \widehat{y}'\right) f(\widehat{y}')\right\} d\widehat{y}' \\
&= a(t)\mathrm{E}(\widehat{y}') + c(t)\mathrm{E}\left(S\right) + b(t) + \mathrm{E}(\epsilon),
\end{aligned}$$

hence there always exist $a(t), b(t), c(t) \in \mathcal{L}_p$ such that

$$\mathrm{E}(\epsilon) = \mathrm{E}(y) - a(t)\mathrm{E}(\widehat{y}') - c(t)\mathrm{E}\left(S\right) - b(t) = 0.$$

The proof of Step 3.2 is analogous to Step 3.1, hence omitted here. □

Lemma S.3 Suppose that $r(k) = k^{-D}\mathcal{L}(k)$ with $0 < D < q^{-1}$, where q is the Hermite rank of G, and $\mathcal{L} : (0, \infty) \to \mathbb{R}$ is a slowly varying function at infinity, i.e. $\lim_{t\to\infty} \mathcal{L}(\tau k)/\mathcal{L}(k) = 1$. Assume that $B \to \infty$, then we have

$$\frac{\left(\mathrm{Var}_B\left(\widehat{\beta}\right) - \sigma_\beta^2\right)}{d_B} = o_p(1)$$

for the bootstrap sequence $\widetilde{B} = \{\widehat{\beta}_1, \widehat{\beta}_2, \ldots, \widehat{\beta}_B\}$.

Proof of Lemma S.3: Note that by Taqqu (1975) and Dobrushin & Major (1979), we have $\sigma_{\beta\eta}^2 = o\left(d_B\right)$, $\mathrm{E}\left\{H_k(\widehat{\beta}_i)H_k(\widehat{\beta}_j)\right\} = r\left(i-j\right)^k I\{i = j\}$, $\mathrm{E}\left(\widetilde{G}\left(\widehat{\beta}_b\right)\right) = G\left(\widehat{\beta}_b\right) - c_q H_q(\widehat{\beta}_b)$ and

$$\begin{aligned}
d_B^{-1}\,\mathrm{Var}_B\left(\widehat{\beta}_b\right) &= \left(Bd_B\right)^{-1}\mathrm{E}\left\{\sum_{b=1}^{B}\left(\widehat{\beta}_b - \mu_\beta\right)^2\right\} \\
&\leq \left(Bd_B\right)^{-1}\mathrm{E}\left\{\sum_{b=1}^{B}\left|\widehat{\beta}_b - \mu_\beta\right|\right\}^2 \\
&= \left(Bd_B\right)^{-1}\mathrm{E}\left\{\sum_{i=1}^{B}\sum_{j=1}^{B}\left(2 - I\{i = j\}\left|\widehat{\beta}_i - \mu_\beta\right|\left|\widehat{\beta}_j - \mu_\beta\right|\right)\right\}.
\end{aligned}$$

Hence, it follows that

$$
\begin{aligned}
d_B^{-1}\operatorname{Var}_B\left(\widehat{\beta}_b\right) &\le (Bd_B)^{-1}\left\{|c_q|\sum_{i=1}^{B}\sum_{j=1}^{B}\left(\left|r(i-j)-\widehat{\beta}_i\widehat{\beta}_j\right|+\mu_{\beta\eta}^2+|\mu_\beta\left(\mathrm{E}\left(\widehat{\beta}_i\right)+\mathrm{E}\left(\widehat{\beta}_j\right)\right)|\right)^q\right.\\
&\quad\left.+B^2\sum_{i=1}^{B}\sum_{j=1}^{B}\left|\mathrm{E}\left(\widetilde{G}\left(\widehat{\beta}_i\right)\right)\mathrm{E}\left(\widetilde{G}\left(\widehat{\beta}_j\right)\right)\right|\right\}\\
&\le C(c_q)\,(Bd_B)^{-1}B^2\sum_{i=1}^{B}\sum_{j=1}^{B}\left|r(i-j)-\widehat{\beta}_i\widehat{\beta}_j+\mu_\beta^2+\mathrm{E}\left(\widehat{\beta}_i\right)+\mathrm{E}\left(\widehat{\beta}_j\right)\right|^q\cdot(1+o(1))\\
&= o(1)
\end{aligned}
$$

where $C(\cdot)$ denotes a generic constant depending only on its argument. Therefore, by stationarity of the sequence $\widetilde{B}$, we finally get $d_B^{-1}\operatorname{Var}_B\left(\widehat{\beta}\right)=o_p(1)$. □

Lemma S.4 Let $\widehat{W}_B^2=\frac{\mathrm{E}_B\left(\widehat{\beta}-\sigma_\beta^2\right)^2}{d_B^2}$ and assume that the conditions of Lemma S.3 hold true, then $\widehat{W}_B^2=W_q^2+o_p(1)$ as $B\to\infty$.

Proof of Lemma S.4: Define $\widehat{W}_{1,B}^2=\left(Bd_B^2\right)^{-2}\sum_{j=1}^{B}\left(c_qH_q(\widehat{\beta}_j)\right)^2$, and

$$
\widehat{W}_{2,B}^2=\left(Bd_B^2\right)^{-2}\sum_{j=1}^{B}\widetilde{G}\left(\widehat{\beta}_j\right)^2=\left(Bd_B^2\right)^{-2}\sum_{j=1}^{B}\left\{\left(\widehat{\beta}_j-\mu_\beta\right)^2-\sigma_\beta^2-c_qH_q(\widehat{\beta}_j)\right\}^2.
$$

Then $\left|\widehat{W}_B^2-\widehat{W}_{1,B}^2\right|\le\widehat{W}_{2,B}^2+2\left|\widehat{W}_{1,n}\widehat{W}_{2,n}\right|$. By Corollary 3.1 of Taqqu (1975), and the stationarity of the sequence $\widetilde{B}$, we can get

$$
\begin{aligned}
\operatorname{Var}_B\left(\widehat{W}_{1,B}^2\right) &\le Cc_q^2\left(Bd_B^2\right)^{-2}\sum_{j=0}^{B-1}(B-j)\left|\left(\left(\sum_{i=1}^{B}H_q(\widehat{\beta}_i)\right)^2,\left(\sum_{i=1}^{B}H_q(\widehat{\beta}_{i+j-1})\right)^2\right)\right|\\
&\le C(c_q)\left(Bd_B^4\right)^{-1}\sum_{j=0}^{B-1}\sum_{i_1=1}^{B}\sum_{i_2=1}^{B}\sum_{i_3=j}^{j+B-1}\sum_{i_4=j}^{j+B-1}\\
&\qquad\times\left|(q!)^4\,2^{-2q}\,(2q!)^{-1}\sum_{1}\prod_{k=1}^{2q}r\left({i'}_k-{j'}_k\right)-(q!)^2\,r\left(i_1-i_2\right)^q r\left(i_3-i_4\right)^q\right|
\end{aligned}
$$

where the operator Σ_1 extends over all $\left({i'}_1,{j'}_1\right),\ldots,\left({i'}_{2q},{j'}_{2q}\right)\in\{i_1,\ldots,i_4\}$ such that ${i'}_k\ne{j'}_k$ for all $k=1,\ldots,2q$ and there are exactly q indices among $\{({i'}_k,{j'}_k):1\le k\le 2q\}$ that are equal to i_1,i_2,i_3,i_4 respectively. Write now $\Sigma_1=\Sigma_{11}+\Sigma_{12}$ where Σ_{11} extends over all indices $\{({i'}_k,{j'}_k):1\le k\le 2q\}$ under Σ_1 for which $|{i'}_k-{j'}_k|=|i_1-i_2|$ for q pairs and $|{i'}_k-{j'}_k|=|i_3-i_4|$ for the

remaining q pairs. Hence, we claim that the number of such indices under Σ_{11} is $2^{-2q}(2q!)(q!)^{-2}$. We then get

$$\left|(q!)^4 2^{-2q}(2q!)^{-1}\sum_1\prod_{k=1}^{2q} r\left(i'_k - j'_k\right) - (q!)^2 r\left(i_1 - i_2\right)^q r\left(i_3 - i_4\right)^q\right|$$
$$\leq C(c_q)\sum_{12}\prod_{k=1}^{2q} r\left(i'_k - j'_k\right)$$

where Σ_{12} extends over the rest of the indices under Σ_1. To prove the claim, note that for any $\left\{\left(i'_1, j'_1\right), \ldots, \left(i'_{2q}, j'_{2q}\right)\right\}$ under Σ_1, if $|i'_k - j'_k| = |i_1 - i_2|$ for some $k_1, \ldots, k_q \in \{1, \ldots, 2q\}$, then

(i) $|i'_k - j'_k| = |i_3 - i_4|$ for all $k \in \{1, \ldots, 2q\} \setminus \{k_1, \ldots, k_q\}$; and

(ii) exactly q of $\left\{\left(i'_{k_1}, j'_{k_1}\right), \ldots, \left(i'_{k_q}, j'_{k_q}\right)\right\}$ are i_k, $k = 1, 2$ and exactly q of the remaining $2q$ integers are i_k, $k = 3, 4$.

Using this, we can check that the set of all indices $\left\{\left(i'_1, j'_1\right), \ldots, \left(i'_{2q}, j'_{2q}\right)\right\}$ under Σ_{11} can be obtained by first selecting a subset $\{k_1, \ldots, k_q\}$ of size q from $\{1, \ldots, 2q\}$, and then setting $(i'_k, j'_k) = (i_1, i_2)$ or (i_2, i_1) for $k \in \{k_1, \ldots, k_q\}$ and $(i'_k, j'_k) = (i_3, i_4)$ or (i_4, i_3) for $k \in \{1, \ldots, 2q\} \setminus \{k_1, \ldots, k_q\}$. Hence the number of terms under Σ_{11} is $2^{-2q}(2q!)(q!)^{-2}$, proving the claim.

Next define $\mathbb{N} = B^\delta$ where δ is any real number satisfying $0 < \delta < \varepsilon(5 - 2qD)^{-1}$ for some $0 < \varepsilon < 1$. Let $\overline{r}(j) = |j|^{-D}(1 + |\mathcal{L}(|j|)|)$, $j \in \mathbb{Z}$ and $M_B = \max\{1 + |\mathcal{L}(j)| : 1 \leq j \leq B\}$. Then for enough large B,

$$\max\{|r(k)| : \mathbb{N} \leq k \leq B\} \leq C(D, \varepsilon)\overline{r}(j) B^{-\delta D} M_B$$

uniformly over $1 \leq j \leq B$. Furthermore, given any i_1, i_2, i_3, i_4, for every multi-index $\{(i'_k, j'_k) : 1 \leq k \leq 2q\}$ under Σ_{12},

$$|i'_k - j'_k| \geq \min\{|i_1 - i_3|, |i_1 - i_4|, |i_2 - i_3|, |i_2 - i_4|\} \geq j$$

for at least one $k \in \{1, \ldots, 2q\}$. Hence, from (S1.9) and (S1.10), it follows that

$$\mathrm{Var}_B\left(\widehat{W}^2_{1,B}\right) \leq C(c_q, q)\left(Bd_B^4\right)^{-1}\sum_{j=0}^{B-1}\sum_{i_1=1}^{B}\sum_{i_2=1}^{B}\sum_{i_3=j}^{j+B-1}\sum_{i_4=j}^{j+B-1}\sum_{12}\prod_{k=1}^{2q} r\left(i'_k - j'_k\right)$$
$$\leq C(c_q, q)\left(Bd_B^4\right)^{-1} J_1 + o\left(B^{-\delta} M_B\right)$$

where $J_1 = \sum_{j=0}^{B-1}\sum_{i_1=1}^{B}\sum_{i_2=1}^{B}\sum_{i_3=j}^{j+B-1}\sum_{i_4=j}^{j+B-1}\sum_{12}\prod_{k=1}^{2q} r\left(i'_k - j'_k\right)$. It is now can be seen that for any i_1, i_2, i_3, i_4, we have

$$\sum_1\prod_{k=1}^{2q} r\left(i'_k - j'_k\right) \leq C(q)\sum_3 \overline{r}\left(i_1 - i_2\right)^{q_1}\overline{r}\left(i_1 - i_3\right)^{q_2}\overline{r}\left(i_1 - i_4\right)^{q_3}$$
$$\times\, \overline{r}\left(i_2 - i_3\right)^{q_4}\overline{r}\left(i_2 - i_4\right)^{q_5}\overline{r}\left(i_3 - i_4\right)^{q_6}$$

where Σ_3 extends over all non-negative integers $q_1, \ldots, q_6$ satisfying $q_1 + q_2 + q_3 = q$, $q_1 + q_4 + q_5 = q$, $q_2 + q_4 + q_6 = q$ and $q_3 + q_5 + q_6 = q$. Next, writing $a = \frac{(1-qD)}{q}$

and $d = \frac{(2-qD)}{D}$. Using Holder's inequality and the conditions on $q_1, \ldots, q_6$, we can get, for any $1 \le j \le B$,

$$\sum_{i_3=j}^{j+B-1} \sum_{i_4=j}^{j+B-1} \sum_{i_2=1}^{B} \sum_{i_1=1}^{B} \overline{r}\left(i_1-i_2\right)^{q_1} \overline{r}\left(i_1-i_3\right)^{q_2} \overline{r}\left(i_1-i_4\right)^{q_3} \overline{r}\left(i_2-i_3\right)^{q_4} \overline{r}\left(i_2-i_4\right)^{q_5} \overline{r}\left(i_3-i_4\right)^{q_6}$$

$$\le \sum_{i_3=j}^{j+B-1} \sum_{i_4=j}^{j+B-1} \sum_{i_2=1}^{B} \overline{r}\left(i_2-i_3\right)^{q_4} \overline{r}\left(i_2-i_4\right)^{q_5} \overline{r}\left(i_3-i_4\right)^{q_6}$$

$$\times \prod_{k=1}^{2q} \left(\sum_{i_1=1}^{B} \overline{r}\left(i_1-i_k\right)^{q} \right)^{\frac{q_{k-1}}{q}}$$

$$\le C(D,q) M_B^q \sum_{i_3=j}^{j+B-1} \sum_{i_4=j}^{j+B-1} \overline{r}\left(i_3-i_4\right)^{q_6} \left(\left(B-i_3\right) \vee i_3\right)^{a q_2} \left(\left(B-i_4\right) \vee i_4\right)^{a q_3}$$

$$\cdot \left(\sum_{i_2=1}^{B} \overline{r}\left(i_2-i_3\right)^{q} \right)^{\frac{q_4}{q}} \left(\sum_{i_2=1}^{B} \overline{r}\left(i_2-i_4\right)^{q} \right)^{\frac{q_5}{q}} \left(\sum_{i_2=1}^{B} \left(\left(B-i_2\right) \vee i_2\right)^{aq} \right)^{\frac{q_1}{q}}$$

$$\le C(D,q) M_B^{2q} \left(j+B\right)^{d(q_1+q_3+q_5)} \sum_{i_3=j}^{j+B-1} \left(\left(B-i_3\right) \vee i_3\right)^{a(q_4+q_5)} \left(j+B-i_3\right)^{a q_6}$$

$$\le C(D,q) M_B^{2q} \left(j+B\right)^{2dq},$$

since $q_1 + \cdots + q_3 = 2m$ and $q_2 + q_4 + q_6 = m$ implies $q_1 + q_3 + q_5 = q$. Hence

$$\left(B d_B^4\right)^{-1} J_1 \le C(D,q) \left(B d_B^4\right)^{-1} \mathbb{N} M_B^{2q} \mathbb{N}^{2dq} = o(1)$$

implies that $\mathrm{Var}_B \left(\widehat{W}_{1,B}^2 \right) = o(1)$. Since $\mathrm{E}_B \left(\widehat{W}_{1,B}^2 \right) \to W_q^2$, Lemma S.4 follows.

□

Proof of Theorem 4: Let $W_*^2 = \frac{\mathrm{E}_B\left(\mathrm{Var}_B\left(\widehat{\beta}\right) - B^{-1}\widehat{\sigma}_\beta^2\right)}{d_B^2}$. Then, by using the Berry-Essen Theorem and Lahiri (1993), we can get

$$\sup_{x \in \mathbb{R}} \left| P_B \left(\sum_{i=1}^{B} \left\{ \left(\widehat{\beta}_i - \mu_\beta \right)^2 - B^{-1} \widehat{\sigma}_\beta^2 \right\} \le \sqrt{B} d_B w_q x \right) - \Phi\left(x\right) \right|$$

$$\le K \left[\widehat{\delta}_B \left(1 + \left(1 - B^{-1} - \widehat{\delta}_B \right)^{-1} \right) + \left(\beta + \widehat{\delta}_B \right) \left| 1 - B^{-1} - \widehat{\delta}_B \right|^{-\frac{3}{2}} \right]$$

with $\widehat{\delta}_B = \left(d_B W_*\right)^{-2} \mathrm{E}_B \left\{ \left(\widehat{\beta} - \mu_\beta \right)^2 - B^{-1} \widehat{\sigma}_\beta^2 \right\}^2 I \left\{ \left| \left(\widehat{\beta} - \mu_\beta \right)^2 - B^{-1} \widehat{\sigma}_\beta^2 \right| > C B^{\frac{1}{2}} d_B W_* \right\}$.

We shall now prove that $\widehat{\delta}_B \to 0$ in probability. Let $\mu_\beta = 0$ without loss of generality, by Lemma S.3 and Lemma S.4, it follows that $W_*^2 = \widehat{W}_B^2 + o_p(1) = W_q^2 + o_p(1)$, hence

$$\widehat{\delta}_B = o_p \left(\left(d_B W_q\right)^{-2} \mathrm{E}_B \left\{ \left(\widehat{\beta} - \mu_\beta \right)^2 \right\}^2 I \left\{ \left| \left(\widehat{\beta} - \mu_\beta \right)^2 \right| > C B^{\frac{1}{2}} d_B W_q \right\} \right) + o_p(1).$$

Now, using the arguments similar to those used in the proof of Lemma S.3, one can show that

$$\mathrm{E}\left(\Sigma_{j\in T} H_q\left(\widehat{\beta}_i\right)\right)^4 = o\left(d_B^4\right)$$

for $T = \{1, \cdots, B\}$. With $\overline{\beta} = \Sigma_{j\in T} c_q H_q\left(\widehat{\beta}_i\right)$, we have

$$\begin{aligned}
&(d_B W_q)^{-2} \mathrm{E}_B \left\{\left(\widehat{\beta} - \mu_\beta\right)^4\right\} I\left\{\left|\left(\widehat{\beta} - \mu_\beta\right)^2\right| > C B^{\frac{1}{2}} d_B W_q\right\} \\
&\leq 4\left(d_B W_q\right)^{-2}\left[\mathrm{E}_B\left(\overline{\beta}^4\right) I\left\{\overline{\beta}^2 > C B^{\frac{1}{2}} d_B W_q\right\} + \mathrm{E}\left(\Sigma_{j\in T}\widetilde{G}\left(\widehat{\beta}_j\right)\right)^2\right] = o_p(1).
\end{aligned}$$

As a result, $\widehat{\delta}_B = o_p(1)$, hence the theorem follows. □

Proof of Theorem 5*:* Following the proof of Theorem 3, the estimator $\widehat{\beta}$ could be rewritten as

$$\begin{aligned}
\widehat{\beta\eta} &= \frac{U^{\tau_2}\mathrm{E}\left(\widehat{Y}_{\mathrm{OLS}}\right) - U^{\tau_1}\mathrm{E}\left(\widehat{Y}_{\mathrm{OLS}}\right) - \left(\widehat{a\eta + c\alpha}\right)\left(U^{\tau_2}\mathrm{E}\left(\widehat{y}\right) - U^{\tau_1}\mathrm{E}\left(\widehat{y}\right)\right) - \widehat{\gamma} A_4}{A_1} \\
&= \frac{\left(\left(F'F\right)^{-1} F'\widehat{Y}\right)' i - Co\left(X'X\right)^{-1} X'y}{A_1},
\end{aligned}$$

where $F = (I\ S)_{r_\tau T\times 2}$, $\widehat{Y} = G\left(G'G\right)^{-1} G'\widehat{y}$, $G = (I\ \widehat{y})_{r_\tau T\times 2}$, $y = XB + E$ and $i = (0,1)'$ for model (17) in the main text. Substitute $y = XB + E$ into the above equation, by the definitions of Theorem 5, we will get

$$\begin{aligned}
&(t_S + \tau - 1)^H \frac{1}{\breve{A}_1} Co\left\{\left(X'X\right)^{-1} X'E\right\} \\
&= (t_S + \tau - 1)^H \frac{1}{\breve{A}_1} Co\left\{\frac{1}{r_\tau T}\sum_{s=1}^{r_\tau T} x_s x_s'\right\}^{-1}\left\{\frac{1}{r_\tau T}\sum_{s=1}^{r_\tau T} x_s e_s\right\}.
\end{aligned}$$

Then applying Corollary 2 of Wu & Zhou (2011), we will then get

$$\frac{1}{(t_S + \tau - 1)^H}\sum_{s=1}^{r_\tau T} x_s e_s \rightsquigarrow_d \mathbb{Q}(r_\tau T) - \mathbb{Q}(0). \tag{S.7}$$

Now consider $x_s x_s'$. Note that the process $x_s x_s' - \mathcal{M}_X\left(\frac{s}{(t_S+\tau-1)}\right)$ is mean zero and following the proof of Lemma 6 of Zhou & Wu (2010), we apply Doob's inequality to

$$\frac{1}{(t_S + \tau - 1)^H}\sum_{s=1}^{r_\tau T}\left(x_s x_s' - \mathcal{M}_X\left(\frac{s}{(t_S + \tau - 1)}\right)\right), \tag{S.8}$$

so that

$$\frac{1}{t_S+\tau-1}\sum_{s=1}^{r_\tau T} x_s x_s' \to_p \int_0^{r_\tau T}\mathcal{M}_X(s)ds \tag{S.9}$$

uniformly in r_τ. Combining (S.7) and (S.8), we finally get

$$(t_S+\tau-1)^H\frac{1}{\breve{A}_1}Co\left\{(X'X)^{-1}X'E\right\}\rightsquigarrow_d \frac{1}{\breve{A}_1}Co\left(\int_0^{r_\tau T}\mathcal{M}_X(s)ds\right)^{-1}(\mathbb{Q}(r_\tau T)-\mathbb{Q}(0)). \tag{S.10}$$

Similarly, we can get

$$\frac{1}{S'\widehat{y}-S'II'\widehat{y}}\widehat{y}'E=\frac{1}{\breve{A}_2}\widehat{y}'E\rightsquigarrow_d\left(\widetilde{\mathbb{Q}}(r_\tau T)-\widetilde{\mathbb{Q}}(0)\right) \tag{S.11}$$

and

$$\frac{\widehat{y}'I}{S'\widehat{y}-S'II'\widehat{y}}I'E=\frac{1}{\breve{A}_3}I'E\rightsquigarrow_d\left(\overline{\mathbb{Q}}(r_\tau T)-\overline{\mathbb{Q}}(0)\right). \tag{S.12}$$

Combining (S.10), (S.11), (S.12) and

$$\mathcal{B}ias(\tau)=\frac{1}{\breve{A}_1}CO\left((X'X)\right)^{-1}\left(X'\left(\widehat{\mathcal{B}}(\tau)-\mathcal{B}(\tau)\right)\right)$$

completes the proof of Theorem 5. □

References

1. Dobrushin R. L. and P. Major (1979). Non-central limit theorems for non-linear functional of Gaussian fields. *Zeitschrift für Wahrscheinlichkeits-theorie und Verwandte Gebiete* 50(1), 27-52.

2. Lahiri S. (1993) Refinements in asymptotic expansions for sums of weakly dependent random vectors. *The Annals of Probability* 791-799.

3. Matzkin R. (1992) Nonparametric estimation of nonadditive random functions. *Econometrica* 71, 1339–1375.

4. Rao C. R. and H. Toutenburg (1995) Linear Models: Least Squares and Alternatives. New York, NY: Springer.

5. Taqqu M. (1975) Weak convergence to fractional Brownian motion and to the Rosenblatt process. *Z. Wahrscheinlichkeitstheorie und Verw. Gebiete* 31:287–302.

6. Wu W. and Z. Zhou (2011) Gaussian Approximations for Non-Stationary Multiple Time Series. *Statistica Sinica* 21, 1397-1413.
