# Supplementary material for: Estimating endogenous treatments effects under long-range dependency without untreated controls
Source: PLoS One. 2026 Jun 3;21(6):e0347847. doi: 10.1371/journal.pone.0347847 (PMC13232959; doi:10.1371/journal.pone.0347847)
Supplement: S3 File — Detailed estimation procedure for panel model. (PDF) [file pone.0347847.s003.pdf]

Estimating Endogenous Treatments Effects under Long Range Dependency without Untreated Controls

SUPPLEMENT 3. Detailed Estimation Procedure for Panel Data Model

For model (24) with $h = 1$, we consider the unit-varying coefficients Bernstein expansion for the random variables $\widetilde{\Delta y_{i,t}} \equiv \widetilde{\Delta y}(i,t)$, $d_{i,t} \equiv d(i,t)$ of order 2:

$$\widetilde{\Delta y}(i,t) = \sum_{k=0}^{2} \frac{2!}{k!(2-k)!} \psi_{i,t}^{k} \left(1-\psi_{i,t}\right)^{2-k} f_{\widetilde{\Delta y}}\left(\frac{k}{2}\right) = \rho_{\widetilde{\Delta y},2,i}\psi_t^2 + \rho_{\widetilde{\Delta y},1,i}\psi_{i,t} + \rho_{\widetilde{\Delta y},0,i}, \tag{S.1}$$

$$d(i,t) = \sum_{k=0}^{2} \frac{2!}{k!(2-k)!} \psi_{i,t}^{k} \left(1-\psi_{i,t}\right)^{2-k} f_{d}\left(\frac{k}{2}\right) = \rho_{d,2,i}\psi_t^2 + \rho_{d,1,i}\psi_{i,t} + \rho_{d,0,i}, \tag{S.2}$$

where $f_\lambda(0) = 0$, $f_\lambda(\frac{1}{2}) = \frac{1}{2}\rho_{\lambda,1,i}$, $f_\lambda(1) = \rho_{\lambda,2,i} + \rho_{\lambda,1,i}$, the real numbers $\rho_{\lambda,2,i}, \rho_{\lambda,1,i} \in \mathbb{R} \setminus \{0\}$ are Bernstein coefficients and the expansions (S.1-S.2) we get are Bernstein polynomials of $\lambda(i,t)$ on $\psi_{i,t}$, $\lambda \in \{\widetilde{\Delta y}, d\}$. Note that the first order derivative of $\widetilde{\Delta y}(i,t)$, $d(i,t)$ are $\widetilde{\Delta y}'(i,t) = 2\rho_{\widetilde{\Delta y},2}\psi_{i,t} + \rho_{\widetilde{\Delta y},1}$ and $d'(i,t) = 2\rho_{d,2}\psi_{i,t} + \rho_{d,1}$ respectively for $i = 1,2,\ldots,N$, $t = 1,2,\ldots,t_{i,k}-1+h$. By the estimation Step 3.1, we know that there exists a continuous function $K(\cdot,\cdot) \in C^2(\mathbb{R})$ such that the relationship between $\widetilde{\Delta y}'(i,t)$ and $d'(i,t)$ satisfies *the balance condition*

$$K\left(\widetilde{\Delta y}'(i,t), d'(i,t)\right) = a_{d,2,i}\widetilde{\Delta y}'(i,t) - a_{\widetilde{\Delta y},2,i}d'(i,t) + a_{\widetilde{\Delta y},2,i}a_{d,1,i} - a_{d,2,i}a_{\widetilde{\Delta y},1,i} = 0,$$

where $a_{\lambda,q,i} = q\rho_{\lambda,q,i}$, $\rho_{\lambda,q,i}$ are Bernstein coefficients in (S.1-S.2), $q \in \{1,2\}$. Estimate (S.1-S.2) by varying coefficients panel methods (e.g. Su et al., 2019; Su et al., 2023; etc.) to get the estimators $\widehat{a}_{\lambda,q,i} = q\widehat{\rho}_{\lambda,q,i}$ for $q \in \{1,2\}$ and $\lambda \in \{\widetilde{\Delta y}, d\}$ respectively, and input all the estimators into the equation $K\left(\widetilde{\Delta y}'(i,t), d'(i,t)\right)$, we will get

$$\widehat{\widetilde{\Delta y}}'(i,t) = \frac{\widehat{a}_{\widetilde{\Delta y},2,i}d'(i,t) + \widehat{a}_{d,2,i}\widehat{a}_{\widetilde{\Delta y},1,i} - \widehat{a}_{\widetilde{\Delta y},2,i}\widehat{a}_{d,1,i}}{\widehat{a}_{d,2,i}}.$$

Further consider the following auxiliary unit-varying coefficients panel model

$$\widetilde{Y} = \widetilde{E} \circ \widetilde{B}_0 + \widetilde{X} \circ \widetilde{B}_1 + \widetilde{W}, \quad \text{with } y(i,t) \equiv \widehat{\widetilde{\Delta y}}'(i,t) \tag{S.3}$$

in which $\widetilde{E} = (I, I, \ldots, I)'_{N\times(t_{i,k}+h)}$ with $I = (1, 1, \ldots, 1)'_{(t_{i,k}+h)\times 1}$, the coefficient $\widetilde{B}_0 = (\delta_{01}, \delta_{01}, \ldots \delta_{0N})'_{N\times(t_{i,k}+h)}$ with $\delta_{0i} = (\delta_{0i,t=1}, \delta_{0i,t=2}, \ldots, \delta_{0i,t=t_k+h})'_{(t_{i,k}+h)\times 1}$, $\widetilde{X}$ is a $N\times(t_{i,k}+h)$ matrix collecting $y(i,t)$ for all i and t, $\widetilde{B} = (\delta_{11}, \delta_{11}, \ldots \delta_{1N})'_{N\times(t_{i,k}+h)}$ with $\delta_{1i} = (\delta_{1i,t=1}, \delta_{1i,t=2}, \ldots, \delta_{1i,t=t_k+h})'_{(t_{i,k}+h)\times 1}$, $\widetilde{W}$ is an unobservable random shock. $\widetilde{Y}$ is the $N \times (t_{i,k} + h)$ matrix collecting $\widetilde{\Delta y_{i,t}}$ in model (24) for all i and t, estimate model (S.3) by varying coefficients panel methods, we will then get

$$\widehat{\widetilde{Y}} = \widetilde{\mathbb{Z}}(\widetilde{\mathbb{Z}}'\widetilde{\mathbb{Z}})^{-1}\widetilde{\mathbb{Z}}'\widetilde{Y}, \qquad \widetilde{\mathbb{Z}} = (\widetilde{E}, \widetilde{X}). \tag{S.4}$$

Substitute (24) into (S.4), similar to step 3.1, we will then get

$$\begin{aligned}
&U^{\tau_2}E(\widehat{\widetilde{Y}}) - U^{\tau_1}E(\widehat{\widetilde{Y}}) \\
&\quad= \left(U^{\tau_2}E(\widehat{\widetilde{\Delta y}}'(i,*)) - U^{\tau_1}E(\widehat{\widetilde{\Delta y}}'(i,*))\right) \\
&\quad\times \left(\xi_i(h)\frac{U^\tau E(d_{i,*}\widehat{\widetilde{\Delta y}}'(i,*)) - U^\tau E(d_{i,*})U^\tau E(\widehat{\widetilde{\Delta y}}'(i,*))}{U^\tau E(\widehat{\widetilde{\Delta y}}'(i,*)\widehat{\widetilde{\Delta y}}'(i,*)) - U^\tau E(\widehat{\widetilde{\Delta y}}'(i,*))U^\tau E(\widehat{\widetilde{\Delta y}}'(i,*))}\right. \\
&\quad+ \alpha_i\frac{U^\tau E(\Delta X_{i,*}\widehat{\widetilde{\Delta y}}'(i,*)) - U^\tau E(\Delta X_{i,*})U^\tau E(\widehat{\widetilde{\Delta y}}'(i,*))}{U^\tau E(\widehat{\widetilde{\Delta y}}'(i,*)\widehat{\widetilde{\Delta y}}'(i,*)) - U^\tau E(\widehat{\widetilde{\Delta y}}'(i,*))U^\tau E(\widehat{\widetilde{\Delta y}}'(i,*))} \\
&\quad+ \gamma_i\frac{U^\tau E(\Delta Z_{i,*}\widehat{\widetilde{\Delta y}}'(i,*)) - U^\tau E(\Delta Z_{i,*})U^\tau E(\widehat{\widetilde{\Delta y}}'(i,*))}{U^\tau E(\widehat{\widetilde{\Delta y}}'(i,*)\widehat{\widetilde{\Delta y}}'(i,*)) - U^\tau E(\widehat{\widetilde{\Delta y}}'(i,*))U^\tau E(\widehat{\widetilde{\Delta y}}'(i,*))} \\
&\quad+ \left.\frac{U^\tau E((\Delta\nu_* + \Delta\varepsilon_{i,*})\widehat{\widetilde{\Delta y}}'(i,*)) - U^\tau E(\Delta\nu_* + \Delta\varepsilon_{i,*})U^\tau E(\widehat{\widetilde{\Delta y}}'(i,*))}{U^\tau E(\widehat{\widetilde{\Delta y}}'(i,*)\widehat{\widetilde{\Delta y}}'(i,*)) - U^\tau E(\widehat{\widetilde{\Delta y}}'(i,*))U^\tau E(\widehat{\widetilde{\Delta y}}'(i,*))}\right) \\
&\quad\equiv \xi_i(h)\breve{A}_1 + \alpha_i\breve{A}_2 + \gamma_i\breve{A}_3 + \breve{A}_4 \\
&\quad= \xi_i(h)\breve{A}_1 + \alpha_i\breve{A}_2 + \gamma_i\breve{A}_3
\end{aligned}$$

for $\tau_1 = \{1, 2, \ldots, t_{i,k} - 1\}$, $\tau_2 = \{t_{i,k} - 1 + h\}$ and $\tau = \{1, 2, \ldots, t_{i,k} - 1 + h\}$, $\xi_i(h) = \xi_{i,t_{i,k}-1+h}$ is the heterogeneous treatment effect for unit i at time $t_{i,k} - 1 + h$, the last equality holds true similar to Step 3.1. Notice that by Assumption 8, model (24) could be rewritten as

$$\widetilde{\Delta y_{i,t}} = (a\alpha_i + c)\, g\Big(\widetilde{\Delta y}'(i,t)\Big) + d_{i,t}\xi_{i,t} + \Delta Z_{i,t}\gamma_i + \tilde{b} + \tilde{d} + \Delta\varepsilon_{i,t} + u_{i,t} + o_p(1), \quad \text{(S.5)}$$

where cov $(d_{i,t}, \Delta\varepsilon_{i,t}) \neq 0$, $u_t = \tilde{u}_{i,t} + \tilde{w}_{i,t}$, $t = 1, 2, \ldots, T$. Estimate (S.5) by unit-varying coefficients semiparametric methods (e.g. Robinson, 1988; etc.), and by the exogeneity of $g\Big(\widetilde{\Delta y}'(i,t)\Big)$, under some mild conditions we will get $\widehat{(a\alpha_i + c)} = (a\alpha_i + c) + o_p(1)$ and $\widehat{\gamma}_i = \gamma_i + o_p(1)$ as $t_{i,k} \to \infty$. Substitute these estimators into $U^{\tau_2} E(\hat{\tilde{Y}}) - U^{\tau_1} E(\hat{\tilde{Y}})$, we will finally get

$$\widehat{\xi_i(h)} = \frac{U^{\tau_2} E(\hat{\tilde{Y}}) - U^{\tau_1} E(\hat{\tilde{Y}}) - \widehat{(a\alpha_i + c)} \left(U^{\tau_2} E(\widehat{\widetilde{\Delta y}}'(i,*)) - U^{\tau_1} E(\widehat{\widetilde{\Delta y}}'(i,*)) \right) - \widehat{\gamma}_i \check{A}_3}{\check{A}_1}.$$

Repeat the above procedure for $h = 2, 3, \ldots, T - t_{i,k}$, similar to Theorem 2, under some mild conditions we will finally get consistent estimates of the heterogeneous treatment effects $\widehat{\xi}_{i,t} = \xi_{i,t} + o_p(1)$ for $i = 1, 2, \ldots, N$ and $t = t_{i,k}, t_{i,k} + 1, \ldots, T$.
