# Supplementary material for: Estimating endogenous treatments effects under long-range dependency without untreated controls
Source: PLoS One. 2026 Jun 3;21(6):e0347847. doi: 10.1371/journal.pone.0347847 (PMC13232959; doi:10.1371/journal.pone.0347847)
Supplement: S4 File — Illustration of panel settings with staggered treatments adoptions. (PDF) [file pone.0347847.s004.pdf]

Estimating Endogenous Treatments Effects under Long Range Dependency without Untreated Controls

SUPPLEMENT 4. Illustration of Panel Settings with Staggered Treatments Adoptions

To capture treatments heterogeneity concerning the timings of treatments adoptions and provide the readers a more intuitive display of our definitions of panel settings as shown in (22). We now present two hypothetical illustration examples (Figure B (a) and (b)), in which we assume that there are 5 treated units with 6 phase states by Definition 6. For these two cases, the whole time interval $[1,T]$ could be divided into 6 phase states:

$$[t_{s=1}, t_{s=1}+1, t_{s=1}+2, t_{s=2}-1], \quad [t_{s=2}, t_{s=2}+1, t_{s=2}+2, t_{s=3}-1],$$
$$[t_{s=3}, t_{s=3}+1, t_{s=3}+2, t_{s=4}-1], \quad [t_{s=4}, t_{s=4}+1, t_{s=4}+2, t_{s=5}-1],$$
$$[t_{s=5}, t_{s=5}+1, t_{s=5}+2, t_{s=6}-1], \quad [t_{s=6}, t_{s=6}+1, t_{s=6}+2, T].$$

On the s-th phase state, unit i receives a treatment $\mathbb{d}_{i,s}$, s=1,2,...,6, i=1,2,...,5.

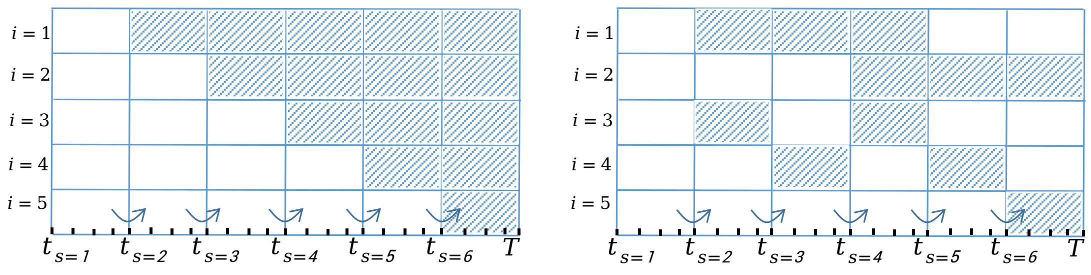

(a) Staggered rolls-in. (b) Staggered rolls-in and rolls-out.

Figure B. Staggered treatments adoptions. The horizontal rows correspond to time periods, and columns correspond to individual units, there are 5 individuals and the whole time interval $[1,T]$ can be divided into 6 phase states, $s = 1,2,...,6$. Panel (a) shows the situation where the start times of the treatments received by the individuals are different but the end times are same, while panel (b) shows the situation where the start times and end times are different.

The first example Figure B (a) illustrates the case where the start times of the treatments received by 5 individuals are different but the end times are the same. We refer to this situation "staggered rolls-in". Under this scenario, the complete treatments indicators for 5 units are

$$\mathbb{d}_{1,s=1} = (0,0,0,0,0)', \mathbb{d}_{2,s=1} = (0,0,0,0,0)', \mathbb{d}_{3,s=1} = (0,0,0,0,0)',$$

$\mathbb{d}_{4,s=1} = (0,0,0,0,0)'$, $\mathbb{d}_{5,s=1} = (0,0,0,0,0)'$, $\mathbb{d}_{1,s=2} = (1,1,1,1,1)'$,

$\mathbb{d}_{2,s=2} = (0,0,0,0,0)'$, $\mathbb{d}_{3,s=2} = (0,0,0,0,0)'$, $\mathbb{d}_{4,s=2} = (0,0,0,0,0)'$,

$\mathbb{d}_{5,s=2} = (0,0,0,0,0)'$, $\mathbb{d}_{1,s=3} = (1,1,1,1,1)'$, $\mathbb{d}_{2,s=3} = (1,1,1,1,1)'$,

$\mathbb{d}_{3,s=3} = (0,0,0,0,0)'$, $\mathbb{d}_{4,s=3} = (0,0,0,0,0)'$, $\mathbb{d}_{5,s=3} = (0,0,0,0,0)'$,

$\mathbb{d}_{1,s=4} = (1,1,1,1,1)'$, $\mathbb{d}_{2,s=4} = (1,1,1,1,1)'$, $\mathbb{d}_{3,s=4} = (1,1,1,1,1)'$,

$\mathbb{d}_{4,s=4} = (0,0,0,0,0)'$, $\mathbb{d}_{5,s=4} = (0,0,0,0,0)'$, ..., $\mathbb{d}_{1,s=6} = (1,1,1,1,1)'$

$\mathbb{d}_{2,s=6} = (1,1,1,1,1)'$, $\mathbb{d}_{3,s=6} = (1,1,1,1,1)'$, $\mathbb{d}_{4,s=6} = (1,1,1,1,1)'$,

$\mathbb{d}_{5,s=6} = (1,1,1,1,1)'$,

and the treatment indicator for all units on the first and second phase state is

$$\mathbb{D}_{*,s=1} = \begin{pmatrix} 0 & 0 & 0 & 0 & 0 \\ 0 & 0 & 0 & 0 & 0 \\ 0 & 0 & 0 & 0 & 0 \\ 0 & 0 & 0 & 0 & 0 \\ 0 & 0 & 0 & 0 & 0 \end{pmatrix}, \mathbb{D}_{*,s=2} = \begin{pmatrix} 1 & 1 & 1 & 1 & 1 \\ 0 & 0 & 0 & 0 & 0 \\ 0 & 0 & 0 & 0 & 0 \\ 0 & 0 & 0 & 0 & 0 \\ 0 & 0 & 0 & 0 & 0 \end{pmatrix},$$

the treatment indicator for all units on the third phase state is

$$\mathbb{D}_{*,s=3} = \begin{pmatrix} 1 & 1 & 1 & 1 & 1 \\ 1 & 1 & 1 & 1 & 1 \\ 0 & 0 & 0 & 0 & 0 \\ 0 & 0 & 0 & 0 & 0 \\ 0 & 0 & 0 & 0 & 0 \end{pmatrix},$$

and so forth, the treatment indicator for all units on the final phase state is

$$\mathbb{D}_{*,s=6} = \begin{pmatrix} 1 & 1 & 1 & 1 & 1 \\ 1 & 1 & 1 & 1 & 1 \\ 1 & 1 & 1 & 1 & 1 \\ 1 & 1 & 1 & 1 & 1 \\ 1 & 1 & 1 & 1 & 1 \end{pmatrix}.$$

The whole treatment indicator for all units on the whole phase states (whole time interval) for model (22) is

$$\mathbb{D}_{*,s=1} \oplus \mathbb{D}_{*,s=2} \oplus \mathbb{D}_{*,s=3} \oplus \mathbb{D}_{*,s=4} \oplus \mathbb{D}_{*,s=5} \oplus \mathbb{D}_{*,s=6}$$

$$= \begin{pmatrix} 0 & 0 & 0 & 0 & 0 & 1 & 1 & 1 & 1 & 1 & 1 & 1 & 1 & 1 & 1 & 1 & 1 & 1 & 1 & 1 & 1 & 1 & 1 & 1 & 1 & 1 & 1 & 1 & 1 & 1 \\ 0 & 0 & 0 & 0 & 0 & 0 & 0 & 0 & 0 & 0 & 1 & 1 & 1 & 1 & 1 & 1 & 1 & 1 & 1 & 1 & 1 & 1 & 1 & 1 & 1 & 1 & 1 & 1 & 1 & 1 \\ 0 & 0 & 0 & 0 & 0 & 0 & 0 & 0 & 0 & 0 & 0 & 0 & 0 & 0 & 0 & 1 & 1 & 1 & 1 & 1 & 1 & 1 & 1 & 1 & 1 & 1 & 1 & 1 & 1 & 1 \\ 0 & 0 & 0 & 0 & 0 & 0 & 0 & 0 & 0 & 0 & 0 & 0 & 0 & 0 & 0 & 0 & 0 & 0 & 0 & 0 & 1 & 1 & 1 & 1 & 1 & 1 & 1 & 1 & 1 & 1 \\ 0 & 0 & 0 & 0 & 0 & 0 & 0 & 0 & 0 & 0 & 0 & 0 & 0 & 0 & 0 & 0 & 0 & 0 & 0 & 0 & 0 & 0 & 0 & 0 & 0 & 1 & 1 & 1 & 1 & 1 \end{pmatrix}.$$

The second example Figure B (b) illustrates the case where the start times of the treatments received by 5 individuals are different and the end times are also different. We refer to this situation “staggered rolls-in and rolls-out”. Under this scenario, the

complete treatments indicators for 5 units are

$$\mathbb{d}_{1,s=1} = (0,0,0,0,0)', \mathbb{d}_{2,s=1} = (0,0,0,0,0)', \mathbb{d}_{3,s=1} = (0,0,0,0,0)',$$

$$\mathbb{d}_{4,s=1} = (0,0,0,0,0)', \mathbb{d}_{5,s=1} = (0,0,0,0,0)', \mathbb{d}_{1,s=2} = (1,1,1,1,1)',$$

$$\mathbb{d}_{2,s=2} = (0,0,0,0,0)', \mathbb{d}_{3,s=2} = (1,1,1,1,1)', \mathbb{d}_{4,s=2} = (0,0,0,0,0)',$$

$$\mathbb{d}_{5,s=2} = (0,0,0,0,0)', \mathbb{d}_{1,s=3} = (1,1,1,1,1)', \mathbb{d}_{2,s=3} = (0,0,0,0,0)',$$

$$\mathbb{d}_{3,s=3} = (0,0,0,0,0)', \mathbb{d}_{4,s=3} = (1,1,1,1,1)', \mathbb{d}_{5,s=3} = (0,0,0,0,0)',$$

$$\mathbb{d}_{1,s=4} = (1,1,1,1,1)', \mathbb{d}_{2,s=4} = (1,1,1,1,1)', \mathbb{d}_{3,s=4} = (1,1,1,1,1)',$$

$$\mathbb{d}_{4,s=4} = (0,0,0,0,0)', \mathbb{d}_{5,s=4} = (0,0,0,0,0)', \ldots, \mathbb{d}_{1,s=6} = (0,0,0,0,0)'$$

$$\mathbb{d}_{2,s=6} = (1,1,1,1,1)', \mathbb{d}_{3,s=6} = (0,0,0,0,0)', \mathbb{d}_{4,s=6} = (0,0,0,0,0)',$$

$$\mathbb{d}_{5,s=6} = (1,1,1,1,1)',$$

and the treatment indicator for all units on the first and second phase state is

$$\mathbb{D}_{*,s=1} = \begin{pmatrix} 0 & 0 & 0 & 0 & 0 \\ 0 & 0 & 0 & 0 & 0 \\ 0 & 0 & 0 & 0 & 0 \\ 0 & 0 & 0 & 0 & 0 \\ 0 & 0 & 0 & 0 & 0 \end{pmatrix}, \mathbb{D}_{*,s=2} = \begin{pmatrix} 1 & 1 & 1 & 1 & 1 \\ 0 & 0 & 0 & 0 & 0 \\ 1 & 1 & 1 & 1 & 1 \\ 0 & 0 & 0 & 0 & 0 \\ 0 & 0 & 0 & 0 & 0 \end{pmatrix},$$

the treatment indicator for all units on the third phase state is

$$\mathbb{D}_{*,s=3} = \begin{pmatrix} 1 & 1 & 1 & 1 & 1 \\ 0 & 0 & 0 & 0 & 0 \\ 0 & 0 & 0 & 0 & 0 \\ 1 & 1 & 1 & 1 & 1 \\ 0 & 0 & 0 & 0 & 0 \end{pmatrix},$$

and so forth, the treatment indicator for all units on the final phase state is

$$\mathbb{D}_{*,s=6} = \begin{pmatrix} 0 & 0 & 0 & 0 & 0 \\ 1 & 1 & 1 & 1 & 1 \\ 0 & 0 & 0 & 0 & 0 \\ 0 & 0 & 0 & 0 & 0 \\ 1 & 1 & 1 & 1 & 1 \end{pmatrix}.$$

The whole treatment indicator for all units on the whole phase states (whole time interval) for model (22) is

$$
\mathbb{D}_{*,s=1} \bigoplus \mathbb{D}_{*,s=2} \bigoplus \mathbb{D}_{*,s=3} \bigoplus \mathbb{D}_{*,s=4} \bigoplus \mathbb{D}_{*,s=5} \bigoplus \mathbb{D}_{*,s=6}
$$

$$
= \begin{pmatrix}
0 & 0 & 0 & 0 & 0 & 1 & 1 & 1 & 1 & 1 & 1 & 1 & 1 & 1 & 1 & 1 & 1 & 1 & 1 & 1 & 0 & 0 & 0 & 0 & 0 & 0 & 0 & 0 & 0 & 0 \\
0 & 0 & 0 & 0 & 0 & 0 & 0 & 0 & 0 & 0 & 0 & 0 & 0 & 0 & 0 & 1 & 1 & 1 & 1 & 1 & 1 & 1 & 1 & 1 & 1 & 1 & 1 & 1 & 1 & 1 \\
0 & 0 & 0 & 0 & 0 & 1 & 1 & 1 & 1 & 1 & 0 & 0 & 0 & 0 & 0 & 1 & 1 & 1 & 1 & 1 & 0 & 0 & 0 & 0 & 0 & 0 & 0 & 0 & 0 & 0 \\
0 & 0 & 0 & 0 & 0 & 0 & 0 & 0 & 0 & 0 & 1 & 1 & 1 & 1 & 1 & 0 & 0 & 0 & 0 & 0 & 1 & 1 & 1 & 1 & 1 & 0 & 0 & 0 & 0 & 0 \\
0 & 0 & 0 & 0 & 0 & 0 & 0 & 0 & 0 & 0 & 0 & 0 & 0 & 0 & 0 & 0 & 0 & 0 & 0 & 0 & 0 & 0 & 0 & 0 & 0 & 1 & 1 & 1 & 1 & 1
\end{pmatrix}.
$$
