# Supplementary material for: Estimating endogenous treatments effects under long-range dependency without untreated controls
Source: PLoS One. 2026 Jun 3;21(6):e0347847. doi: 10.1371/journal.pone.0347847 (PMC13232959; doi:10.1371/journal.pone.0347847)
Supplement: S5 File — Impact of choosing a weak or invalid CP variable on estimation and inference. (PDF) [file pone.0347847.s005.pdf]

Estimating Endogenous Treatments Effects under Long Range Dependency without Untreated Controls

SUPPLEMENT 5. Impact of Choosing a Weak or Invalid CP on Estimation and Inference

Within the CMS framework of the paper, the Common Proximal Variable (CP) ψ must satisfy two core conditions: **(1) it is highly correlated with the unobservable confounders (W, X); and (2) it is independent of the model error term ϵ.** If the chosen CP is "weak" (i.e., has low correlation with the confounders) or "invalid" (i.e., is correlated with ϵ), the estimator will be biased, and inference will be invalid. The following mathematical derivation illustrates the specific impacts.

For simplicity, consider a single-treatment model with S (ignoring D and Z):

$$y_t = S_t\beta + W_t\eta + \varepsilon_t, \quad \text{cov}(S_t, \varepsilon_t) \neq 0, \tag{S.1}$$

where W_t is an unobservable confounder. Assume the relationship between W_t and the CP variable ψ_t is:

$$W_t = \theta\psi_t + u_t, \quad E(u_t) = 0, \quad \text{cov}(\psi_t, u_t) = 0, \tag{S.2}$$

Here, θ measures the strength of the CP ($\theta = 0$ implies it is completely irrelevant). We allow ψ_t to be potentially correlated with ε_t, denoting the covariance as $\sigma_{\psi\varepsilon} = \text{cov}(\psi_t, \varepsilon_t)$. The CP is valid when $\sigma_{\psi\varepsilon} = 0$ and invalid otherwise.

I. A Simplified Representation of the Two-Step Estimation Procedure

The paper's estimation procedure (Step 3.1) can be simplified into the following key steps:

1. Using Bernstein expansions, construct an auxiliary variable $\tilde{y}'_t$. Under a valid CP, $\tilde{y}'_t$ is proportional to W_t.

2. Run an auxiliary regression: $y_t = \delta_0 + \delta_1\tilde{y}'_t + \text{error}$, obtaining the fitted values $\hat{y}_t^{\text{OLS}}$.

3. Isolate β by taking the difference in means between the post-treatment and pre-treatment subsamples:

$$\hat{\beta} = \frac{\bar{y}_{\text{post}}^{\text{OLS}} - \bar{y}_{\text{pre}}^{\text{OLS}} - \hat{\eta}(\bar{\tilde{y}}'_{\text{post}} - \bar{\tilde{y}}'_{\text{pre}})}{A_1}, \tag{S.3}$$

   where $\bar{y}_{\text{post}}^{\text{OLS}}$ represents the mean of $\hat{y}_t^{\text{OLS}}$ in the post-treatment subsample, $\hat{\eta}$ is the estimator for η, and A_1 is a non-zero constant determined by the data.

Under a valid CP, it can be shown that $\hat{\beta} \xrightarrow{p} \beta$.

II. Impact of a Weak CP (θ is close to zero but non-zero, and ψ is independent of ϵ)

Assume $\sigma_{\psi\varepsilon} = 0$, but θ is very small. In this case, ψ_t is only weakly correlated with W_t. From (S.2), only a small fraction of the variation in W_t can be explained by ψ_t, with the majority coming from u_t.

In the first step of constructing $\tilde{y}'_t$, it primarily reflects the information in ψ_t and largely ignores u_t. Consequently, $\tilde{y}'_t$ is weakly related to W_t and approximately independent of u_t.

Let $\tilde{y}'_t = c\psi_t + o_p(1)$, where c is some constant. The fitted values $\hat{y}_t^{\text{OLS}}$ from the auxiliary regression will primarily depend on ψ_t, even though the true y_t includes the u_t term. The numerator in equation (S.3) can then be decomposed as:

$$\bar{y}_{\text{post}}^{\text{OLS}} - \bar{y}_{\text{pre}}^{\text{OLS}} = c(\bar{\psi}_{\text{post}} - \bar{\psi}_{\text{pre}}) + o_p(1).$$

Similarly, $\bar{\tilde{y}}'_{\text{post}} - \bar{\tilde{y}}'_{\text{pre}} = c(\bar{\psi}_{\text{post}} - \bar{\psi}_{\text{pre}}) + o_p(1)$. Substituting into (S.3) gives:

$$\hat{\beta} = \frac{c(\bar{\psi}_{\text{post}} - \bar{\psi}_{\text{pre}}) - \hat{\eta}c(\bar{\psi}_{\text{post}} - \bar{\psi}_{\text{pre}}) + o_p(1)}{A_1} = \frac{c(1-\hat{\eta})(\bar{\psi}_{\text{post}} - \bar{\psi}_{\text{pre}})}{A_1} + o_p(1). \tag{S.4}$$

Since θ is very small, c is also small (because $c \propto \theta$), and the estimate $\hat{\eta}$ may be unstable. Crucially, the true β does not appear in (4); it is replaced by terms that converge to zero. In fact, as $\theta \to 0$, $c \to 0$, the numerator tends to zero, and the denominator A_1 also tends to zero (as A_1 depends on the information in ψ), leading to an indeterminate limiting behavior for $\hat{\beta}$. More precisely, it can be shown that the asymptotic variance of $\hat{\beta}$ is proportional to $1/\theta^2$:

$$\sqrt{T}(\hat{\beta} - \beta) \xrightarrow{d} N\left(0, \frac{\sigma^2}{\theta^2} \cdot \Sigma\right), \quad \text{as long as } \theta \neq 0,$$

where σ^2 is the error variance and Σ is some positive definite matrix. Therefore, the smaller θ is, the larger the variance and the lower the estimation precision. When $\theta = 0$, the model is unidentified, and $\hat{\beta}$ does not converge to a constant.

III. Impact of an Invalid CP (ψ is correlated with ϵ)

Now suppose $\sigma_{\psi\varepsilon} \neq 0$, even though θ might be large. In this case, ψ_t is correlated with ε_t. Consequently, the constructed $\tilde{y}'_t$ in the first step will also contain information from ε_t, making the fitted values $\hat{y}_t^{\text{OLS}}$ from the auxiliary regression correlated with ε_t. Consider the numerator in equation (S.3):

$$\bar{y}_{\text{post}}^{\text{OLS}} - \bar{y}_{\text{pre}}^{\text{OLS}} = \beta(\bar{S}_{\text{post}} - \bar{S}_{\text{pre}}) + \eta(\bar{W}_{\text{post}} - \bar{W}_{\text{pre}}) + (\bar{\varepsilon}_{\text{post}} - \bar{\varepsilon}_{\text{pre}}) + \text{estimation error terms}.$$

Because $\tilde{y}'_t$ is correlated with ε, the estimator $\hat{\eta}$ will be inconsistent. Let its probability limit be $\eta^* \neq \eta$. Furthermore, $\bar{y}_{\text{post}}^{\text{OLS}} - \bar{y}_{\text{pre}}^{\text{OLS}}$ contains the difference in ε, which is correlated with ψ and hence with $\bar{\tilde{y}}'_{\text{post}} - \bar{\tilde{y}}'_{\text{pre}}$. Ultimately, the bias in $\hat{\beta}$ can be expressed as:

$$\hat{\beta} \xrightarrow{p} \beta + \frac{\eta\Delta W + \Delta\varepsilon - \eta^*\Delta\tilde{y}'}{A_1^*}, \tag{S.5}$$

where $\Delta W = \text{plim}(\bar{W}_{\text{post}} - \bar{W}_{\text{pre}})$, $\Delta\varepsilon$ and $\Delta\tilde{y}'$ are defined similarly, and A_1^* is the probability limit of A_1. Since ψ is correlated with ε, $\Delta\varepsilon$ is correlated with $\Delta\tilde{y}'$, and since $\eta^* \neq \eta$, the second term in (S.5) is non-zero, resulting in asymptotic bias.

Specifically, if the correlation coefficient between ψ and ε is ρ, the bias term is proportional to ρ. The bias disappears when $\rho = 0$ (the valid case). Therefore, an invalid CP leads to an inconsistent estimator, with the magnitude of the bias depending on the degree of correlation between ψ and ε.

IV. Impact on Inference

1. **Weak CP**: Although the estimator may still be consistent (when $\theta \neq 0$ and ψ is independent of ϵ), the variance inflation leads to overly wide confidence intervals and reduced test power. Moreover, significant bias may be present in finite samples. In bootstrap inference, due to the large variance, an extremely large number of bootstrap samples is required to obtain stable results.

2. **Invalid CP**: The estimator is inconsistent, causing the center of the confidence interval to shift and the coverage probability to fall below the nominal level. Even bootstrap quantile methods cannot correct this systematic bias, as the center of the distribution is displaced from the true value.

V. Summary

CP Type	Mathematical Manifestation	Impact on Estimation	Impact on Inference
**Weak**	Small θ, but $\sigma_{\psi\varepsilon} = 0$	Variance $\propto 1/\theta^2$, consistent asymptotically but slow convergence; potentially biased in finite samples	Confidence intervals are too wide, low test power; bootstrap requires very large samples
**Invalid**	$\sigma_{\psi\varepsilon} \neq 0$	Estimator is inconsistent, bias proportional to ρ	Confidence interval center is shifted, coverage probability is distorted; any inference method fails

In practical applications, the choice of CP must be made cautiously. It is crucial to prioritize, based on background theory, ensuring its independence from the error term and, where possible, selecting a CP strongly correlated with the confounders to avoid the problems outlined above.
